# HMPA-Catalyzed Transfer Hydrogenation of 3-Carbonyl Pyridines and Other *N*-Heteroarenes with Trichlorosilane

**DOI:** 10.3390/molecules24030401

**Published:** 2019-01-22

**Authors:** Yun Fu, Jian Sun

**Affiliations:** 1 Chengdu Institute of Biology, Chinese Academy of Sciences, Chengdu 610041, China; fy860401@163.com; 2 University of Chinese Academy of Sciences, Beijing 100049, China

**Keywords:** HMPA, transfer hydrogenation, trichlorosilane, pyridines

## Abstract

A method for the HMPA (hexamethylphosphoric triamide)-catalyzed metal-free transfer hydrogenation of pyridines has been developed. The functional group tolerance of the existing reaction conditions provides easy access to various piperidines with ester or ketone groups at the C-3 site. The suitability of this method for the reduction of other *N*-heteroarenes has also been demonstrated. Thirty-three examples of different substrates have been reduced to designed products with 45–96% yields.

## 1. Introduction

Piperidines are very important structural building blocks of numerous biologically active compounds, such as the Topo inhibitor, the Chk1 inhibitor, Tiagabine and Focalin XR [1]. The catalytic hydrogenation of pyridines provides one of the most straightforward methods to access piperidines [2,3,4,5,6,7,8,9,10,11,12,13,14,15,16,17,18], although it is essential to overcome some inherent challenges presented by catalyst deactivation and pyridine dearomatization. In the last decade, various transition-metal catalyst systems have been studied for the direct hydrogenation of pyridines, but the metal-free catalytic reduction of pyridines is a great challenge [19,20,21,22,23,24]. 

**Figure 1:**
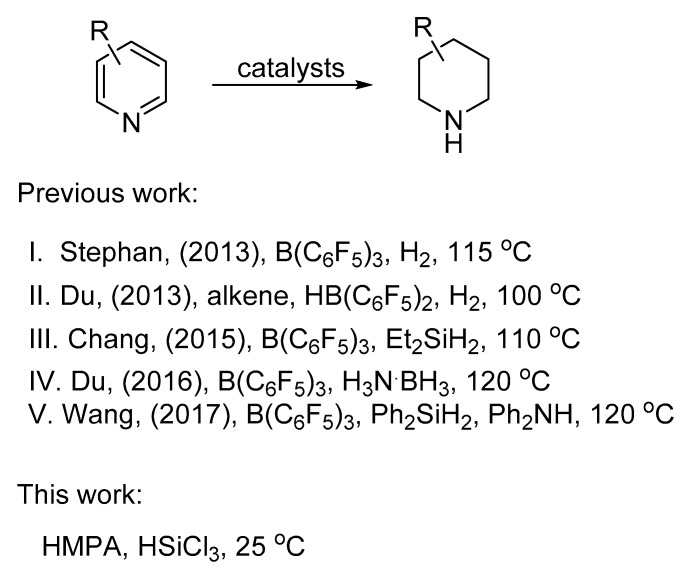
Selected examples for the synthesis of piperidines through the catalytic reduction of pyridines.

In recent years, frustrated Lewis base pairs have been proven as efficient catalyst systems for the regio- and chemoselective reduction of pyridines with various reducing agents. In particular, Stephan [25] and Du [26], respectively, reported the metal-free organoborane-catalyzed hydrogenation of pyridines with H_2_. Later on, Du [27] developed a method for the metal-free organoborane catalyzed transfer hydrogenation of pyridines with ammonia boranes. Chang [28] and Wang [29] reported the B(C_6_F_5_)_3_ catalyzed reduction of pyridines with Et_2_SiH_2_ and PhMe_2_SiH, respectively (Figure 1). For the reduction of the 3-carbonyl pyridines, Rueping reported the first example of organocatalytic transfer hydrogenation of 3-carbonyl pyridines with Hantzsch ester for the preparation of chiral 1,4-dihydropyridine (DHP) derivatives [30]. Although a variety of 3-carbonyl piperidines derivatives could be prepared with these methods, there are still some drawbacks: (1) For most of these reactions, a high temperature and (2) high pressure of H_2_ was required. Therefore, the search for new methods for the reduction of 3-carbonyl pyridines still remains a challenging task.

Trichlorosilane with a Lewis base as an activator is a well-known unsaturated double bond reduction method. Its strength has been well demonstrated by others and ourselves in terms of asymmetric reduction of double bonds [31,32,33,34,35,36,37,38]. However, to the best of our knowledge, the reduction of pyridines by use of this system still presents a great challenge, and no successful protocol has been reported. In our previous study, we found trichlorosilane could activate the imine substrate through coordination of the nitrogen atom. A similar coordination was also found when trichlorosilane and pyridines were added together. Thus, we envisioned that pyridines would be reduced by trichlorosilane with a proper Lewis base activator. Here in, we wish to communicate the results of our study and present a highly effective new method to reduce 3-carbonyl pyridines under an organic Lewis base activated trichlorosilane system.

## 2. Results and Discussion

To implement our design, phenyl(pyridin-3-yl)methanone **1a** was used as a model substrate to test the catalytic activity of various commercially available Lewis bases. We found that the reduced product could be obtained with 49% and 37%, respectively, when 0.2 equivalent of HMPA (hexamethylphosphoric triamide) or POPh_3_ were used. However, only a trace amount of reduced product could be detected when DMF (*N*,*N*-dimethylformamide) was used, which has been proven as an efficient catalyst for the reduction of C=O and C=N bonds with trichlorosilane. Dichloromethane was the most suitable solvent for this reaction. An 86% yield could be obtained when six equivalents of trichlorosilane were added as a reducing agent. The yield could be increased further to 96% when the reaction was stirred at 25 °C for 24 h. Decreasing the amount of HMPA to 10 mol% and 5 mol%, respectively, both caused a clear drop in the yield. After a careful investigation, we identified the best reaction conditions in which the substrate **1a** was reduced with trichlorosilane (6.0 equivalent) under the catalysis of HMPA (20 mol%) in DCM (dichloromethane) at 25 °C for 24 h (Table 1).

**Figure 2:**
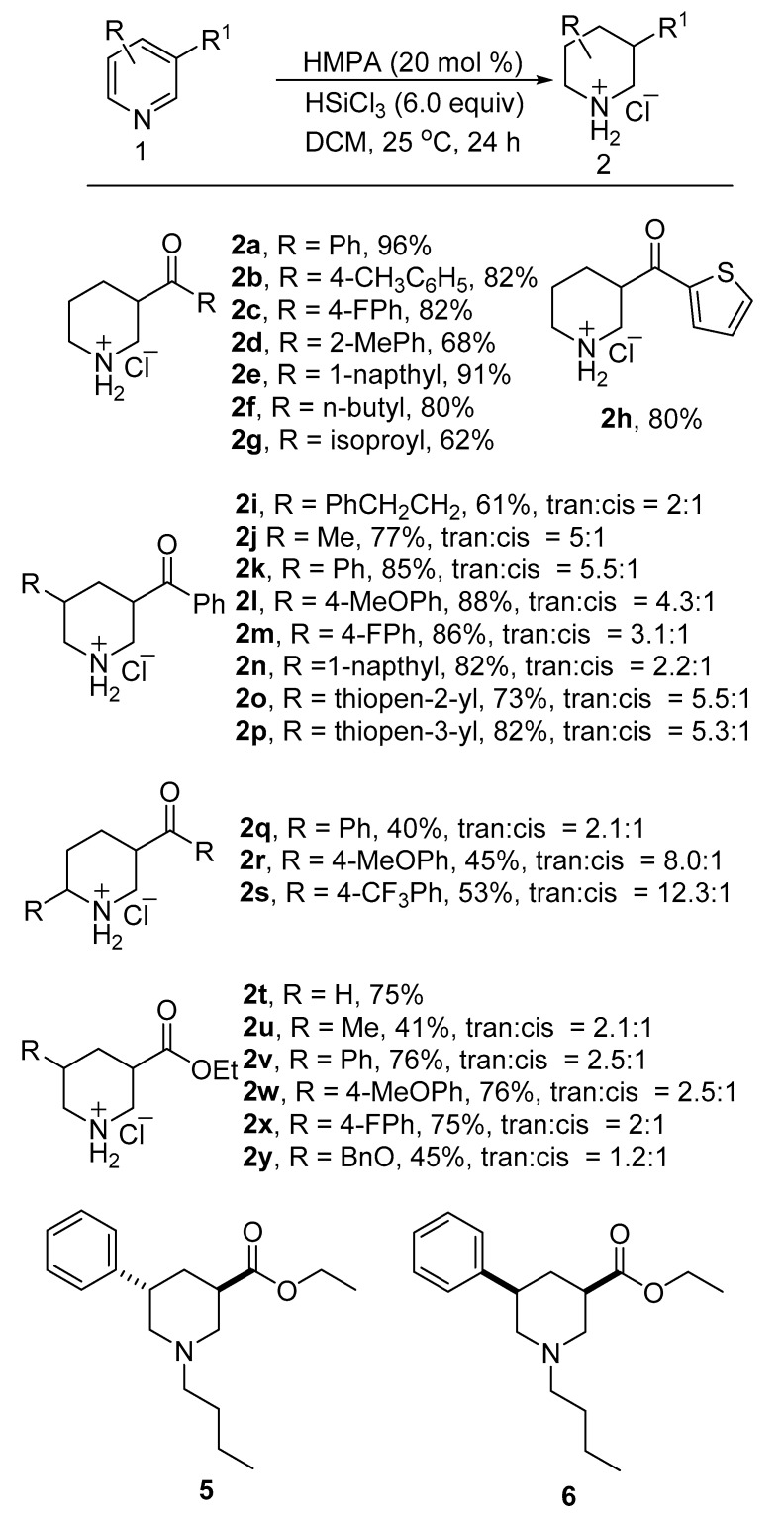
Substrate scope of the HMPA-catalyzed reduction of pyridines.

With the optimized reaction conditions in hand, the scope and limitations for the substrates were investigated. We found a series of phenyl(pyridin-3-yl)methanone derivatives could be reduced under the existing reaction conditions to get the desired product with good yields (Figure 2). The desired products **2b**–**2h** were obtained with 62–91% yields when the phenyl group of phenyl(pyridin-3-yl)methanone was replaced with other aryl and alkyl substituents. The 3,5-disubstituted pyridines could also be reduced under the existing reaction conditions. The substituents of the 5-position of the pyridine ring could be aryl and alkyl groups. The 5-phenethyl and 5-methyl substituted substrates could be reduced to the desired products **2i** and **2j** with 61% and 77% yields, respectively. When the 5-position substituent groups of pyridines were Ph, 4-MePh, 4-FPh and 1-napthyl, these pyridines could be reduced to 3,5-disubsitituted piperidines with 82–88% yields. The substrates with a hetero aromatic group at the 5-position of the pyridines are also tolerated. The desired products **2o** and **2p** were obtained with 73% and 82% yields, respectively, when the thiopen-2-yl and thiopen-3-yl substituted substrates were reduced under the existing reaction conditions. Next, we found that the 3,6-disubstituted substrates could be reduced with moderate yields. The desired reducing products **2q**–**2s** could be obtained with 40–53% yields.

Ethyl nicotinate and its 5-position substituted derivatives are tolerated under the existing reaction conditions. Ethyl nicotinate was reduced to the corresponding product **2t** with a 75% yield. The 5-methly substituted ethyl nicotinate was reduced with a 41% yield, and the 5-Ph, 4-MeOPh and 4-FPh substituted ethyl nicotinate were reduced to their corresponding products **2v**–**2x** with 75–76% yields. The 5-BnO substituted ethyl nicotinate could also be reduced to the corresponding product **2y** with a 45% yield. In order to confirm the relative configuration of the main product, compounds **5** and **6** were synthesized according the literature, and the trans product was confirmed to be the main product [39].

**Figure 3:**
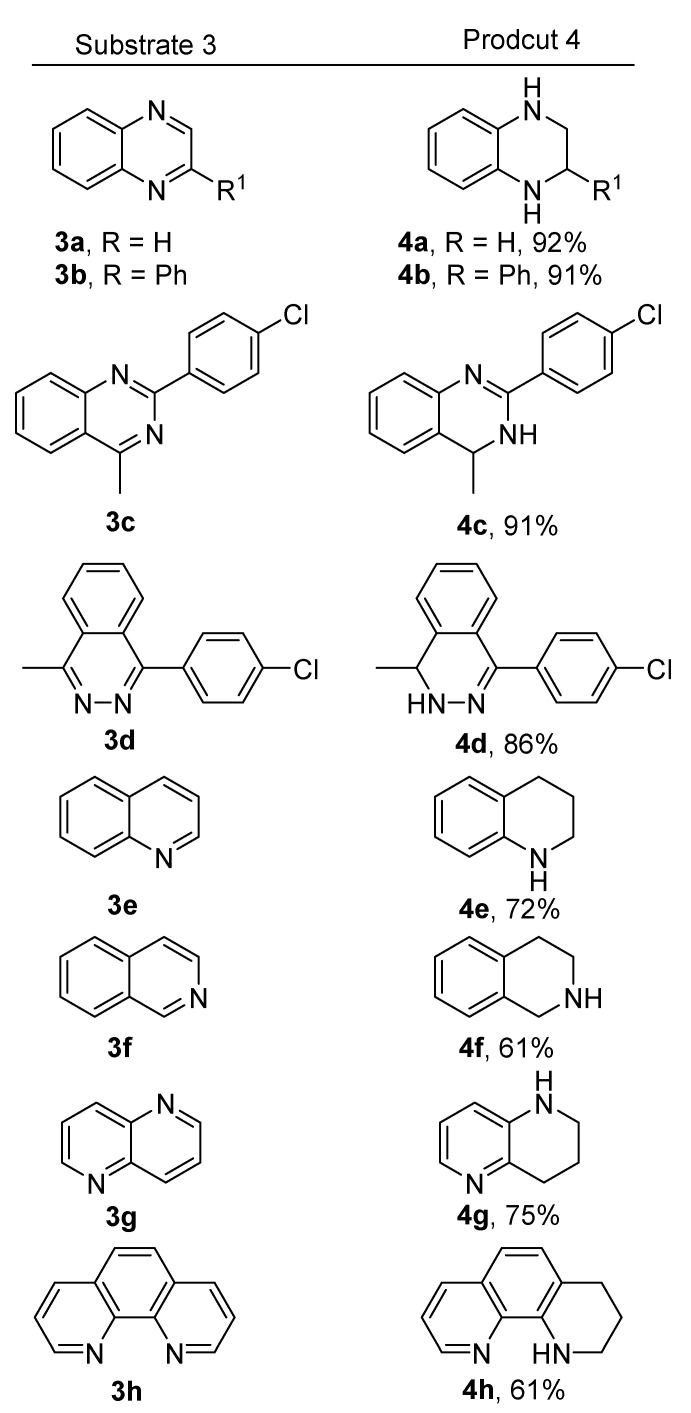
Substrate scope of the HMPA-catalyzed reduction of *N*-heteroarenes.

Pyridine derivatives such as 3-Br, 3-CF_3_, 3-NO_2_, and 3-CN substituted pyridines could not be reduced under the existing reaction conditions. However, other *N*-heteroarenes such as quinoxaline (**3a**) and 2-phenylquinoxaline (**3b**) could be reduced to their tetrahydroquinoxaline derivatives **4a** [29] and **4b** [40] with high yields. The substrates **3c** and **3d** were partially reduced to the products **4c** and **4d.** Quinolone (**3e**) and isoquinoline (**3f**) were reduced to products **4e** [41] and **4f** [41], respectively, with moderate yields. 1,5-Naphthyridine (**3g**) and 1,10-phenanthroline (**3h**) could only be partially reduced to the products **4g** [42] and **4h** [43] (Figure 3). All attempts to achieve the fully reduced products of **3c**, **3d**, **3g** and **3h** have failed. 

**Figure 4:**
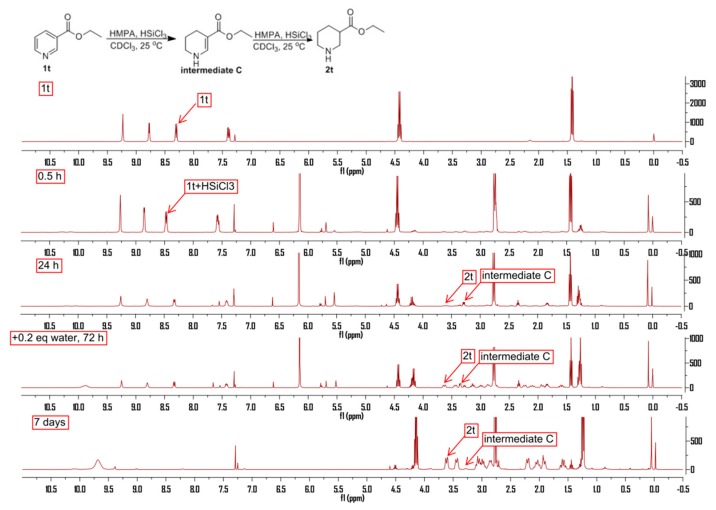
^1^H-NMR spectra for mechanism studies.

In order to shed light on the mechanistic pathway, in situ NMR analysis was performed. We first found that ethyl nicotinate could form a complex with HSiCl_3_ when the reaction was set in CDCl_3_ under the otherwise identical reaction conditions. An obvious chemical shift in the aromatic region could be detected when HSiCl_3_ was added to the solution of **1t**. Besides the signals of the designed products, a group of peaks that matched the intermediate **C** were also detected at the beginning. The intensity of these peaks would decrease with the addition of water. At the end of the reaction, only the peaks of designed product and HMPA could be detected (Figure 4).

**Figure 5:**
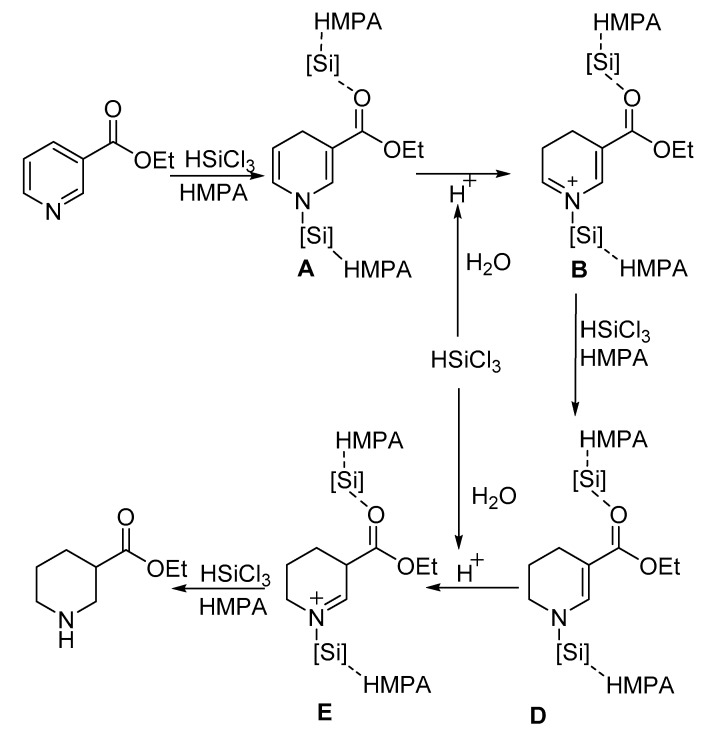
Proposed mechanism of HMPA-catalyzed reduction of ethyl nicotinate with HSiCl_3_.

Based on the above observations and precedents indicating a stepwise process in the reduction of unsaturated pyridines [27], we proposed a possible mechanistic pathway for the present HMPA-catalyzed reduction of pyridines (Figure 5). The first step was assumed to be the formation of a HSiCl_3_ and substrate complex, followed by the hydride attack at the C-4 position to produce the 1,4-dihydropyidine intermediate, **A**, which will transfer to **B** in the presence of a proton, and then be reduced to **D** with HSiCl_3_ under the catalysis of HMPA. The **D** to **E** step is the rate-determining step, since only the intermediate **D** could be detected and isolated from the reaction. The proton which is coming from the hydrochloride that is formed by the hydrolysis of HSiCl_3_ is important for the existing reaction. 

**Figure 6:**
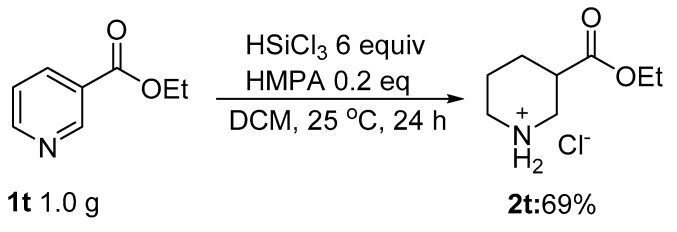
The gram-scale reaction of **1t**.

In order to illustrate the synthetic potential of these methodologies, a gram-scale reaction was carried out using **1t** as the substrate. Fortunately, the desired product, **2t**, was obtained in a yield of 69% (Figure 6).

## 3. Materials and Methods

All solvents used in the reactions were distilled from the appropriate drying agents prior to use. All substrates were analogously prepared and characterized as reported in the Supplementary Materials. Reactions were monitored by thin layer chromatography, using silica gel HSGF254 plates. Flash chromatography was performed using silica gel HG/T2354-92. ^1^H- and ^13^C-NMR (400 and 100 MHz, respectively) spectra were recorded in CDCl_3_. The ^1^H-NMR chemical shifts are reported in ppm (δ) relative to tetramethylsilane (TMS), with the solvent resonance employed as the internal standard (CDCl_3_, δ 7.26 ppm). Data are reported as follows: Chemical shift, multiplicity (s = singlet, d = doublet, t = triplet, q = quartet, m = multiplet, br = broad, dd = double doublet), coupling constants (Hz) and integration. ^13^C-NMR chemical shifts are reported in ppm from tetramethylsilane (TMS) with the solvent resonance as the internal standard (CDCl_3_, δ 77.0 ppm). ESIMS (Electron Spray Ionization Mass Spectrometry) spectra were recorded on BioTOF Q (Bruker, Billerica, MA, USA).

### 3.1. Reduction of Pyridines and N-Heteroaromatics

Under an argon atmosphere, pyridine **1** or *N*-heteroaromatic **3** (0.10 mmol) and HMPA (3.5 mg, 0.02 mmol) were added in anhydrous DCM (0.7 mL) and stirred at room temperature for 10 min, and then trichlorosilane (2.0 M, 0.3 mL) was added. The reaction was stirred at room temperature for 24 h, quenched with H_2_O, and then the pH was adjusted to ~7–8 with saturated NaHCO_3_. The mixture was extracted with EtOAc (3 × 5 mL). The combined organic layers were washed with brine, dried over anhydrous Na_2_SO_4_, concentrated under reduced pressure and purified with column chromatography (silica gel, DCM/MeOH/TEA = 10/1/0.1) to afford a pure product.

*3-Benzoylpiperidin-1-ium Chloride* (**2a**). Colorless oil. ^1^H-NMR (400 MHz, CDCl_3_): δ 7.97–7.95 (m, 2H), 7.58–7.55 (m, 1H), 7.49–7.45 (m, 2H), 3.64–3.46 (m, 1H), 3.27–3.24 (m, 3H), 3.10 (d, *J* = 12.3 Hz, 1H), 2.90 (dd, *J* = 12.4, 9.9 Hz, 1H), 2.71–2.69 (m, 1H), 2.04–2.02 (m, 1H), 1.84–1.56 (m, 3H). ^13^C-NMR (101 MHz, CDCl_3_): δ 202.4, 136.0, 133.1, 128.7, 128.3, 48.9, 46.3, 44.7, 28.0, 25.4. HRMS (+ESI) *m*/*z* calculated for [M + H]^+^ 190.1226, found 190.1230. 

*3-(4-Methylbenzoyl)piperidin-1-ium Chloride* (**2b**). Colorless oil. ^1^H-NMR (400 MHz, CDCl_3_): δ 7.87 (d, *J* = 8.2 Hz, 2H), 7.32–7.23 (m, 2H), 3.74 (br, 2H), 3.67–3.56 (m, 2H), 3.29 (d, *J* = 12.2 Hz, 1H), 3.16 (d, *J* = 12.7 Hz, 1H), 2.94 (dd, *J* = 12.4, 10.2 Hz, 1H), 2.80–2.64 (m, 1H), 2.41 (s, 3H), 2.10–1.97 (m, 1H), 1.82–1.75 (m, 1H), 1.74–1.62 (m, 1H), 1.48 (t, *J* = 7.3 Hz, 1H). ^13^C-NMR (101 MHz, CDCl_3_): δ 201.4, 144.1, 133.2, 129.5, 128.5, 48.2, 45.8, 43.7, 27.8, 24.6, 21.6. HRMS (+ESI) *m*/*z* calculatedfor [M + H]^+^ 204.1383, found 204.1387.

*3-(4-Fluorobenzoyl)piperidin-1-ium Chloride* (**2c**). Yellow oil. ^1^H-NMR (400 MHz, CDCl_3_): δ 8.00 (dd, *J* = 8.7, 5.5 Hz, 2H), 7.17–7.13 (m, 2H), 3.63–3.45 (m, 1H), 3.34 (br, 2H), 3.26 (d, *J* = 12.1 Hz, 1H), 3.12 (d, *J* = 11.8 Hz, 1H), 2.96–2.82 (m, 1H), 2.72 (t, *J* = 9.6 Hz, 1H), 2.10–2.05 (m, 1H), 1.86–1.62 (m, 3H). ^13^C-NMR (101 MHz, CDCl_3_): δ 200.5, 166.8 (d, *J* = 253.0 Hz), 132.2 (d, *J* = 3.0 Hz), 131.0 (d, *J* = 9.0 Hz), 115.9 (d, *J* = 22.0 Hz), 48.6, 46.1, 44.3, 27.9, 25.0. HRMS (+ESI) *m*/*z* calculatedfor [M + H]^+^ 208.1132, found 208.1136.

*3-(2-Methylbenzoyl)piperidin-1-ium Chloride* (**2d**). Colorless oil. ^1^H-NMR (400 MHz, CDCl_3_): δ 7.63 (d, *J* = 7.8 Hz, 1H), 7.40–7.36 (m, 1H), 7.32–7.24 (m, 2H), 5.03 (br, 2H), 3.64–3.59 (m, 1H), 3.42 (dd, *J* = 12.4, 2.8 Hz, 1H), 3.27 (dt, *J* = 12.4, 3.6 Hz, 1H), 3.05–2.93 (m, 1H), 2.87–2.71 (m, 1H), 2.45 (s, 3H), 2.11–1.99 (m, 1H), 1.84 (tt, *J* = 7.2, 3.6 Hz, 2H), 1.66–1.51 (m, 1H). ^13^C-NMR (101 MHz, CDCl_3_): δ 204.9, 138.2, 136.8, 132.0, 131.4, 128.1, 125.8, 46.9, 45.4, 45.2, 26.9, 23.7, 21.0. HRMS (+ESI) *m*/*z* calculated for [M + H]^+^ 204.1383, found 204.1387. 

*3-(2-Naphthoyl)piperidin-1-ium Chloride* (**2e**). Yellow oil. ^1^H-NMR (400 MHz, CDCl_3_): δ 8.55 (s, 1H), 8.09–7.97 (m, 2H), 7.95–7.85 (m, 2H), 7.64–7.55 (m, 2H), 4.29 (br, 2H), 3.94–3.82 (m, 1H), 3.44 (d, *J* = 11.7 Hz, 1H), 3.30–3.20 (m, 1H), 3.04 (dd, *J* = 12.3, 10.4 Hz, 1H), 2.90–2.75 (m, 1H), 2.18–2.09 (m, 1H), 1.95–1.84 (m, 2H), 1.80–1.73 (m, 1H). ^13^C-NMR (101 MHz, CDCl_3_): δ 201.3, 135.7, 132.8, 132.6, 130.1, 129.7, 128.7, 128.7, 127.8, 126.9, 124.0, 48.1, 45.7, 43.5, 27.7, 24.3. HRMS (+ESI) *m*/*z* calculated for [M + H]^+^ 240.1383, found 240.1387.

*3-Pentanoylpiperidin-1-ium Chloride* (**2f**). Yellow oil. ^1^H-NMR (400 MHz, CDCl_3_): δ 3.61–3.55 (m, 1H), 3.46 (t, *J* = 14.0 Hz, 2H), 3.14 (tt, *J* = 11.5, 3.4 Hz, 1H), 3.03–2.91 (m, 1H), 2.82 (td, *J* = 12.6, 3.3 Hz, 1H), 2.54–2.43 (m, 2H), 2.18 (d, *J* = 13.4 Hz, 1H), 2.07–2.04 (m, 1H), 2.00–1.85 (m, 1H), 1.63–1.40 (m, 4H), 1.36–1.30 (m, 2H), 0.92 (t, *J* = 7.3 Hz, 3H). ^13^C-NMR (101 MHz, CDCl_3_): δ 209.7, 45.3, 44.7, 43.9, 40.7, 25.9, 25.4, 22.3, 21.9, 13.8. HRMS (+ESI) *m*/*z* calculated for [M + H]^+^ 170.1539, found 170.1543.

*3-Isobutyrylpiperidin-1-ium Chloride* (**2g**). Yellow oil. ^1^H-NMR (400 MHz, CDCl_3_): δ 3.88 (br, 1H), 3.55–3.40 (m, 2H), 3.40–3.27 (m, 2H), 3.05–2.95 (m, 1H), 2.93–2.73 (m, 2H), 2.15–1.93 (m, 3H), 1.58–1.40 (m, 1H), 1.15–1.08 (m, 6H). ^13^C-NMR (101 MHz, CDCl_3_): δ 213.4, 44.9, 43.9, 43.3, 39.3, 25.8, 19.3, 18.6, 17.8. HRMS (+ESI) *m*/*z* calculated for [M + H]^+^ 156.1383, found 156.1386.

*3-(Thiophene-2-carbonyl)piperidin-1-ium Chloride* (**2h**). Yellow oil.^1^H-NMR (400 MHz, CDCl_3_): δ 7.82 (d, *J* = 3.8 Hz, 1H), 7.68 (d, *J* = 4.9 Hz, 1H), 7.19–7.14 (m, 1H), 3.55–3.44 (m, 1H), 3.33 (d, *J* = 12.0 Hz, 1H), 3.21–3.08 (m, 3H), 3.02–2.93 (m, 1H), 2.77 (t, *J* = 11.5 Hz, 1H), 2.10–2.07 (m, 1H), 1.91–1.67 (m, 3H). ^13^C-NMR (101 MHz, CDCl_3_): δ 194.7, 143.5, 134.1, 132.1, 128.3, 48.7, 46.0, 29.7, 28.0, 24.9. HRMS (+ESI) *m*/*z* calculated for [M + H]^+^ 196.0791, found 196.0794.

*3-Benzoyl-5-phenethylpiperidin-1-ium Chloride* (**2i**). Yellow oil. ^1^H-NMR (400 MHz, CDCl_3_): δ 8.03 (d, *J* = 7.3 Hz, 0.45H), 7.96 (d, *J* = 7.3 Hz, 0.92H), 7.70–7.40 (m, 4H), 7.28–7.02 (m, 5H), 4.29–4.15 (m, 1H), 4.03 (d, *J* = 4.2 Hz, 1H), 3.72–3.55 (m, 2H), 3.45–3.41 (m, 1H), 3.21–2.85 (m, 2H), 2.77–2.49 (m, 3H), 2.47–2.17 (m, 1H), 2.13–1.99 (m, 1H), 1.91–1.84 (m, 2H), 1.79–1.47 (m, 2H). ^13^C-NMR (101 MHz, CDCl_3_): δ 201.4, 199.0, 161.8, 159.2, 141.2, 140.7, 134.8, 134.3, 134.2, 133.9, 129.1, 129.0, 128.7, 128.7, 128.6, 128.5, 128.3, 128.2, 126.2, 48.7, 47.8, 45.4, 44.8, 37.2, 35.6, 33.9, 33.4, 33.1, 32.6, 31.1, 29.7, 29.7. HRMS (+ESI) *m*/*z* calculated for [M + H]^+^ 294.1852, found 294.1855.

*3-Benzoyl-5-methylpiperidin-1-ium Chloride* (**2j**). Colorless oil. ^1^H-NMR (400 MHz, CDCl_3_): δ 7.98 (d, *J* = 7.5 Hz, 1.5H), 7.93 (d, *J* = 7.5 Hz, 0.3H), 7.61–7.57 (m, 1H), 7.51–7.47 (m, 2H), 3.59–3.52 (m, 1H), 3.29 (t, *J* = 10.8 Hz, 1H), 3.09 (d, *J* = 12.8 Hz, 0.72H), 3.01 (d, *J* = 12.8 Hz, 0.24H), 2.74 (t, *J* = 11.7 Hz, 1H), 2.37 (dd, *J* = 12.9, 9.0 Hz, 0.18H), 2.25 (dd, *J* = 12.9, 9.0 Hz, 0.78H), 2.20–2.12 (m, 0.2H), 2.02 (d, *J* = 13.3 Hz, 0.91H), 1.77 (td, *J* = 11.2, 7.0 Hz, 0.94H), 1.69–1.60 (m, 0.31H), 1.39–1.22 (m, 2H), 0.91 (dd, *J* = 13.4, 6.6 Hz, 3H).^13^C-NMR (101 MHz, CDCl_3_): δ 201.8, 136.0, 133.2, 128.8, 128.3, 53.6, 53.0, 48.4, 47.4, 45.3, 40.4, 36.5, 34.8, 31.5, 29.3, 19.4, 18.8. HRMS (+ESI) *m*/*z* calculated for [M + H]^+^ 204.1383, found 204.1387.

*3-Benzoyl-5-phenylpiperidin-1-ium Chloride* (**2k**). Colorless oil. ^1^H-NMR (400 MHz, CDCl_3_): δ 8.02 (d, *J* = 7.4 Hz, 1.68H), 7.95 (d, *J* = 7.4 Hz, 0.32H), 7.61–7.57 (m, 1H), 7.51–7.47 (m, 2H), 7.37–7.19 (m, 5H), 4.43 (br, 2H), 4.06–3.90 (m, 1H), 3.62–3.46 (m, 1H), 3.44–3.41 (m, 0.78H), 3.35–3.33 (m, 0.28H), 3.25–3.12 (m, 1H), 3.05 (t, *J* = 12.0 Hz, 1H), 2.96–2.87 (m, 0.4H), 2.84 (t, *J* = 12.1 Hz, 0.76H), 2.31–2.28 (m, 1H), 2.03–1.85 (m, 1H). ^13^C-NMR (101 MHz, CDCl_3_): δ 200.5, 141.8, 141.8, 135.5, 135.4, 133.5, 133.4, 128.9, 128.8, 128.3, 128.4, 127.2, 127.1, 52.0, 51.6, 47.3, 46.5, 43.8, 41.7, 40.0, 38.6, 34.7, 33.1. HRMS (+ESI) *m*/*z* calculated for [M + H]^+^ 266.1539, found 266.1545.

*3-Benzoyl-5-(4-methoxyphenyl)piperidin-1-ium Chloride* (**2l**). Colorless oil. ^1^H-NMR (400 MHz, CDCl_3_): δ 8.00 (d, *J* = 7.3 Hz, 1.66H), 7.93 (d, *J* = 7.3 Hz, 0.38H), 7.61–7.57 (m, 1H), 7.54–7.44 (m, 2H), 7.18 (d, *J* = 8.6 Hz, 1.61H), 7.13 (d, *J* = 8.6 H, 0.39H), 6.88–6.84 (m, 2H), 3.80 (s, 2.3H), 3.79 (s, 0.57H), 3.72 (tt, *J* = 11.5, 3.4 Hz, 1H), 3.49 (d, *J* = 12.3 Hz, 0.24H), 3.38 (d, *J* = 12.3 Hz, 0.82H), 3.28–3.24 (m, 0.88H), 3.20–3.16 (m, 0.18H), 3.03 (dd, *J* = 13.6, 3.9 Hz, 0.26H), 2.95–2.87 (m, 1.52H), 2.79–2.65 (m, 2H), 2.62 (br, 2H), 2.35–2.32 (m, 0.31H), 2.26–2.17 (m, 0.81H), 2.01–1.84 (m, 1H). ^13^C-NMR (101 MHz, CDCl_3_): δ 203.9, 201.6, 158.4, 158.2, 136.0, 135.9, 135.8, 135.4, 133.2, 133.1, 128.8, 128.3, 128.1, 128.0, 114.0, 113.9, 55.3, 53.5, 53.1, 48.6, 47.2, 45.6, 42.4, 40.6, 38.5, 36.9, 35.1, 33.7. HRMS (+ESI) *m*/*z* calculated for [M + H]^+^ 296.1645, found 296.1647.

*3-Benzoyl-5-(4-fluorophenyl)piperidin-1-ium Chloride* (**2m**). Yellow oil. ^1^H-NMR (400 MHz, CDCl_3_): δ 8.04–7.97 (m, 1.39H), 7.97–7.90 (m, 0.49H), 7.62–7.57 (m, 1H), 7.52–7.47 (m, 2H), 7.23–7.16 (m, 2H), 7.03–6.96 (m, 2H), 3.76 (tt, *J* = 11.5, 3.6 Hz, 0.72H), 3.68–3.64 (m, 0.29H), 3.50 (d, *J* = 13.5 Hz, 0.27H), 3.41 (d, *J* = 12.4 Hz, 0.7H), 3.29–3.21 (m, 1H), 3.12–2.82 (m, 4H), 2.82–2.65 (m, 1H), 2.33 (d, *J* = 13.2 Hz, 0.28H), 2.26–2.18 (m, 0.81H), 2.17–2.08 (m, 0.24H), 2.02–1.84 (m, 0.75H). ^13^C-NMR (101 MHz, CDCl_3_): δ 203.6, 201.3, 161.7 (d, *J* = 243.0 Hz), 161.5 (d, *J* = 243.0 Hz), 139.3 (d, *J* = 3.0 Hz), 138.7 (d, *J* = 4.0 Hz), 135.8 (d, *J* = 8.0 Hz), 133.3 (d, *J* = 11.0 Hz), 128.8, 128.6, 128.5, 128.3, 115.4 (d, *J* = 21.0 Hz), 115.23 (d, *J* = 21.0 Hz),53.1, 52.8, 48.4, 47.1, 45.2, 42.3, 40.4, 38.5, 34.9, 33.6. HRMS (+ESI) *m*/*z* calculated for [M + H]^+^ 284.1445, found 284.1453.

*3-Benzoyl-5-(naphthalen-1-yl)piperidin-1-ium Chloride* (**2n**). Colorless oil. ^1^H-NMR (400 MHz, CDCl_3_): δ 8.23 (d, *J* = 8.5 Hz, 1H), 8.05 (d, *J* = 7.4 Hz, 1.58H), 7.99 (d, *J* = 7.4 Hz, 0.65H), 7.89 (d, *J* = 8.0 Hz, 0.69H), 7.84 (d, *J* = 8.0 Hz, 0.4H), 7.76 (d, *J* = 8.0 Hz, 0.62H), 7.73 (d, *J* = 8.0 Hz, 0.33H) 7.65–7.36 (m, 7H), 3.98–3.60 (m, 3H), 3.81–3.42 (m, 1.45H), 3.15 (dd, *J*=13.6, 4.0 Hz, 0.46H), 3.08–2.97 (m, 1H), 2.83 (t, *J* = 11.7 Hz, 1H), 2.57 (d, *J* = 13.5 Hz, 0.45H), 2.38 (d, *J* = 13.2 Hz, 0.77H), 2.27–2.16 (m,1H), 2.13– 2.00 (m, 2H). ^13^C-NMR (101 MHz, CDCl_3_): δ 203.6, 201.3, 162.9, 162.7, 160.4, 160.3, 139.3, 138.8, 138.7, 135.8, 135.8, 133.3, 133.2, 128.8, 128.6, 128.5, 128.3, 115.5, 115.4, 115.3, 115.2, 53.1, 52.8, 48.4, 47.1, 45.2, 42.3, 40.4, 38.5, 34.9, 33.6. HRMS (+ESI) *m*/*z* calculated for [M + H]^+^ 316.1696, found 316.1701.

*3-Benzoyl-5-(thiophen-2-yl)piperidin-1-ium Chloride* (**2o**). Yellow oil. ^1^H-NMR (400 MHz, CDCl_3_): δ 8.01 (d, *J* = 7.3 Hz, 1.67H), 7.93 (d, *J* = 7.3 Hz, 0.28H), 7.61–7.57 (m, 1H), 7.51–7.47 (m, 2H), 7.31–7.25 (m, 1H), 7.04 (d, *J* = 1.7 Hz, 1H), 7.00 (dd, *J* = 5.0, 0.8 Hz, 1H), 4.03 (br, 2H), 3.91 (tt, *J* = 11.8, 3.3 Hz, 1H), 3.59–3.53 (m, 0.17H), 3.47 (d, *J* = 12.2 Hz, 2H), 3.38–3.31 (m, 0.24H), 3.29–3.22 (m, 0.9H), 3.13–3.05 (m, 0.35H), 3.02–2.96 (m, 0.89H), 2.93–2.87 (m, 0.22H), 2.77–2.72 (m, 1H), 2.66 (d, *J* = 9.3 Hz, 1H), 2.40–2.33 (m, 1H), 2.26–2.15 (m, 0.29H), 1.89–1.80 (m, 1H). ^13^C-NMR (101 MHz, CDCl_3_): δ 203.1, 200.7, 147.0, 146.1, 135.7, 133.4, 133.3, 128.9, 128.8, 128.4, 126.8, 126.8, 123.5, 123.3, 123.3, 53.2, 52.7, 47.9, 44.6, 40.3, 37.8, 36.0, 34.7, 34.2, 29.7. HRMS (+ESI) *m*/*z* calculated for [M + H]^+^ 272.1104, found 272.1110.

*3-Benzoyl-5-(thiophen-3-yl)piperidin-1-ium Chloride* (**2p**). Yellow oil. ^1^H-NMR (400 MHz, CDCl_3_): δ 8.02–7.99 (m, 1.64H), 7.95–7.90 (m, 0.33H), 7.62–7.57 (m, 1H), 7.55–7.46 (m, 2H), 7.30–7.28 (m, 1H), 7.06–6.98 (m, 2H), 3.78 (tt, *J* = 11.6, 3.4 Hz, 0.81H), 3.71–3.62 (m, 0.23H), 3.46–3.36 (m, 2H), 3.28 (br, 2H), 3.22–3.01 (m, 2H), 3.02–2.83 (m, 1H), 2.79–2.60 (m, 1H), 2.41–2.32 (m, 1H), 2.26–2.13 (m, 0.31H), 1.92–1.82 (m, 0.89H). ^13^C-NMR (101 MHz, CDCl_3_) δ203.2, 200.3, 143.4, 142.5, 135.4, 133.6, 133.5, 128.9, 128.9, 128.5, 128.4, 126.7, 126.4, 126.1, 125.9, 120.4, 120.1, 51.1, 50.9, 46.9, 46.4, 43.4, 39.8, 34.7, 34.1, 33.1, 29.7. HRMS (+ESI) *m*/*z* calculated for [M + H]^+^ 272.1104, found 272.1110.

*5-Benzoyl-2-phenylpiperidin-1-ium Chloride* (**2q**). Yellow oil. ^1^H-NMR (400 MHz, CDCl_3_): δ 8.09–8.02 (m, 1.33H), 7.99–7.94 (m, 0.64H), 7.73–7.64 (m, 0.73H), 7.64–7.55 (m, 1.42H), 7.55–7.48 (m, 2.52H), 7.47–7.44 (m, 1.47H), 7.39–7.34 (m, 2H), 3.88–3.80 (m, 0.36H), 3.79–3.70 (m, 1.41H), 3.61–3.56 (m, 0.59H), 3.48–3.36 (m, 0.75H), 3.21 (dd, *J* = 13.9, 4.3 Hz, 0.38H), 3.04 (t, *J* = 11.5 Hz, 0.75H), 2.65 (br, 2H), 2.18–2.11 (m, 1H), 2.02–1.96 (m, 1H), 1.87–1.82 (m, 1H). ^13^C-NMR (101 MHz, CDCl_3_): δ 203.7, 201.9, 136.0, 133.2, 133.1, 132.1, 132.1, 132.0, 128.8, 128.6, 128.5, 128.4, 128.3, 127.6, 127.4, 126.8, 126.6, 61.7, 60.0, 49.5, 47.5, 44.1, 39.5, 33.4, 29.7, 29.3, 28.6. HRMS (+ESI) *m*/*z* calculated for [M + H]^+^ 266.1539, found 266.1545.

*5-(4-Methoxybenzoyl)-2-(4-methoxyphenyl)piperidin-1-ium Chloride* (**2r**). Yellow oil. ^1^H-NMR (400 MHz, CDCl_3_): δ 7.97 (d, *J* = 8.2 Hz, 0.28H), 7.87 (d, *J* = 8.2 Hz, 1.92H), 7.32–7.28 (m, 4H), 7.16 (d, *J* = 7.9 Hz, 2.24H), 4.50 (br, 2H), 3.96–3.86 (m, 1H), 3.78–3.75 (m, 0.17H), 3.67–3.51 (m, 1.87H), 3.48–3.45 (m, 0.19H), 3.24 (dd, *J* = 13.4, 3.7 Hz, 0.93H), 3.05 (t, *J* = 11.7 Hz, 0.14H), 2.44 (s, 3.09H), 2.40 (s, 0.19H), 2.34 (s, 2.84H), 2.30 (s, 0.2H), 2.25–2.07 (m, 2H), 1.92–1.84 (m, 2H). ^13^C-NMR (101 MHz, CDCl_3_): δ 203.1, 144.2, 138.8, 137.3, 133.1, 129.5, 129.3, 128.6, 126.6, 59.2, 47.0, 39.0, 28.7, 26.5, 21.7, 21.1.

*5-(4-(Trifluoromethyl)benzoyl)-2-(4-(trifluoromethyl)phenyl)piperidin-1-ium Chloride* (**2s**). Yellow oil. ^1^H-NMR (400 MHz, CDCl_3_): δ 8.03 (d, *J* = 8.1 Hz, 2H), 7.77 (d, *J* = 8.2 Hz, 2H), 7.59 (d, *J* = 8.1 Hz, 2H), 7.48 (d, *J* = 8.3 Hz, 2H), 3.87–3.71 (m, 1H), 3.60 (dt, *J* = 13.1, 2.1 Hz, 1H), 3.51–3.47 (m, 1H), 3.20 (dd, *J* = 13.2, 3.9 Hz, 1H), 2.41–2.24 (m, 1H), 2.14–1.95 (m, 1H), 1.89 (br, 2H), 1.86–1.79 (m, 2H). ^13^C-NMR (101 MHz, CDCl_3_): δ 202.6, 148.3, 139.2, 134.1 (q, *J* = 32.6 Hz), 129.3 (q, *J* = 32.3 Hz), 125.8 (q, *J* = 3.7 Hz), 125.4 (q, *J* = 3.8 Hz), 124.2(q, *J* = 272.7 Hz ), 123.6 (q, *J* = 273.7 Hz), 60.3, 48.1, 40.7, 30.1, 26.2. HRMS (+ESI) *m*/*z* calculated for [M + H]^+^ 402.1287, found 402.1309.

*3-(Ethoxycarbonyl)piperidin-1-ium Chloride* (**2t**) [23]. Colorless oil. ^1^H-NMR (400 MHz, CDCl_3_): δ 4.13 (q,*J* = 7.1 Hz, 2H), 3.16 (dd, *J* = 12.4, 3.6 Hz, 1H), 2.93 (dt, *J* = 12.2, 3.9 Hz, 1H), 2.81 (dd, *J* = 12.4, 9.3 Hz, 1H), 2.72–2.57 (m, 1H), 2.47–2.40 (m, 1H), 2.06–1.94 (m, 2H), 1.75 (br, 2H), 1.71–1.59 (m, 2H), 1.53–1.38 (m, 1H), 1.25 (t, *J* = 7.1 Hz, 3H). ^13^C-NMR (101 MHz, CDCl_3_) δ 174.3, 60.2, 48.5, 46.3, 42.4, 27.3, 25.40, 14.2.

*3-(Ethoxycarbonyl)-5-methylpiperidin-1-ium Chloride* (**2u**). Yellow oil. ^1^H-NMR (400 MHz, CDCl_3_): δ 4.34–4.11 (m, 2H), 3.77–3.65 (m, 1H), 3.60–3.54 (m, 1H), 3.47–3.32 (m, 1H), 3.21–3.10 (m, 1H), 3.04–2.92 (m, 0.67H), 2.84 (t, *J* = 12.6 Hz, 0.35H), 2.75–2.69 (m, 0.64H), 2.65 (d, *J* = 9.3 Hz, 0.24H), 2.26–2.14 (m, 0.70H), 2.11–2.06 (m, 1.34H), 1.64–1.53 (m, 0.7H), 1.51–1.47 (m, 0.29H), 1.33–1.25 (m, 3H), 1.08 (d, *J* = 7.8 Hz, 1.77H), 1.01 (d, *J* = 7.8 Hz). ^13^C-NMR (101 MHz, CDCl_3_): δ 172.2, 171.3, 61.9, 61.2, 49.6, 49.5, 44.3, 44.0, 38.7, 36.2, 34.3, 32.2, 25.5, 18.6, 18.3, 14.1, 14.1. HRMS (+ESI) *m*/*z* calculatedfor [M + H]^+^ 172.1332, found 172.1339.

*3-(Ethoxycarbonyl)-5-phenylpiperidin-1-ium Chloride* (**2v**). Colorless oil. ^1^H-NMR (400 MHz, CDCl_3_): δ 7.37–7.31 (m, 2H), 7.28–7.21 (m, 3H), 4.34–4.13 (m, 2H), 3.51 (d, *J* = 13.2 Hz, 0.74H), 3.43 (d, *J* = 13.2 Hz, 0.31H), 3.25–3.20 (m, 1H), 2.91–2.65 (m, 4H), 2.43 (d, *J* = 13.5 Hz, 0.86H), 2.33 (d, *J* = 13.5 Hz, 0.44H), 2.22 (br, 2H), 1.99–1.92 (ddd, *J* = 13.6, 9.6, 4.4 Hz, 0.86H), 1.86–1.77 (q, *J* = 12.6 Hz, 0.37H ), 1.34–1.28 (m, 3H). ^13^C-NMR (101 MHz, CDCl_3_): δ 174.5, 143.7, 128.6, 128.5, 127.1, 126.8, 126.5, 60.6, 53.0, 47.0, 34.4, 32.9, 29.7, 14.3, 14.2, 12.0. HRMS (+ESI) *m*/*z* calculated for [M + H]^+^ 234.1489, found 234.1493.

*3-(Ethoxycarbonyl)-5-(4-methoxyphenyl)piperidin-1-ium Chloride* (**2w**). Colorless oil. ^1^H-NMR (400 MHz, CDCl_3_): δ 7.18–7.12 (m, 2H), 6.91–6.84 (m, 2H), 4.36–4.09 (m, 2H), 3.81 (s, 3H), 3.50 (d, *J* = 13.2 Hz, 0.77H), 3.44 (d, *J* = 13.2 Hz, 0.32H), 3.21 (t, *J* = 13.5 Hz, 1H), 2.90–2.75 (m, 2H), 2.73 (br, 2H), 2.71–2.59 (m, 2H), 2.40 (d, *J* = 13.1 Hz, 0.83H), 2.31 (d, *J* = 13.1 Hz, 0.36H), 1.95–1.87 (ddd, *J* = 13.6, 9.6, 4.4 Hz, 0.79H), 1.81–1.72 (q, *J* = 12.6 Hz, 0.34H), 1.34–1.25 (m, 3H). ^13^C-NMR (101 MHz, CDCl_3_): δ 174.4, 173.3, 158.4, 158.2, 135.7, 134.9, 128.0, 114.0, 113.9, 60.7, 60.6, 55.3, 53.0, 52.4, 47.4, 46.8, 39.6, 39.2, 34.5, 33.1, 29.7, 14.3, 14.2. HRMS (+ESI) *m*/*z* calculated for [M + H]^+^ 264.1594, found 264.1598.

*3-(Ethoxycarbonyl)-5-(4-fluorophenyl)piperidin-1-ium Chloride* (**2x**). Colorless oil. ^1^H-NMR (400 MHz, CDCl_3_): δ 7.22–7.16 (m, 2H), 7.05–6.97 (m, 2H), 4.31–4.11 (m, 2H), 3.49 (d, *J* = 13.3 Hz, 0.7H), 3.43–3.35 (m, 0.33H), 3.16 (d, *J* = 11.7 Hz, 1H), 2.89–2.81 (m, 1H), 2.76–2.59 (m, 3H), 2.44–2.35 (m, 0.72H), 2.31–2.26 (m, 0.34H), 2.00 (br, 2H), 1.92–1.85 (ddd, *J* = 13.6, 9.6, 4.4 Hz*,* 0.84H), 1.81–1.72 (q, *J* = 12.6 Hz, 0.40H), 1.34–1.26 (m, 3H). ^13^C-NMR (101 MHz, CDCl_3_): δ 174.1, 172.8, 161.7 (d, *J* = 244.0 Hz), 161.6 (d, *J* = 243.0 Hz), 139.0 (d, *J* = 3.0 Hz), 138.0 (d, *J* = 3.0 Hz), 128.5 (d, *J* = 8.0 Hz), 128.4 (d, *J* = 8.0 Hz), 115.5 (d, *J* = 22.0 Hz), 115.4 (d, *J* = 21.0 Hz), 60.8, 52.7, 51.7, 46.8, 46.6, 41.9, 41.1, 39.4, 39.0, 34.2, 32.9, 29.7, 14.3, 14.2. HRMS (+ESI) *m*/*z* calculated for [M + H]^+^ 252.1394, found 252.1399.

*3-(Benzyloxy)-5-(ethoxycarbonyl)piperidin-1-ium Chloride* (**2y**). Yellow oil. ^1^H-NMR (400 MHz, CDCl_3_): δ 7.55–7.30 (m, 5H), 4.91 (br, 2H), 4.70–4.62 (m, 0.62H), 4.64–4.54 (m, 1.62H), 4.24–4.04 (m, 2H), 3.94–3.80 (m, 1H), 3.62–3.33 (m, 2H), 3.21–3.02 (m, 1H), 3.03–2.78 (m, 2H), 2.45–2.41 (m, 0.56H), 2.34–2.31 (m, 0.52H), 1.92–1.79 (m, 0.56H), 1.79–1.66 (m, 0.49H), 1.28–1.23 (m, 3H). ^13^C-NMR (101 MHz, CDCl_3_): δ 172.3, 171.8, 137.6, 128.5, 128.5, 127.9, 127.9, 127.8, 127.6, 71.1, 70.7, 70.6, 68.5, 61.4, 61.2, 47.6, 46.8, 45.4, 44.9, 37.5, 35.6, 31.6, 30.2, 14.1, 14.1. HRMS (+ESI) *m*/*z* calculated for [M + H]^+^ 264.1594, found 264.1598.

*1,2,3,4-Tetrahydroquinoxaline* (**4a**) [29]. Yellow oil. ^1^H-NMR (400 MHz, CDCl_3_): δ 6.63–6.61 (m, 2H), 6.56–6.47 (m, 2H), 3.45 (s, 4H). ^13^C-NMR (101 MHz, CDCl_3_) δ 133.7, 118.8, 114.7, 41.4.

*2-Phenyl-1,2,3,4-tetrahydroquinoxaline* (**4b**) [40]. White solid. ^1^H-NMR (400 MHz, CDCl_3_): δ 7.50–7.39 (m, 4H), 7.38–7.34 (m, 1H), 6.70–6.67 (m, 2H), 6.64 –6.61 (m, 2H), 4.52 (dd, *J* = 8.2, 3.1 Hz, 1H), 3.92 (br, 2H), 3.50 (dd, *J* = 11.0, 3.1 Hz, 1H), 3.37 (dd, *J* = 11.0, 8.2 Hz, 1H). ^13^C-NMR (101 MHz, CDCl_3_) δ 141.9, 134.2, 132.9, 128.7, 127.9, 127.0, 118.9, 118.8, 114.7, 114.5, 54.8, 49.2.

*2-(4-Chlorophenyl)-4-methyl-3,4-dihydroquinazoline* (**4c**). Yellow solid. ^1^H-NMR (400 MHz, CDCl_3_): δ 7.79 (d, *J* = 8.4 Hz, 2H), 7.43 (d, *J* = 8.4 Hz, 2H), 7.27–7.23 (m, 1H), 7.19 (d, *J* = 7.6 Hz, 1H), 7.13–7.09 (m, 1H), 7.04 (d, *J* = 7.4 Hz, 1H), 4.91 (q, *J* = 6.5 Hz, 1H), 1.54 (d, *J* = 6.5 Hz, 3H). ^13^C-NMR (101 MHz, CDCl_3_): δ 152.9, 141.0, 136.7, 133.9, 128.8, 128.1, 128.0, 125.8, 125.1, 125.0, 123.3, 49.3, 25.6.

*4-(4-Chlorophenyl)-1-methyl-1,2-dihydrophthalazine* (**4d**). Yellow solid. ^1^H-NMR (400 MHz, CDCl_3_): δ 7.59 (d, *J* = 8.4 Hz, 2H), 7.48–7.42 (m, 3H), 7.34–7.27 (m, 1H), 7.25–7.22 (m, 2H), 6.07 (br, 1H), 4.41 (q, *J* = 6.4 Hz, 1H), 1.55 (d, *J* = 6.5 Hz, 3H). ^13^C-NMR (101 MHz, CDCl_3_): δ 148.1, 137.2, 135.0, 134.1, 130.7, 129.7, 128.6, 127.3, 125.3, 125.0, 123.7, 49.9, 18.1.

*1,2,3,4-Tetrahydroquinoline* (**4e**) [41]. Colorless oil. ^1^H-NMR (400 MHz, CDCl_3_): δ 7.08–7.03 (m, 2H), 6.72–6.68 (m, 1H), 6.55 (dd, *J* = 7.8, 0.6 Hz, 1H), 3.84 (br, 1H), 3.42–3.32 (m, 2H), 2.85 (t, *J* = 6.4 Hz, 2H), 2.08–1.97 (m, 2H). ^13^C-NMR (101 MHz, CDCl_3_) δ 144.9, 129.6, 126.8, 121.5, 117.0, 114.3, 42.1, 27.1, 22.3.

*1,2,3,4-Tetrahydroisoquinolin-2-ium Chloride* (**4f**) [41]. Colorless oil. ^1^H-NMR (400 MHz, CDCl_3_): δ 7.27–7.18 (m, 2H), 7.18–7.13 (m, 1H), 7.13–7.07 (m, 1H), 5.47 (br, 2H), 4.27 (s, 2H), 3.38 (t, *J* = 6.1 Hz, 2H), 3.08 (t, *J* = 6.1 Hz, 2H). ^13^C-NMR (101 MHz, CDCl_3_) δ 136.1, 134.9, 129.4, 126.3, 126.0, 125.8, 48.4, 44.0, 29.3.

*1,2,3,4-Tetrahydro-1,5-naphthyridine* (**4g**) [42]. Yellow oil. ^1^H-NMR (400 MHz, CDCl_3_): δ 7.88 (d, *J* = 4.5 Hz, 1H), 6.91 (dd, *J* = 8.0, 4.7 Hz, 1H), 6.76 (d, *J* = 8.0 Hz, 1H), 3.39–3.24 (m, 2H), 2.96 (t, *J* = 6.5 Hz, 2H), 2.09–2.02 (m, 2H). ^13^C-NMR (101 MHz, CDCl_3_) δ 151.2, 137.9, 124.4, 121.9, 120.2, 41.5, 30.3, 21.8.

*1,2,3,4-Tetrahydro-1,10-phenanthroline* (**4h**) [43]. Yellow oil. ^1^H-NMR (400 MHz, CDCl_3_): δ 8.71 (d, *J* = 3.1 Hz, 1H), 8.03 (d, *J* = 8.2 Hz, 1H), 7.32 (dd, *J* = 8.2, 4.2 Hz, 1H), 7.18 (d, *J* = 8.2 Hz, 1H), 7.00 (d, *J* = 8.2 Hz, 1H), 5.95 (s, 1H), 3.60–3.52 (m, 2H), 2.95 (t, *J* = 6.3 Hz, 2H), 2.17–2.04 (m, 2H). ^13^C-NMR (101 MHz, CDCl_3_) δ 147.0, 140.7, 137.5, 135.9, 129.1, 127.4, 120.6, 116.6, 113.1, 41.3, 27.1, 21.8.

*Ethyl 1,4,5,6-tetrahydropyridine-3-carboxylate* (**intermediate C**) [12]. Yellow oil. ^1^H-NMR (400 MHz, CDCl_3_): δ 7.48 (d, *J* = 6.1 Hz, 1H), 4.45 (s, 1H), 4.14 (q, *J* = 7.1 Hz, 2H), 3.27–3.15 (m, 2H), 2.34 (t, *J* = 6.2 Hz, 2H), 1.82 (dt, *J* = 11.9, 6.1 Hz, 2H), 1.26 (t, *J* = 7.1 Hz, 3H).

### 3.2. The Stereochemical Assignment of Disubstituted Piperidines

**2v** (41.2 mg, 0.15 mmol) and l-bromopropane (30.1 mg, 0.23 mmol) were refluxed in absolute ethanol (2 mL) with sodium bicarbonate (47.7 mg, 0.45 mol) for 18 h. The mixture was filtered through a pad of Celite. The inorganic salts were washed with several portions of fresh ethanol. The combined filtrates were evaporated in vacuo and the residue was purified by flash column chromatography to obtain compounds **5** (20.2 mg, 0.07 mmol, 46.5%) and **6** (5.4 mg, 0.02 mmol, 13.3%).

*trans-Ethyl 1-butyl-5-phenylpiperidine-3-carboxylate* (**5**) [39]. Yellow oil. ^1^H-NMR (400 MHz, CDCl_3_) δ 7.30 (d, *J* = 4.8 Hz, 4H), 7.23–7.17 (m, 1H), 4.23–4.15 (m, 2H), 3.29 (d, *J* = 9.6 Hz, 1H), 3.16–3.09 (m, 1H), 2.87 (dd, *J* = 11.4, 4.0 Hz 1H), 2.78–2.68 (m, 1H), 2.46–2.11 (m, 5H), 1.69–1.62 (m, 1H), 1.57–1.38 (m, 2H), 1.38 (m, 5H), 0.91 (t, *J* = 7.3 Hz, 3H).

*cis-Ethyl 1-butyl-5-phenylpiperidine-3-carboxylate* (**6**) [39]. Yellow oil. ^1^H-NMR (400 MHz, CDCl_3_) δ 7.40–7.30 (m, 2H), 7.24–7.19 (m, 3H),4.16 (q, *J* = 7.6 Hz, 1H), 3.26 (dt, *J* = 11.8 Hz, 1.6 Hz 1H), 3.04 (dt, *J* = 11.8 Hz, 1.6 Hz 1H), 2.95–2.82 (m, 1H), 2.82–2.68 (m, 1H), 2.42 (t, *J* = 7.2 Hz, 2H), 2.24 (d, *J* = 12.6 Hz, 1H), 2.05 (t, *J* = 11.3 Hz, 1H), 1.97 (t, *J* = 10.8 Hz, 1H), 1.64 (q, *J* = 12.3 Hz, 1H), 1.57–1.44 (m, 2H), 1.39–1.31 (m, 2H), 1.26 (t, *J* = 7.4 Hz, 3H), 0.94 (t, *J* = 7.3 Hz, 1H).

## 4. Conclusions

In conclusion, we have developed a HMPA-catalyzed metal-free transfer hydrogenation method for the reduction of pyridines. The functional group tolerance of this method provides an easy access method to various piperidines with ester or ketone groups at the C-3 position. The suitability of the method for the reduction of other N-heteroarenes has also been demonstrated. Efforts to extend the application of chiral HMPA derivatives in metal free pyridine reduction with HSiCl_3_ are currently underway.

## Figures and Tables

**Table 1:**
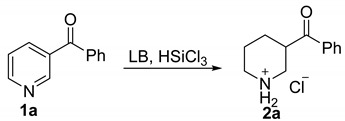
Optimization of reaction conditions ^a^.

Entry	Solvent	LB (Equiv.)	Temp (°C)	Yield (%) ^b^
1	DCM	HMPA (0.2)	0	49.0
2	DCM	DMF (0.2)	0	trace
3	DCM	POPh_3_ (0.2)	0	37.0
4	DCM	HMPA (0.2)	−10	38.0
5	DCM	HMPA (0.2)	25	82.0
6	THF	HMPA (0.2)	25	64.0
7	CHCl_3_	HMPA (0.2)	25	76.0
8	CCl_4_	HMPA (0.2)	25	trace
9	DCE	HMPA (0.2)	25	77.0
10	toluene	HMPA (0.2)	25	trace
11	MeCN	HMPA (0.2)	25	trace
12 ^c^	DCM	HMPA (0.2)	25	60.0
13 ^d^	DCM	HMPA (0.2)	25	81.0
14 ^e^	DCM	HMPA (0.2)	25	86.0
15 ^f^	DCM	HMPA (0.2)	25	96.0
16 ^f^	DCM	HMPA (0.1)	25	82.0
17 ^f^	DCM	HMPA (0.05)	25	54.0

^a^ Unless otherwise specified, all reactions were performed with pyridines (0.1 mmol), HMPA and HSiCl_3_ (0.6 mmol) in solvent (1 mL) for 12 h. ^b^ Isolated yield. ^c^ HSiCl_3_ (0.4 mmol). ^d^ HSiCl_3_ (0.5 mmol). ^e^ HSiCl_3_ (0.8 mmol). ^f^ Reaction time is 24 h. hexamethylphosphoramide (HMPA).

## Supplementary Materials

The following are available online: General experimental procedures, substrates, product characterization data, ^1^H- and ^13^C-NMR spectra substrates and NMR spectra.
