# Supplementary material for: HMPA-Catalyzed Transfer Hydrogenation of 3-Carbonyl Pyridines and Other N-Heteroarenes with Trichlorosilane
Source: Molecules. 2019 Jan 22;24(3):401. doi: 10.3390/molecules24030401 (PMC6384841; doi:10.3390/molecules24030401)

Supporting Information

HMPA Catalysed Cascade Transfer Hydrogenations of 3-carbonyl Pyridines and other N-heteroarenes with Trichlorosilane

Yun Fu[1], Jian Sun[1]*

[1] Chengdu Institute of Biology, Chinese Academy of Sciences, Chengdu, China, 610041. University of Chinese Academy of Sciences, Beijing, China ,100049. fy860401@163.com(Y.F); sunjian@cib.ac.cn(J.S).

* Correspondence: sunjian@cib.ac.cn(J.S); Tel.: +86-28-85222753

Table of Contents

General Information....1
Preparation of Starting Materials....1
References....8
NMR Spectra....10

General Information

All solvents used in the reactions were distilled from appropriate drying agents prior to use. Reactions were monitored by thin layer chromatography using silica gel HSGF254 plates. Flash chromatography was performed using silica gel HG/T2354-92. ^{1}H - and ^{13}C NMR (400 and 100 MHz, respectively) spectras were recorded in $CDCl_3$. ^{1}H NMR chemical shifts are reported in ppm (δ) relative to tetramethylsilane (TMS) with the solvent resonance employed as the internal standard ($CDCl_3$, δ 7.26 ppm). Datas were reported as follows: chemical shift, multiplicity (s = singlet, d = doublet, t = triplet, q = quartet, m = multiplet, br = broad, dd = double doublet), coupling constants (Hz) and integration. ^{13}C NMR chemical shifts were reported in ppm from tetramethylsilane (TMS) with the solvent resonance as the internal standard ($CDCl_3$, δ 77.0 ppm). ESIMS spectras were recorded on BioTOF Q.

Preparation of Starting Materials

The substrates **1a**[1], **1b**[2], **1c**[2], **1u**[3], **1y**[4], **3b**[5], **3c**[6], **3d**[7], **1q**, **1r**, **1s**[8] were prepared according to the previous reported procedures. The substrates **1t**, **3a**, **3b**, **3e**, **3f**, **3g**, **3h** were purchased from commercial sources.

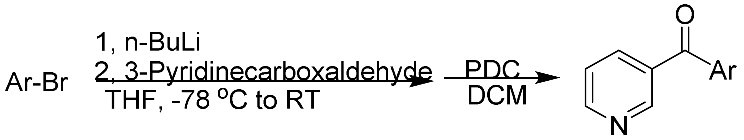

Scheme 1 Synthesis of **1d**, **1e** and **1h**.

n-Butyllithium (2.5 M solution in hexane, 0.88 mL, 2.2 mmol) was added dropwise to a solution of aryl bromide (2.0 mmol) in THF (10 mL) at -78 °C for 30 min. And then 3-pyridinecarboxaldehyde (261 mg, 2.1 mmol) was added to the mixture at -78 °C. The obtained mixture was stirred at room temperature for 1 h and monitored with TLC. Saturated NH_4Cl solution was added to quench the reaction. The mixture was extracted with EA. The combined organic layers were dried over Na_2SO_4, concentrated under vacuum to get the crude product. The crude was dissolved in DCM (20 mL), and then PDC (1.02 g, 2.7 mmol)

was added. The reaction mixture was stirred for 3 hours at room temperature and monitored with TLC. The mixture was filtered through Celite and washed with DCM. The combined organic layers were washed with brine, dried over Na_2SO_4, concentrated under vacuum to afford the crude product. The desired products were obtained by column chromatography on silica gel.

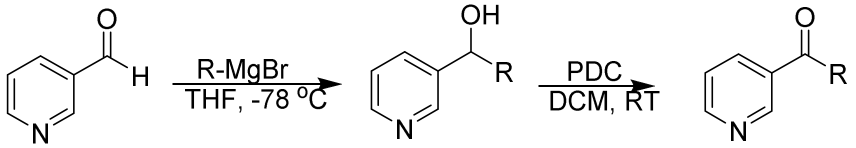

Scheme 2 Synthesis of **1f**, **1g**.

R-MgBr (1 M solution in THF, 0.88 mL, 2.2 mmol) was added dropwise to a solution of 3-pyridinecarboxaldehyde (201.1 mg, 2 mmol) in THF (10 mL) at -78 oC for 30 min. The obtained mixture was stirred at room temperature for 1 h and monitored with TLC. Saturated NH4Cl solution was added to quench the reaction. The mixture was extracted with EA. The combined organic layers were dried over Na2SO4, concentrated under vacuum to get the crude product. The crude product was dissolved in DCM, and PDC (1.02 g, 2.7 mmol) was added. The reaction was stirred at room temperature for 3 h and monitored with TLC. The mixture was filtered through a bed of Celite and later washed with DCM. The combined organic layers were washed with brine, dried over Na_2SO_4, concentrated under vacuum to afford the crude product. The desired products were obtained by column chromatography on silica gel.

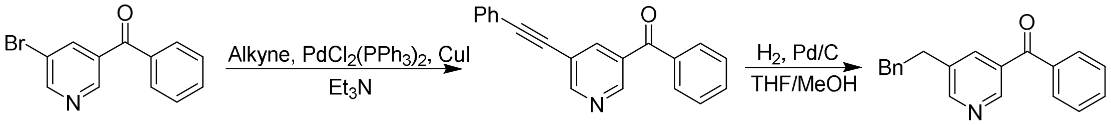

Scheme 3 Synthesis of **1i**

$PdCl_2(PPh_3)_2$ (148 mg, 0.21 mmol) and CuI (120.6 mg, 0.63 mmol) were added to a solution of (5-bromopyridin-3-yl)(phenyl)methanone (1.1 g, 4.22 mmol) in Et_3N (10 mL) under N_2, and the resulting mixture was stirred for 30 min. Then ethynylbenzene (516 mg, 5.1 mmol) was added dropwise and the reaction mixture was stirred overnight at room temperature, followed by filtration over Celite and evaporation under vacuum. Purification by column chromatography on silica gel afforded the desired product.

To the crude product in a mixture of THF (12 mL) and MeOH (12 mL) was added 10% Pd on carbon (80 mg), and the atmosphere was changed to H_2 (2.5 bar). The resulting mixture was stirred in a Parr hydrogenation apparatus overnight at room temperature, followed by filtration over Celite and evaporation under vacuum. Purification by column chromatography on silica gel afforded the desired product.

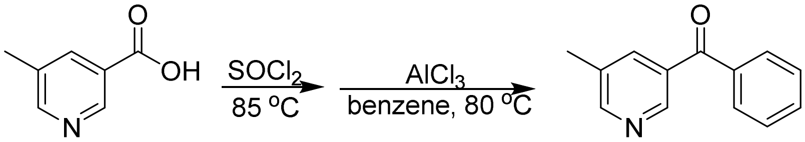

Scheme 4 Synthesis of **1j**

A suspension of 5-methylnicotic acid (2 mmol) in thionyl chloride (10 mL) was heated at 85 °C for 90 min. The excess of thionyl chloride was evaporated under vacuum, and the crude

residue was treated with benzene (15mL) and portion wise while stirring with anhydrous aluminum chloride (6 mmol) at 0 °C. The mixture was refluxed for 6 h, and then cooled, poured on ice containing 37% HCl (5 mL), and extracted with chloroform. The organic solution was washed with 1 N NaOH (5 mL) and brine (5 mL), and then dried and evaporated under vacuum. The residue was passed through a silica gel column chromatography to give the desired product.

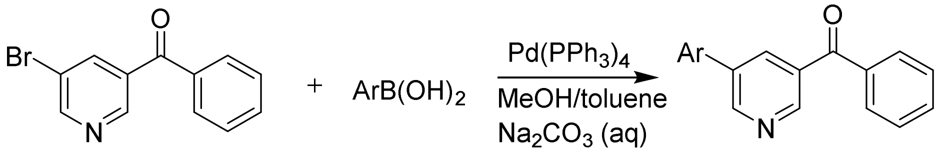

Scheme 5 Synthesis of **1k**, **1l**, **1m**, **1n**, **1o** and **1p**

Pd(PPh_3)$_4$ (17.3 mg, 0.015 mmol) was added to a solution of 3-benzoy-5-bromo pyridine (130.1 mg, 0.5 mmol) and aryl boronic acid (0.6 mmol) in MeOH (0.2 mL), toluene (0.8 mL), and 2 M Na_2CO_3 (0.2mL) under N_2. The mixture was heated to 75 °C for 2 h, and then cooled to room temperature and concentrated under reduced pressure. Water was added to the residue and the aq. phase was extracted with DCM (3 × 5 mL). The combined organic layers were washed with brine, dried over Na_2SO_4, and evaporated to obtain the crude product. Purification by column chromatography on silica gel afforded the desired product.

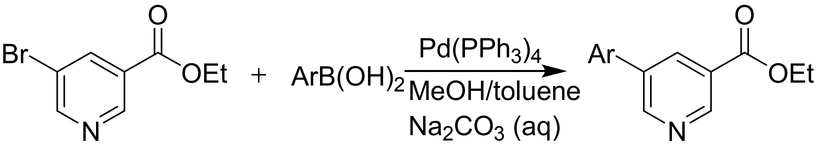

Scheme 6 Synthesis of **1v**, **1w**, **1x**

Pd(PPh_3)$_4$ (17.3 mg, 0.015 mmol) was added to a solution of 5-bromonicotinic acid ethyl ester(115.1 mg, 0.5 mmol) and aryl boronic acid (0.6 mmol) in MeOH (0.2 mL), toluene (0.8 mL), and 2 M Na_2CO_3 (0.2mL) under N_2. The mixture was heated to 75 °C for 2 h, then cooled to room temperature and concentrated under reduced pressure. Water was added to the residue and the aq. phase was extracted with DCM (3 × 5 mL). The combined organic layers were washed with brine, dried over Na_2SO_4, and evaporated to obtain the crude product. Purification by column chromatography on silica gel afforded the desired product.

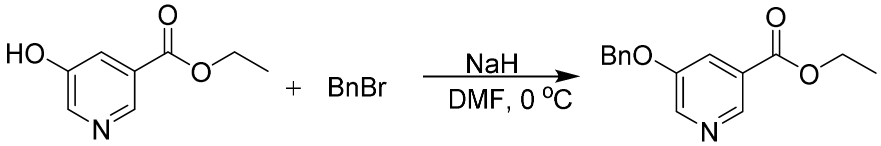

Scheme 7 Synthesis of **1y**

In an oven dried 25 ml flask, ethyl 5-hydroxynicotinate (5 mmol, 835 mg) was dissolved in 5 mL DMF under nitrogen atmosphere. This solution was transferred via cannula to another flask containing DMF (2 mL) solution of sodium hydride (55% in mineral oil, pre-washed with dry hexane) (10 mmol, 436 mg) and stirred under ice-cold condition for 30 minutes. To this solution, benzyl bromide (5 mmol, 593.7 µl) was slowly added under ice-cold condition and then the reaction mixture was allowed to warm to room temperature and stirred for 12 h. Upon completion, the reaction mixture was poured into ice-cold water and extracted with dichloromethane. The combined organic layers were dried over Na2SO4 and evaporated under reduced pressure. The crude reaction mixture was purified by column

chromatography.

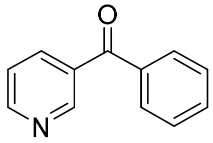

Phenyl(pyridin-3-yl)methanone(1a)[1]. Yellow oil. Yield: 78%. **^{1}H NMR** (400 MHz, $CDCl_3$): δ 9.00 (d, *J* = 1.6 Hz, 1H), 8.81 (dd, *J* = 4.9, 1.7 Hz, 1H), 8.13 - 8.11 (m, 1H), 7.86 - 7.78 (m, 2H), 7.64 - 7.62 (m, 1H), 7.52 - 7.50 (m, 2H), 7.46 - 7.44 (m, 1H).

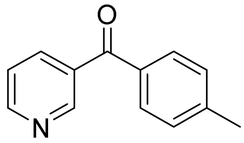

Pyridin-3-yl(p-tolyl)methanone(1b)[2]. Yellow oil. Yield: 58%. **^{1}H NMR** (400 MHz, $CDCl_3$): δ 8.99 (d, *J* = 1.2 Hz, 1H), 8.87 - 8.76 (m, 1H), 8.11 - 8.09 (m, 1H), 7.74 (d, *J* = 8.1 Hz, 2H), 7.45 (dd, *J* = 7.8, 4.7 Hz, 1H), 7.32 (d, *J* = 7.9 Hz, 2H), 2.46 (s, 3H).

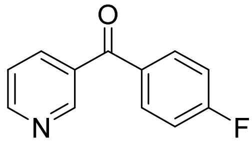

(4-fluorophenyl)(pyridin-3-yl)methanone (1c)[2]. Yellow solid. Yield: 46%. **^{1}H NMR** (400 MHz, $CDCl_3$): δ 8.99 (d, *J* = 1.7 Hz, 1H), 8.84 (dd, *J* = 4.8, 1.7 Hz, 1H), 8.12 - 8.10 (m, 1H), 7.89 - 7.87 (m, 2H), 7.49 (dd, *J* = 7.9, 4.9 Hz, 1H), 7.25 - 7.20 (m, 2H).

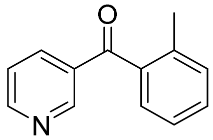

Pyridin-3-yl(o-tolyl)methanone(1d)[9]. White solid. Yield: 68%. **^{1}H NMR** (400 MHz, $CDCl_3$): δ 8.96 (d, *J* = 1.7 Hz, 1H), 8.82 (dd, *J* = 4.8, 1.2 Hz, 1H), 8.15 (d, *J* = 7.9 Hz, 1H), 7.49 - 7.43 (m, 2H), 7.35 (d, *J* = 5.6 Hz, 2H), 7.32 - 7.28 (m, 1H), 2.39 (s, 3H).

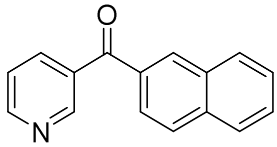

Naphthalen-2-yl(pyridin-3-yl)methanone (1e). White solid. Yield: 78%. **^{1}H NMR** (400 MHz, $CDCl_3$): δ 9.10 (d, *J* = 1.6 Hz, 1H), 8.88 (dd, *J* = 4.9, 1.7 Hz, 1H), 8.30 (s, 1H), 8.23 - 8.20 (m, 1H), 8.03 - 7.95 (m, 4H), 7.70 - 7.59 (m, 1H), 7.64 - 7.55 (m, 1H), 7.54 - 7.51 (m, 1H). **^{13}C NMR** (101 MHz, $CDCl_3$): δ 196.3, 153.4, 151.6, 137.4, 135.0, 134.0, 133.9, 132.3, 130.8, 128.7, 128.6, 127.8, 126.8, 125.4, 124.3, 123.5.

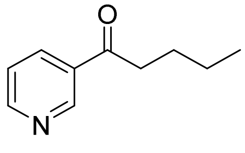

1-(pyridin-3-yl)pentan-1-one (1f)[10]. Yellow oil. Yield: 58%. **^{1}H NMR** (400 MHz, $CDCl_3$): δ 9.18 (d, *J* = 1.7 Hz, 1H), 8.78 (dd, *J* = 4.8, 1.6 Hz, 1H), 8.25– 8.23 (m, 1H), 7.44 (dd, *J* = 7.9, 4.8 Hz,

1H), 3.00 (t, *J* = 7.4 Hz, 2H), 1.77 - 1.75 (m, 2H), 1.50 - 1.36 (m, 2H), 0.97 (t, *J* = 7.3 Hz, 3H).

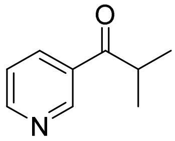

2-methyl-1-(pyridin-3-yl)propan-1-one(1g). Yellow oil. Yield: 43%. **¹H NMR** (400 MHz, $CDCl_3$): δ 9.16 (dd, *J* = 2.2, 0.6 Hz, 1H), 8.76 (dd, *J* = 4.8, 1.7 Hz, 1H), 8.27 - 8.17 (m, 1H), 7.42–7.40 (m, 1H), 3.54 - 3.50 (m, 1H), 1.24 (d, *J* = 6.9 Hz, 6H). **^{13}C NMR** (101 MHz, $CDCl_3$): δ 203.15, 153.2, 149.7, 135.7, 131.4, 123.7, 35.9, 18.8.

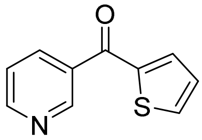

Pyridin-3-yl(thiophen-2-yl)methanone(1h)[11]. Yellow solid. Yield: 56%. **¹H NMR** (400 MHz, $CDCl_3$): δ 9.11 (d, *J* = 1.5 Hz, 1H), 8.85 (dd, *J* = 4.9, 1.7 Hz, 1H), 8.18 - 8.16 (m, 1H), 7.82 (dd, *J* = 4.9, 1.1 Hz, 1H), 7.69 (dd, *J* = 3.8, 1.1 Hz, 1H), 7.49 - 7.47 (m, 1H), 7.23 (dd, *J* = 4.9, 3.9 Hz, 1H).

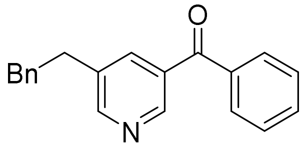

(5-phenethylpyridin-3-yl)(phenyl)methanone(1i). Yellow solid.Yield: 78%. **¹H NMR** (400 MHz, $CDCl_3$): δ 8.84 (d, *J* = 1.6 Hz, 1H), 8.63 (d, *J* = 2.0 Hz, 1H), 7.87 - 7.85 (m, 1H), 7.77 (d, *J* = 7.3 Hz, 2H), 7.67 - 7.65 (m, 1H), 7.54 - 7.51 (m, 2H), 7.31 - 7.25 (m, 3H), 7.17 (d, *J* = 7.1 Hz, 2H), 3.20 - 2.93 (m, 4H). **^{13}C NMR** (101 MHz, $CDCl_3$): δ 195.0, 153.1, 148.7, 140.3, 137.0, 136.8, 136.7, 133.1, 132.8, 130.0, 128.6, 128.6, 128.5, 126.4, 37.3, 34.7.

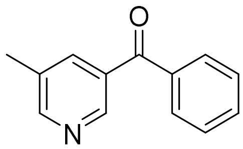

(5-methylpyridin-3-yl)(phenyl)methanone(1j). Yellow oil. Yield: 73%. **¹H NMR** (400 MHz, $CDCl_3$): δ 8.80 (d, *J* = 1.8 Hz, 1H), 8.67 (d, *J* = 1.7 Hz, 1H), 7.96 - 7.94 (m, 1H), 7.84 - 7.81 (m, 2H), 7.70 - 7.61 (m, 1H), 7.54 - 7.52 (m, 2H), 2.46 (s, 3H). **^{13}C NMR** (101 MHz, $CDCl_3$): δ 195.2, 153.4, 148.2, 137.4, 136.9, 133.2, 133.1, 132.8, 130.0, 128.6, 18.4.

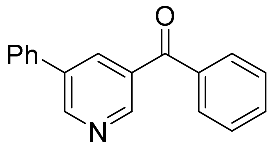

Phenyl(5-phenylpyridin-3-yl)methanone(1k). White solid. Yield: 88%. **¹H NMR** (400 MHz, $CDCl_3$): δ 9.07 (d, *J* = 2.0 Hz, 1H), 8.98 (d, *J* = 2.0 Hz, 1H), 8.33 - 8.32 (m, 1H), 7.95 - 7.86 (m, 2H), 7.74 - 7.62 (m, 3H), 7.56 - 7.54 (m, 4H), 7.48 - 7.46 (m, 1H). **^{13}C NMR** (101 MHz, $CDCl_3$): δ 194.9, 151.3, 149.5, 136.8, 136.8, 136.6, 135.4, 135.3, 133.3, 133.2, 130.1 129.3, 128.7, 127.3.

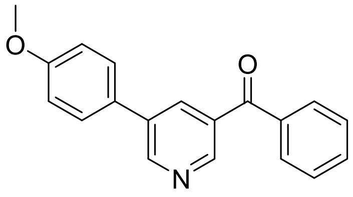

(5-(4-methoxyphenyl)pyridin-3-yl)(phenyl)methanone(1l). White solid. Yield: 72%. **^{1}H NMR** (400 MHz, $CDCl_3$): δ 9.03 (d, *J* = 2.4 Hz, 1H), 8.92 (d, *J* = 2.0 Hz, 1H), 8.29 - 8.28 (m, 1H), 7.95 - 7.85 (m, 2H), 7.68 - 7.52 (m, 5H), 7.08 - 7.05 (m, 2H), 3.90 (s, 3H). **^{13}C NMR** (101 MHz, $CDCl_3$): δ 195.1, 160.2, 150.9, 148.9, 136.8, 136.2, 134.8, 133.2, 133.1, 130.1, 129.1, 128.7, 128.4, 114.8, 55.4.

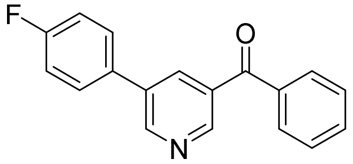

(5-(4-fluorophenyl)pyridin-3-yl)(phenyl)methanone(1m). White solid. Yield: 78%. **^{1}H NMR** (400 MHz, $CDCl_3$): δ 9.03 (d, *J* = 2.0 Hz, 1H), 8.96 (d, *J* = 2.0 Hz, 1H), 8.30 - 8.29 (m, 1H), 7.93 - 7.86 (m, 2H), 7.72 - 7.57 (m, 5H), 7.23 - 7.20 (m, 2H). **^{13}C NMR** (101 MHz, $CDCl_3$): δ 194.8, 163.2 (d, *J* = 249.5 Hz), 151.0, 149.5, 136.7, 135.7, 135.1, 133.3, 133.2, 132.9 (d, *J* = 3.0 Hz), 130.1, 129.0 (d, *J* = 8.1 Hz), 128.7, 116.3 (d, *J* = 20.2 Hz).

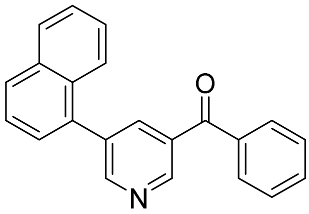

(5-(naphthalen-1-yl)pyridin-3-yl)(phenyl)methanone(1n). White solid. Yield: 63%.**^{1}H NMR** (400 MHz, $CDCl_3$): δ 9.09 (d, *J* = 1.6 Hz, 1H), 8.99 (d, *J* = 2.0 Hz, 1H), 8.28 - 8.27 (m, 1H), 7.98 - 7.67 (m, 5H), 7.67 - 7.52 (m, 7H). **^{13}C NMR** (101 MHz, $CDCl_3$): δ 194.9, 153.6, 149.7, 138.3, 136.8, 136.3, 135.2, 133.8, 133.3, 132.9, 131.3, 130.1, 129.1, 128.7, 128.7, 127.7, 126.9, 126.3, 125.4, 124.9.

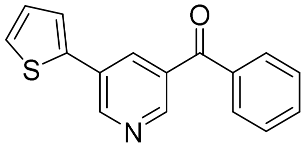

Phenyl(5-(thiophen-2-yl)pyridin-3-yl)methanone(1o). Yellow solid. Yield: 84%. **^{1}H NMR** (400 MHz, $CDCl_3$): δ 9.08 (d, *J* = 2.4 Hz, 1H), 8.90 (d, *J* = 2.0 Hz, 1H), 8.32 - 8.31 (m, 1H), 7.92 - 7.85 (m, 2H), 7.72 - 7.47 (m, 6H). **^{13}C NMR** (101 MHz, $CDCl_3$): δ 194.9, 150.5, 149.2, 137.7, 136.8, 134.4, 133.3, 133.3, 131.5, 130.1, 128.7, 127.4, 125.8, 122.4.

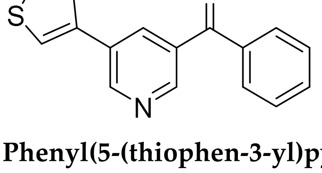

Phenyl(5-(thiophen-3-yl)pyridin-3-yl)methanone(1p). Yellow solid. Yield: 87%. **^{1}H NMR** (400 MHz, $CDCl_3$): δ 9.09 (d, *J* = 2.4 Hz, 1H), 8.88 (d, *J* = 2.0, Hz, 1H), 8.32 - 8.31 (m, 1H), 7.92 - 7.86 (m, 2H), 7.72 - 7.65 (m, 1H), 7.61 - 7.53 (m, 2H), 7.48 - 7.45 (m, 2H), 7.18 (s, 1H). **^{13}C NMR** (101 MHz, $CDCl_3$): δ 194.6, 149.8, 149.3, 139.2, 136.7, 133.7, 133.3, 133.2, 130.5, 130.1, 128.7,

128.5, 126.9, 125.1.

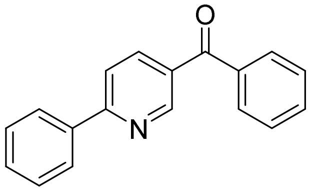

Phenyl(6-phenylpyridin-3-yl)methanone(1q)[8]. White solid. Yield: 56%. [1]**H NMR** (400 MHz, $CDCl_3$): δ 9.10 (dd, *J* = 2.2, 0.7 Hz, 1H), 8.25 (dd, *J* = 8.3, 2.3 Hz, 1H), 8.15 - 8.08 (m, 2H), 7.95 - 7.84 (m, 3H), 7.71 - 7.65 (m, 1H), 7.60 - 7.47 (m, 5H).

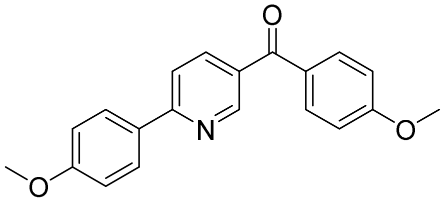

(4-methoxyphenyl)(6-(4-methoxyphenyl)pyridin-3-yl)methanone(1r). White solid. Yield: 69%. [1]**H NMR** (400 MHz, $CDCl_3$): δ 9.06 (d, *J* = 1.9 Hz, 1H), 8.20 (dd, *J* = 8.3, 2.2 Hz, 1H), 8.02 (d, *J* = 8.2 Hz, 2H), 7.87 (d, *J* = 8.3 Hz, 1H), 7.80 (d, *J* = 8.2 Hz, 2H), 7.35 (d, *J* = 8.0 Hz, 4H), 2.49 (s, H), 2.46 (s, 3H). [13]**C NMR** (101 MHz, $CDCl_3$): δ 194.4, 160.2, 151.1, 143.9, 140.2, 138.1, 135.5, 134.5, 131.3, 130.2, 129.7, 129.3, 127.2, 119.6, 21.7, 21.4.

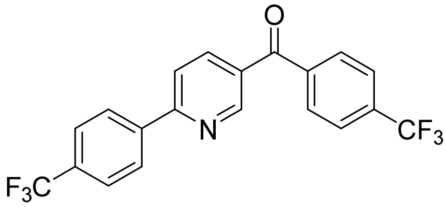

(4-(trifluoromethyl)phenyl)(6-(4-(trifluoromethyl)phenyl)pyridin-3-yl)methanone(1s). White solid. Yield: 60%. [1]**H NMR** (400 MHz, $CDCl_3$): δ 9.12 (d, *J* = 2.0 Hz, 1H), 8.34 – 8.20 (m, 3H), 7.98 (dd, *J* = 8.0, 5.4 Hz, 3H), 7.85 - 7.80 (m, 4H). [13]**C NMR** (101 MHz, $CDCl_3$): δ 193.4, 159.2, 151.3, 141.2, 139.8, 138.4, 134.5 (q, *J* = 32.8 Hz), 131.9 (q, *J* = 32.6 Hz), 131.2, 130.1, 127.7, 125.9 (q, *J* = 3.8 Hz), 125.8 (q, *J* = 3.7 Hz), 124.0 (q, *J* = 273.7 Hz), 123.5 (q, *J* = 273.7 Hz), 120.4.

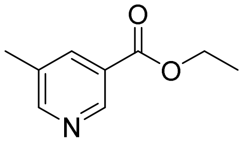

Ethyl 5-methylnicotinate(1u)[3]. Yellow oil. Yield: 89%. [1]**H NMR** (400 MHz, $CDCl_3$): δ 9.03 (d, *J* = 1.6 Hz, 1H), 8.60 (d, *J* = 1.6 Hz, 1H), 8.12-8.10 (m, 1H), 4.41 (q, *J* = 7.1 Hz, 2H), 2.41 (d, *J* = 0.6 Hz, 3H), 1.42 (t, *J* = 7.1 Hz, 3H).

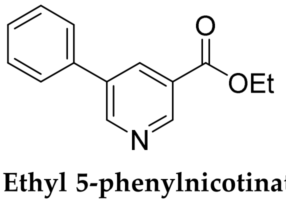

Ethyl 5-phenylnicotinate(1v)[12]**.** White solid.Yield: 87%. [1]**H NMR** (400 MHz, $CDCl_3$): δ 9.22 (d, *J* = 1.8 Hz, 1H), 9.02 (d, *J* = 2.2 Hz, 1H), 8.52 - 8.50 (m, 1H), 7.65 (dd, *J* = 5.3, 3.4 Hz, 2H), 7.56 – 7.50 (m, 2H), 7.50 – 7.43 (m, 1H), 4.47 (q, *J* = 7.1 Hz, 2H), 1.46 (t, *J* = 7.1 Hz, 3H).

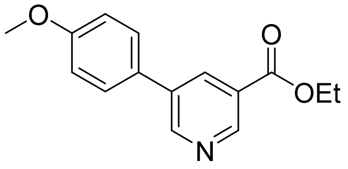

Ethyl 5-(4-methoxyphenyl)nicotinate(1w). White solid, Yield: 80%. **^{1}H NMR** (400 MHz, $CDCl_3$): δ 9.16 (d, *J* = 1.8 Hz, 1H), 8.98 (d, *J* = 2.2 Hz, 1H), 8.46– 8.45 (m, 1H), 7.63 – 7.56 (m, 2H), 7.12 – 7.01 (m, 2H), 4.46 (q, *J* = 7.1 Hz, 2H), 1.45 (t, *J* = 7.1 Hz, 3H). **^{13}C NMR** (101 MHz, $CDCl_3$): δ 165.4, 160.1, 151.3, 148.7, 136.1, 134.7, 129.1, 128.3, 126.3, 114.7, 61.5, 55.4, 14.3.

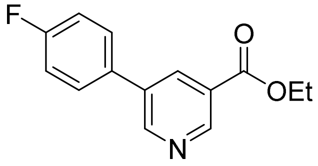

Ethyl 5-(4-fluorophenyl)nicotinate(1x)[4]. White solid.Yield: 85%. **^{1}H NMR** (400 MHz, $CDCl_3$): δ 9.20 (s, 1H), 8.96 (s, 1H), 8.44 (s, 1H), 7.60 (dd, *J* = 7.9, 5.4 Hz, 2H), 7.22 - 7.18 (m, 2H), 4.46 (q, *J* = 7.0 Hz, 2H), 1.45 (t, *J* = 7.1 Hz, 3H).

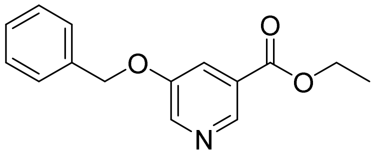

Ethyl 5-(benzyloxy)nicotinate(1y). Yellow oil. Yield: 67%. **^{1}H NMR** (400 MHz, $CDCl_3$): δ 8.87 (s, 1H), 8.57 (s, 1H), 7.88 (dd, *J* = 2.7, 1.5 Hz, 1H), 7.51 – 7.34 (m, 5H), 5.17 (s, 2H), 4.43 (q, *J* = 7.1 Hz, 2H), 1.43 (t, *J* = 7.1 Hz, 3H). **^{13}C NMR** (101 MHz, $CDCl_3$): δ 165.3, 143.2, 142.7, 135.6, 128.8, 128.5, 128.5, 127.6, 127.0, 121.3, 70.6, 61.6, 14.3.

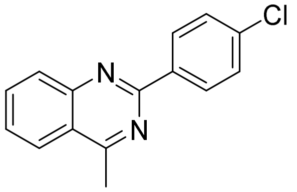

2-(4-chlorophenyl)-4-methylquinazoline(3c)[6]. White solid. Yield: 65%. ^{1}H NMR (400 MHz, $CDCl_3$): δ 8.68 – 8.54 (m, 2H), 8.17 – 8.00 (m, 2H), 7.92 – 7.88 (m, 1H), 7.65 – 7.60 (m, 1H), 7.56 – 7.43 (m, 2H), 3.04 (s, 3H).

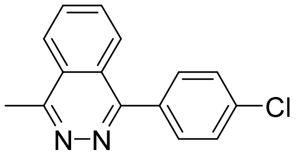

1-(4-chlorophenyl)-4-methylphthalazine(3d)[7]**.** Yellow solid. Yield: 48%. ^{1}H NMR (400 MHz, $CDCl_3$): δ 8.19 (d, *J* = 8.2 Hz, 1H), 8.04 (d, *J* = 8.2 Hz, 1H), 7.99 – 7.92 (m, 1H), 7.92 – 7.85 (m, 1H), 7.74 – 7.69 (m, 2H), 7.60 – 7.53 (m, 2H), 3.09 (s, 3H).

References

1. De Martino, G.; La Regina, G.; Di Pasquali, A.; Ragno, R.; Bergamini, A.; Ciaprini, C.; Sinistro, A.; Maga, G.; Crespan, E.; Artico, M.; Silvestri, R., Novel 1-[2-(Diarylmethoxy)ethyl]-2-methyl-5-nitroimidazoles as HIV-1 Non-Nucleoside Reverse

Transcriptase Inhibitors. A Structure–Activity Relationship Investigation. *J. Med. Chem.* **2005,** *48* (13), 4378-4388.
2. Letso, R. R.; Bauer, A. J.; Lunn, M. R.; Yang, W. S.; Stockwell, B. R., Small Molecule Screen Reveals Regulation of Survival Motor Neuron Protein Abundance by Ras Proteins. *ACS Chem. Biol.* **2013,** *8* (5), 914-922.
3. Palacios, F.; Herrán, E.; Alonso, C.; Rubiales, G.; Lecea, B.; Ayerbe, M.; Cossío, F. P., Reaction of N-Vinylic Phosphazenes with α,β-Unsaturated Aldehydes. Azatriene-Mediated Synthesis of Dihydropyridines and Pyridines Derived from β-Amino Acids. *J. Org. Chem.* **2006,** *71* (16), 6020-6030.
4. Kort, M. E.; Atkinson, R. N.; Thomas, J. B.; Drizin, I.; Johnson, M. S.; Secrest, M. A.; Gregg, R. J.; Scanio, M. J. C.; Shi, L.; Hakeem, A. H.; Matulenko, M. A.; Chapman, M. L.; Krambis, M. J.; Liu, D.; Shieh, C.-C.; Zhang, X.; Simler, G.; Mikusa, J. P.; Zhong, C.; Joshi, S.; Honore, P.; Roeloffs, R.; Werness, S.; Antonio, B.; Marsh, K. C.; Faltynek, C. R.; Krafte, D. S.; Jarvis, M. F.; Marron, B. E., Subtype-selective Nav1.8 sodium channel blockers: Identification of potent, orally active nicotinamide derivatives. *Bioorg. Med. Chem. Lett.* **2010,** *20* (22), 6812-6815.
5. Gopalaiah, K.; Saini, A.; Chandrudu, S. N.; Rao, D. C.; Yadav, H.; Kumar, B., Copper-catalyzed aerobic oxidative coupling of o-phenylenediamines with 2-aryl/heteroarylethylamines: direct access to construct quinoxalines. *Org. Biomol. Chem* **2017,** *15* (10), 2259-2268.
6. Baeckvall, J. E.; Nordberg, R. E.; Nystroem, J. E.; Hoegberg, T.; Ulff, B., Synthesis of 3-aryl-3-pyridylallylamines related to zimelidine via palladium-catalyzed amination. *J. Org. Chem.* **1981,** *46* (17), 3479-3483.
7. Tang, W.; Sun, Y.; Lijin, X.; Wang, T.; Qinghua, F.; Lam, K.-H.; Chan, A. S. C., Highly efficient and enantioselective hydrogenation of quinolines and pyridines with Ir-Difluorphos catalyst. *Org. Biomol. Chem.* **2010,** *8* (15), 3464-3471.
8. Abdel-Khalik, M. M.; Elnagdi, M. H., Enaminones in organic synthesis: A novel synthesis of 1, 3, 5-trisubstituted benzene derivatives and of 2-substituted-5-aroylpyridines. *Synth. Commun.* **2002,** *32* (2), 159-164.
9. Lu, B.; Wang, Q.; Zhao, M.; Xie, X.; Zhang, Z., Ruthenium-Catalyzed Enantioselective Hydrogenation of Ferrocenyl Ketones: A Synthetic Method for Chiral Ferrocenyl Alcohols. *J. Org. Chem.* **2015,** *80* (19), 9563-9569.
10. Mei, Z.-W.; Omote, T.; Mansour, M.; Kawafuchi, H.; Takaguchi, Y.; Jutand, A.; Tsuboi, S.; Inokuchi, T., A high performance oxidation method for secondary alcohols by inductive activation of TEMPO in combination with pyridine–bromine complexes. *Tetrahedron* **2008,** *64* (47), 10761-10766.
11. Benischke, A. D.; Le Corre, G.; Knochel, P., Preparation of Polyfunctional Organozinc Halides by an InX3- and LiCl-Catalyzed Zinc Insertion to Aryl and Heteroaryl Iodides and Bromides. *Chem.–Eur. J.* **2017,** *23* (4), 778-782.
12. Zeng, J.; Liu, K. M.; Duan, X. F., Selective Co/Ti Cooperatively Catalyzed Biaryl Couplings of Aryl Halides with Aryl Metal Reagents. *Org. Lett.* **2013,** *15* (20), 5342-5345.

NMR Spectra

The NMR spectra of 1a

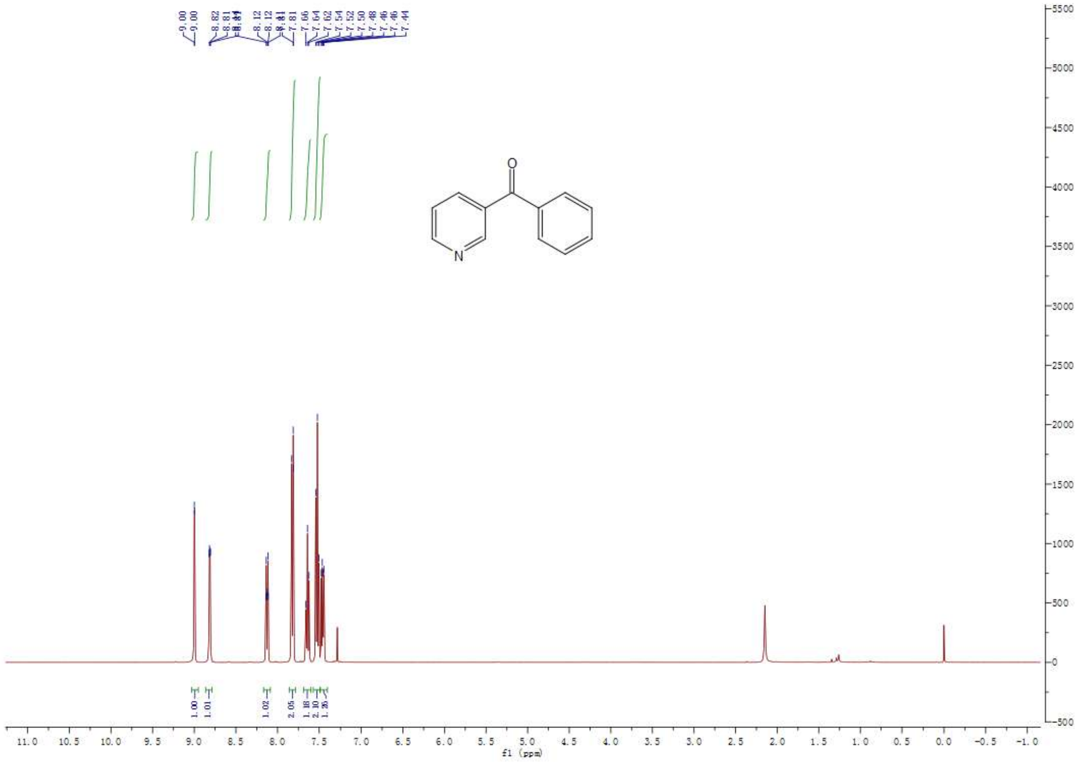

The NMR spectra of 1b

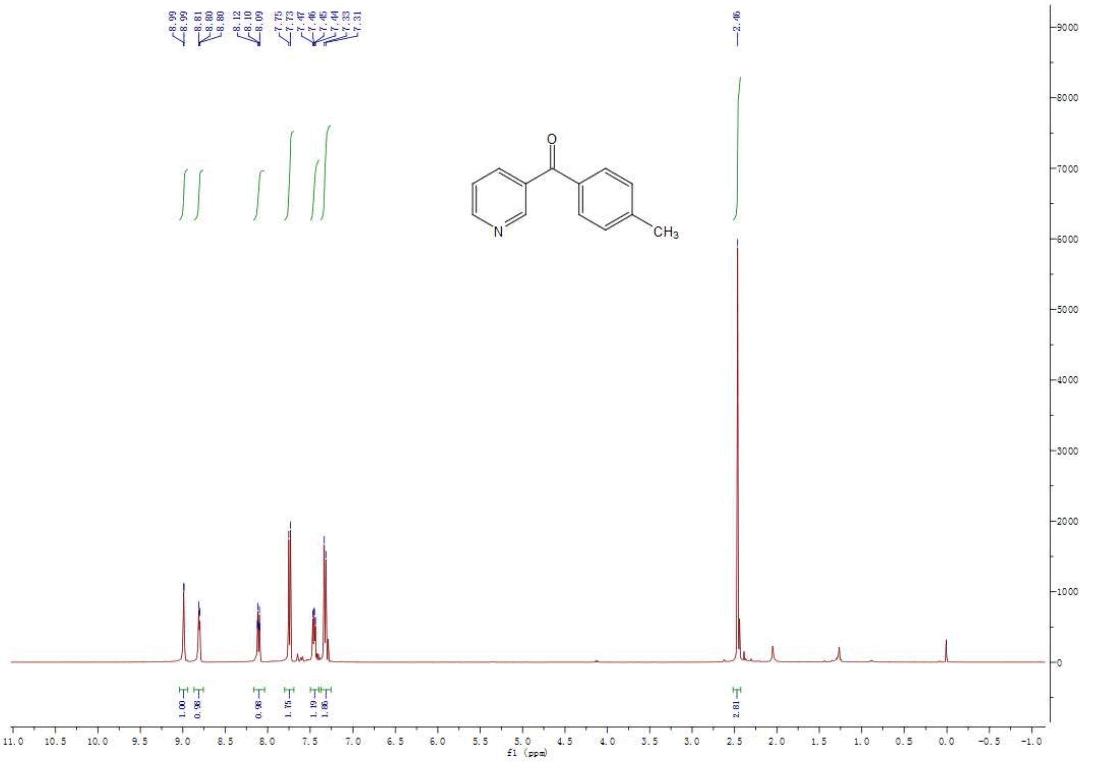

The NMR spectra of 1c

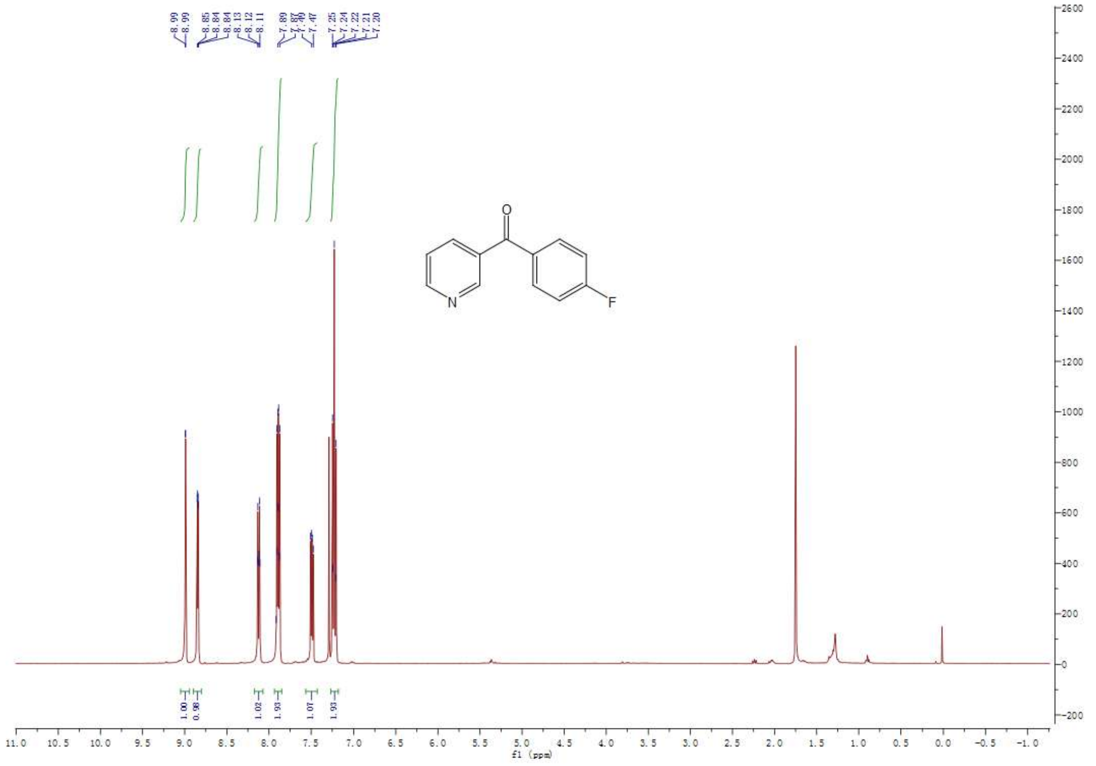

The NMR spectra of 1d

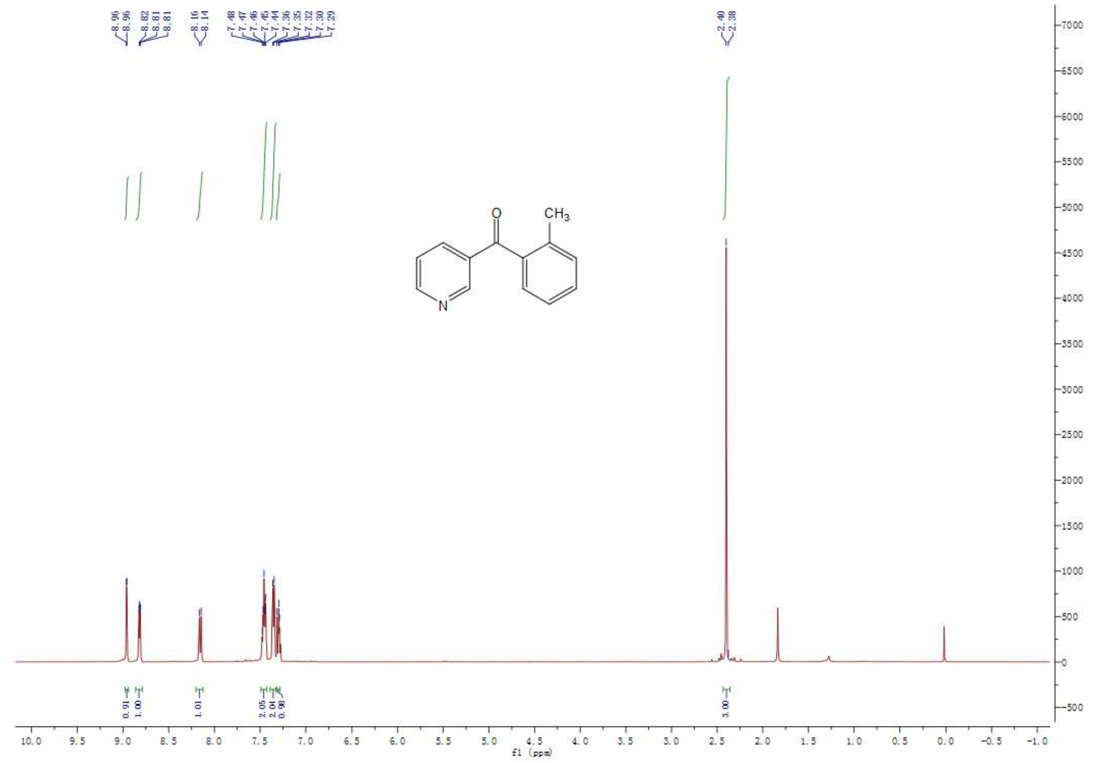

The NMR spectra of 1e

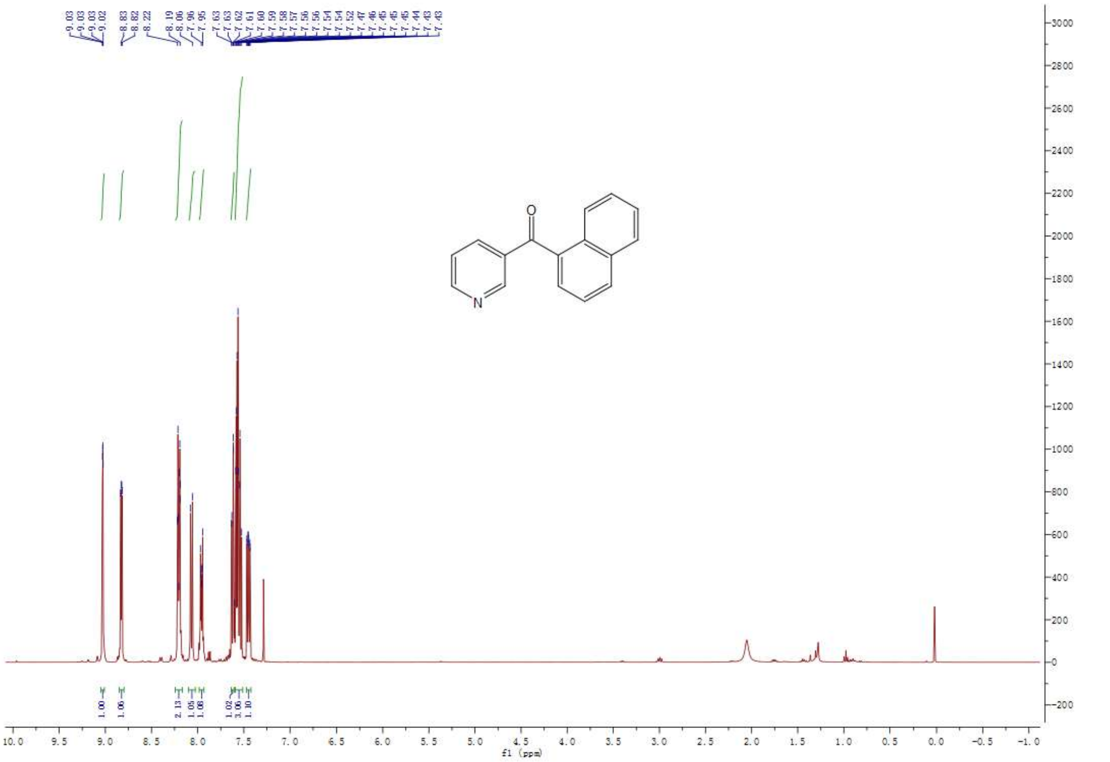

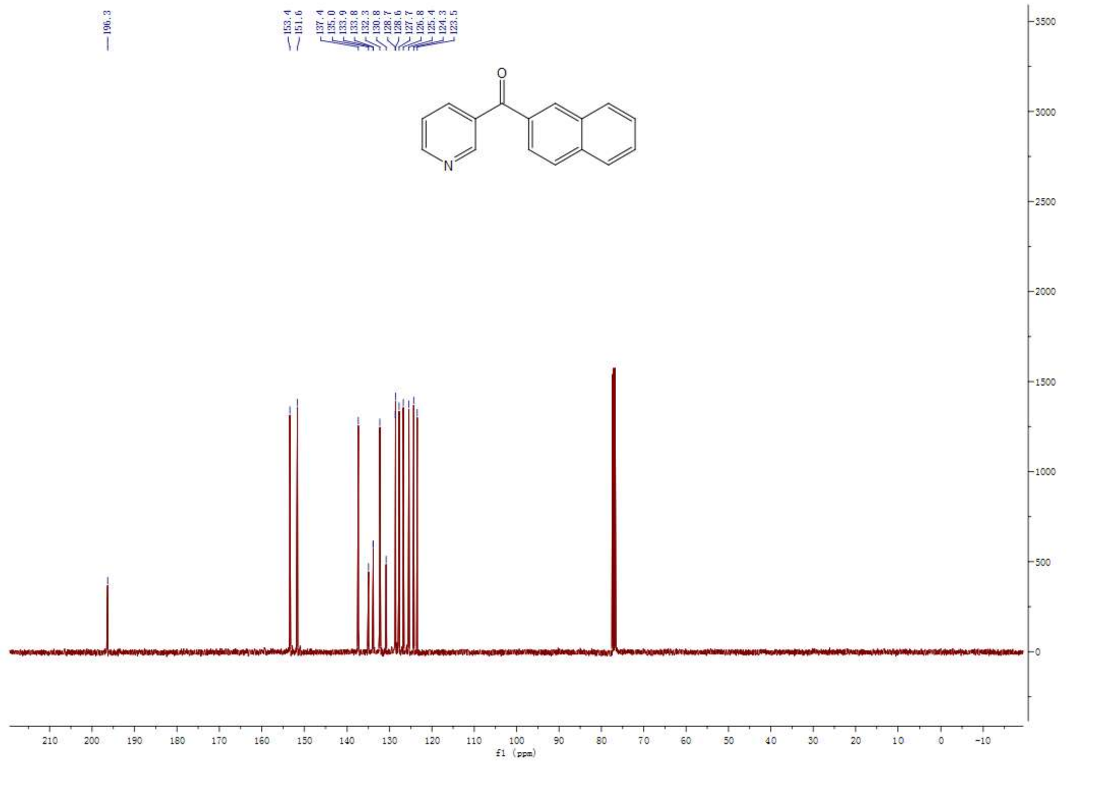

The NMR spectra of 1f

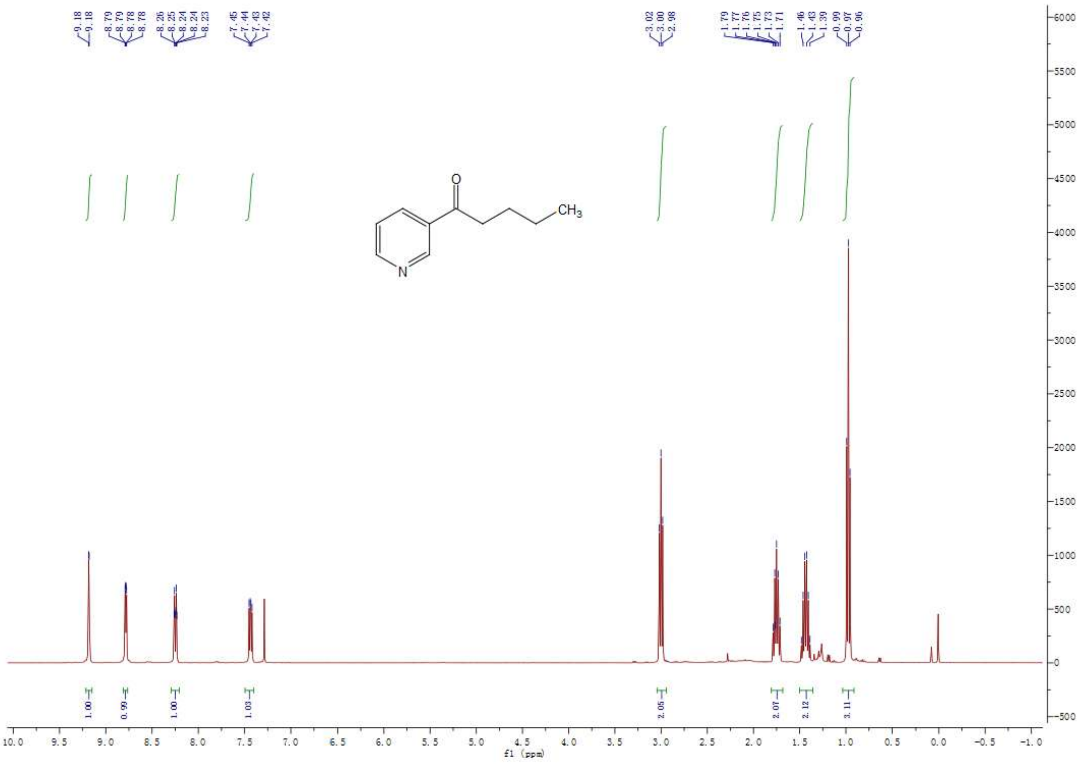

The NMR spectra of 1g

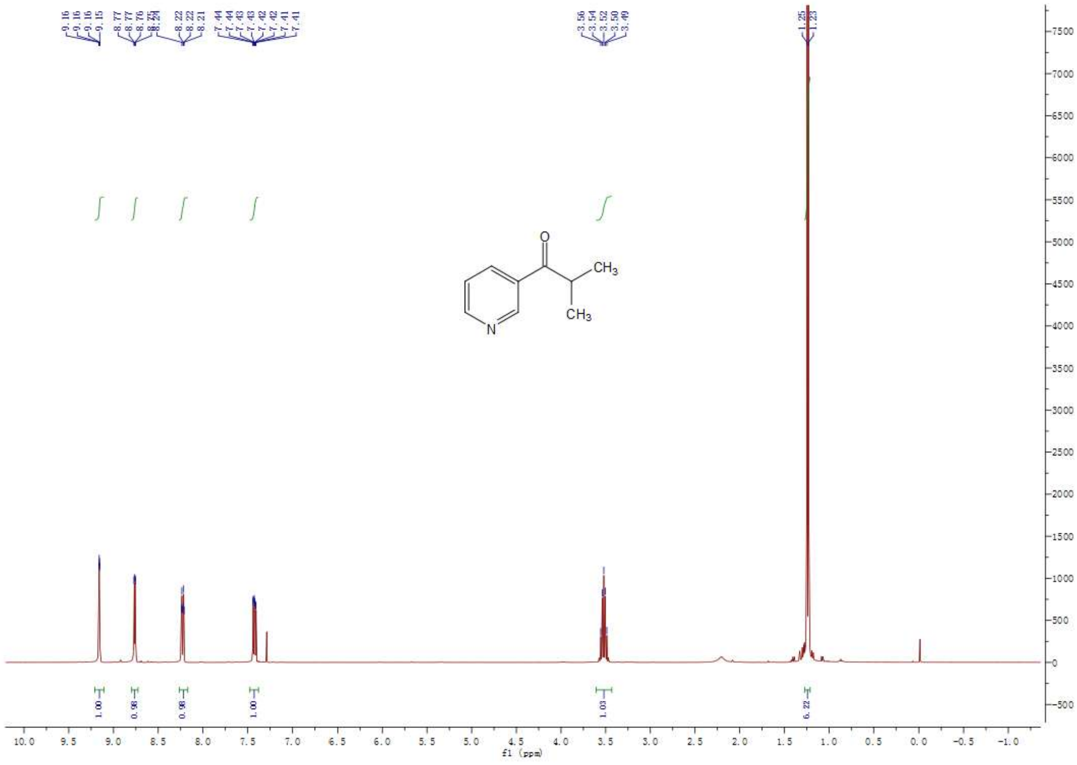

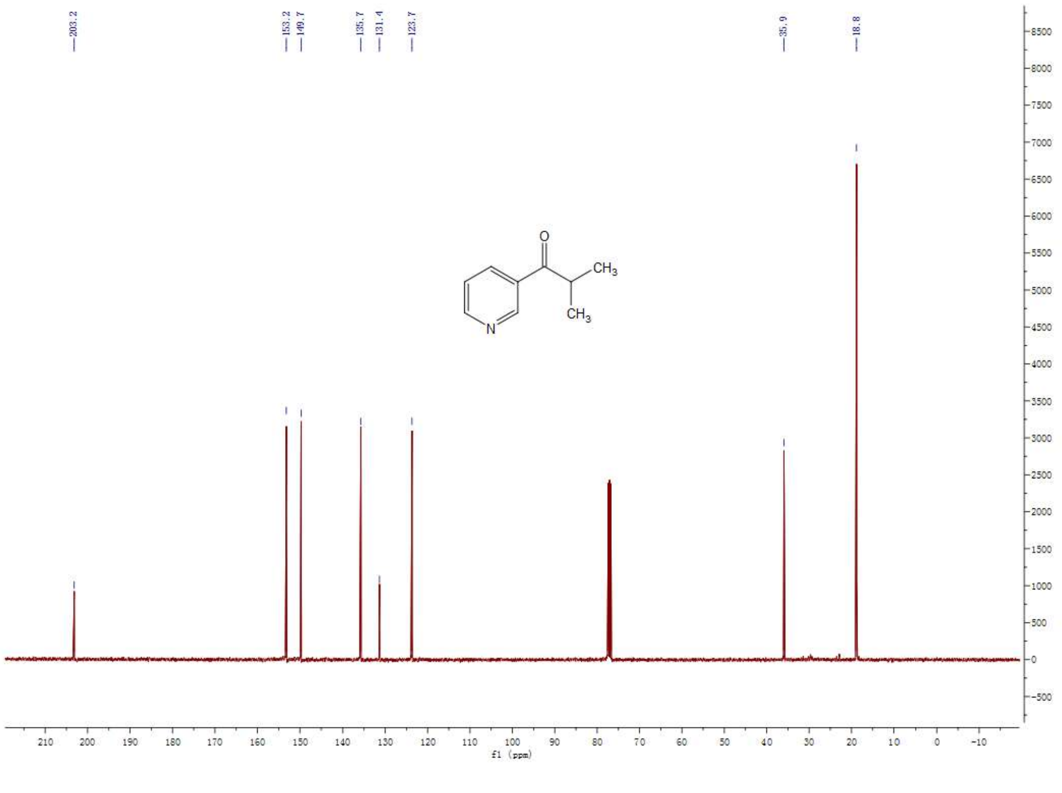

The NMR spectra of 1h

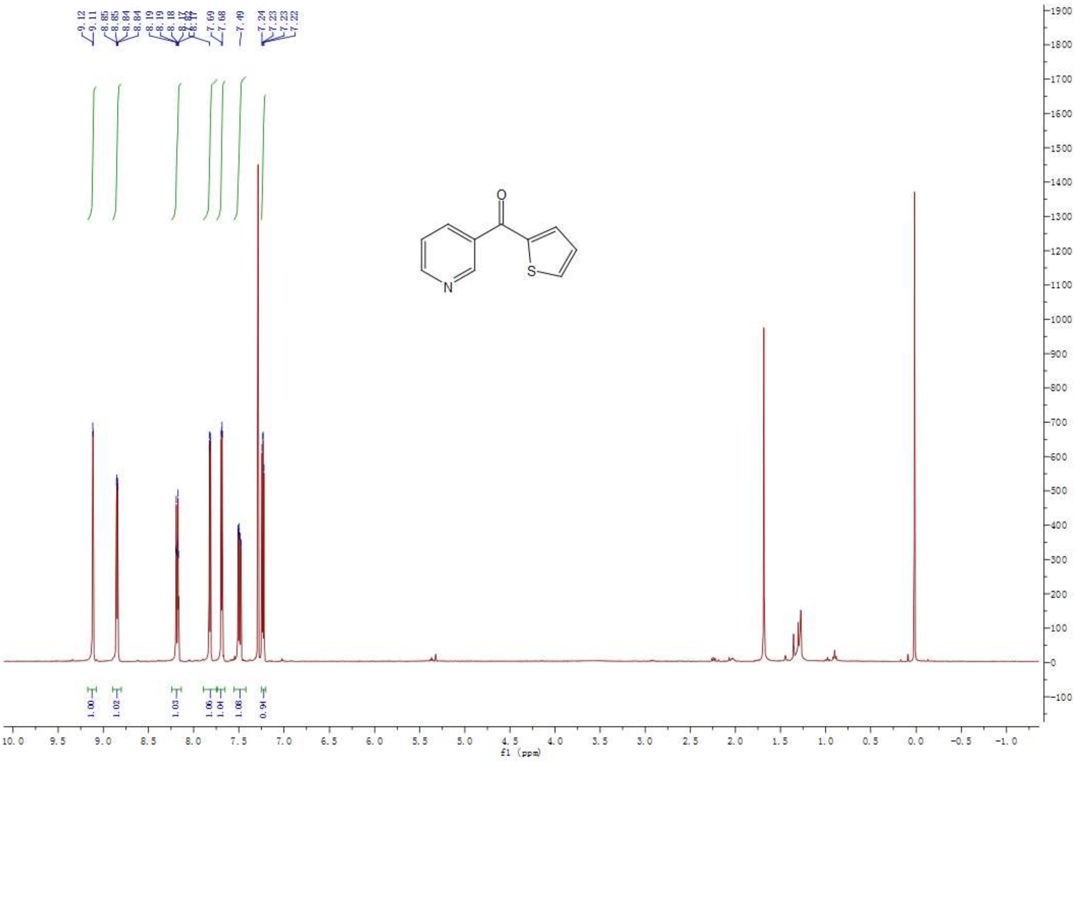

The NMR spectra of 1i

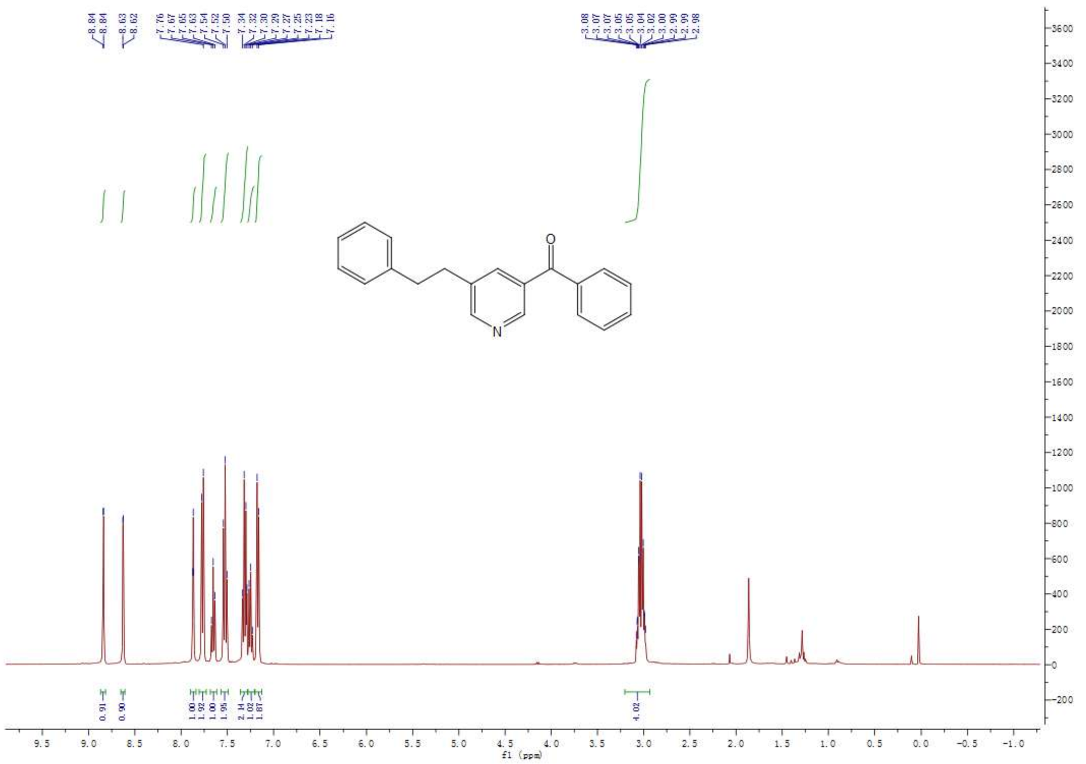

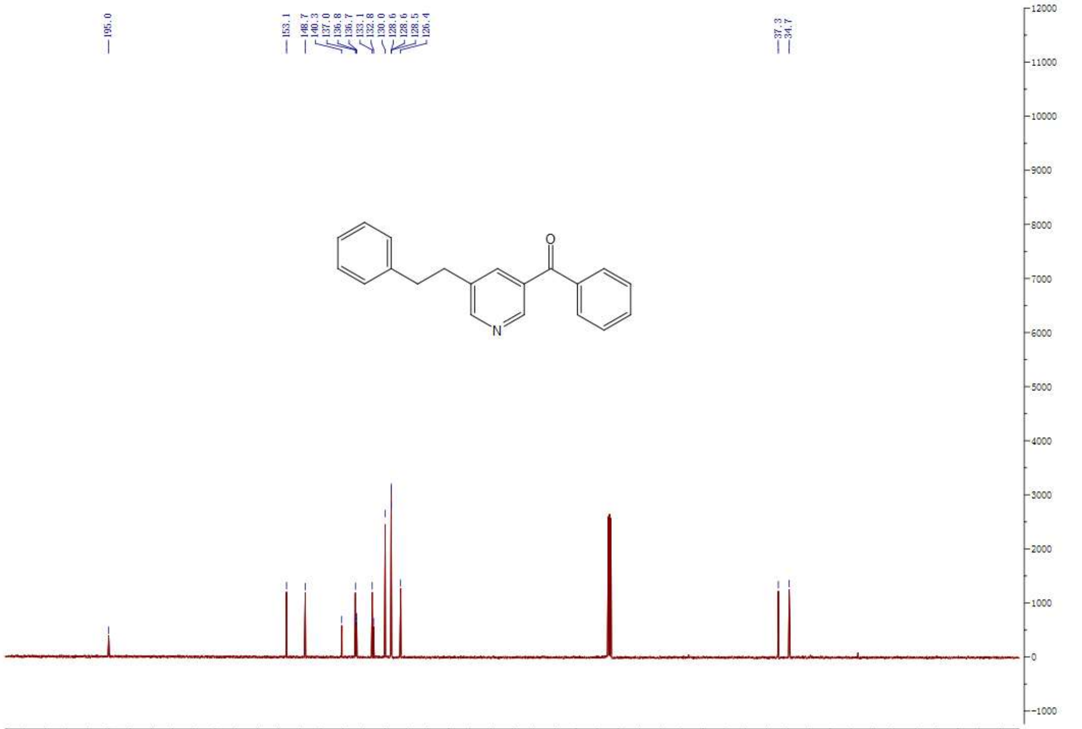

The NMR spectra of 1j

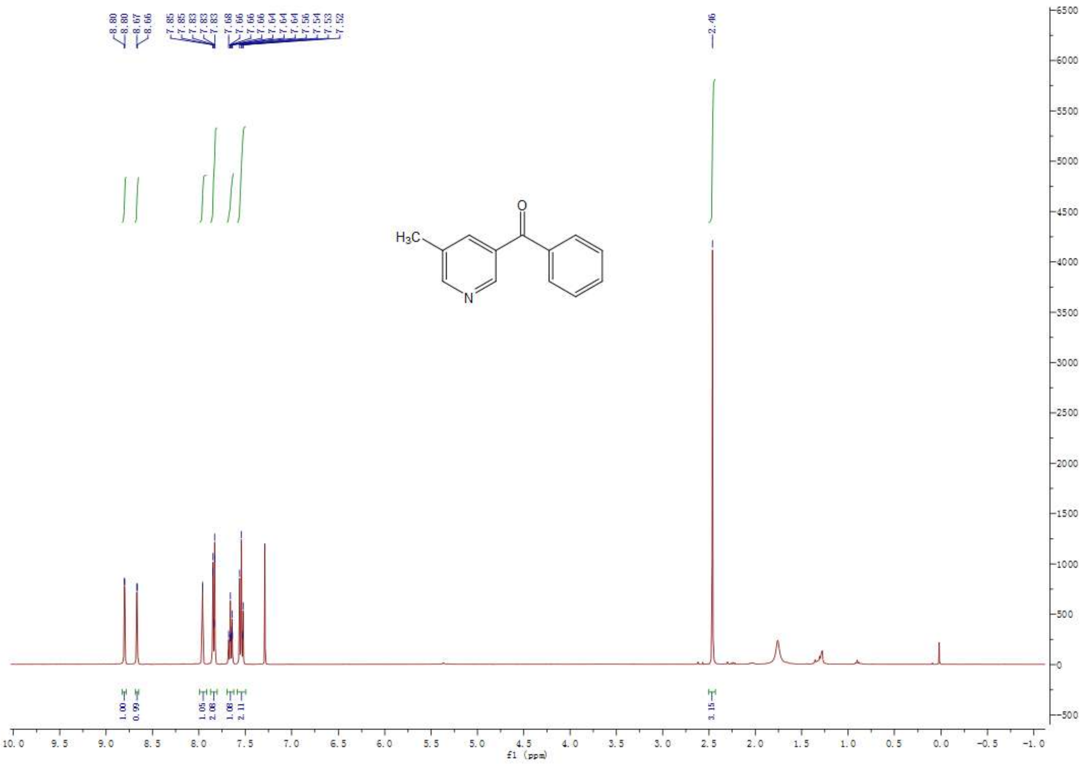

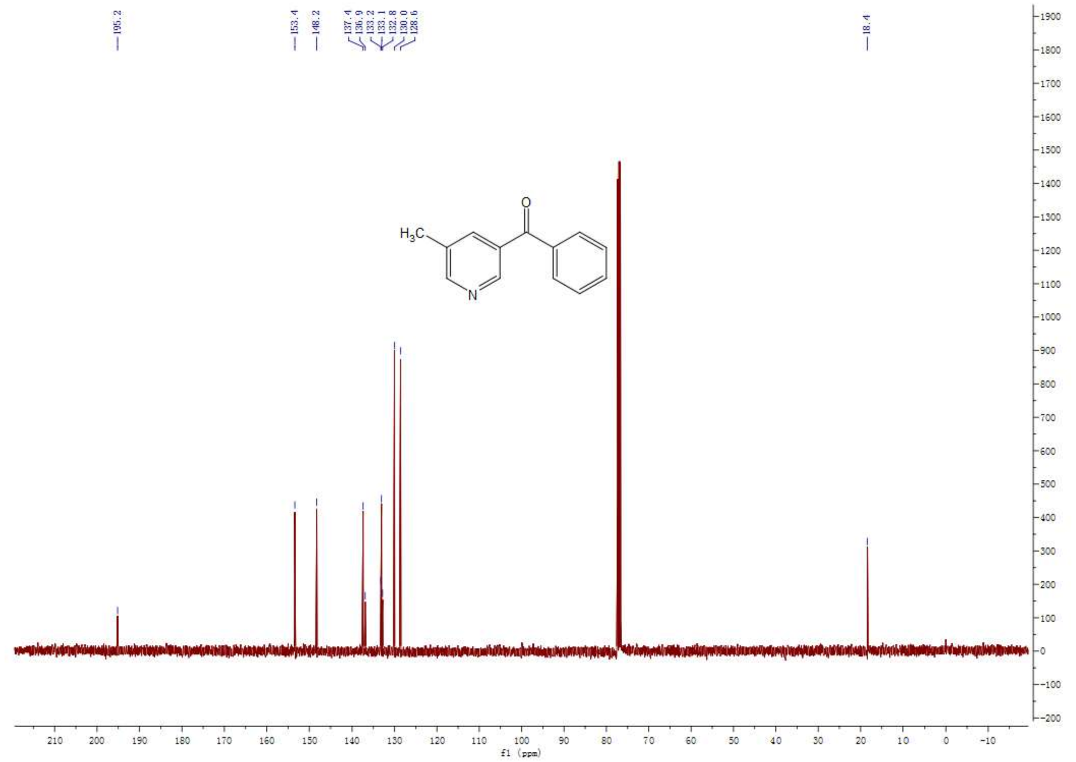

The NMR spectra of 1k

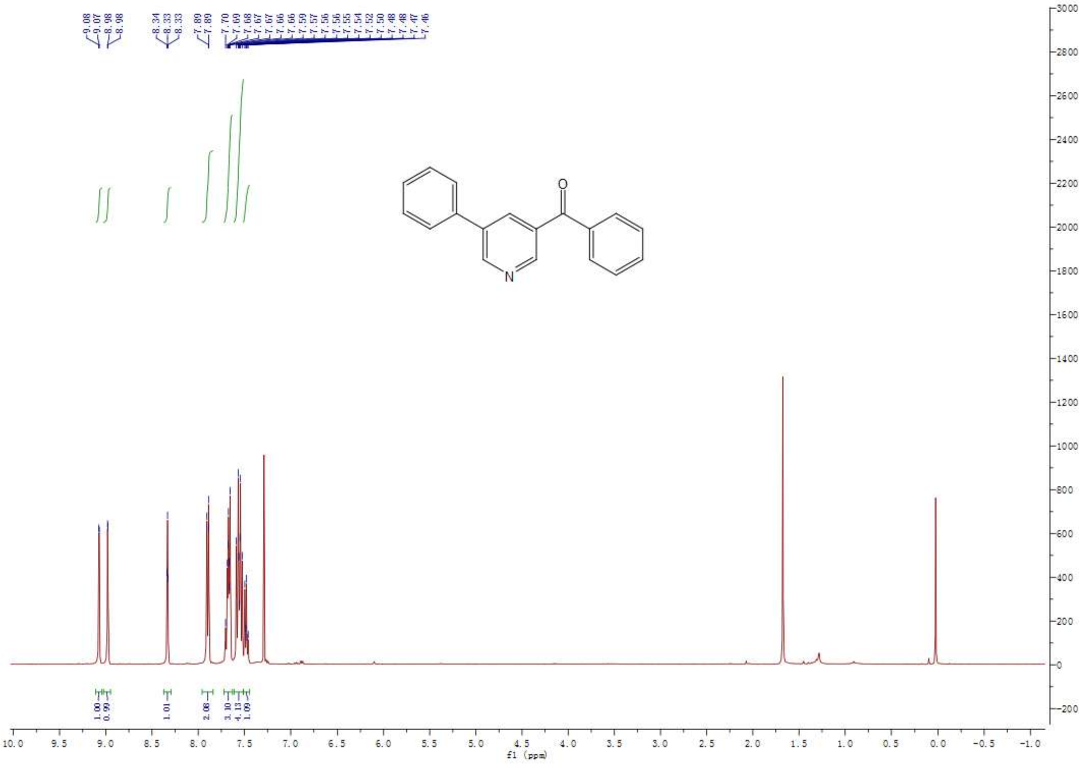

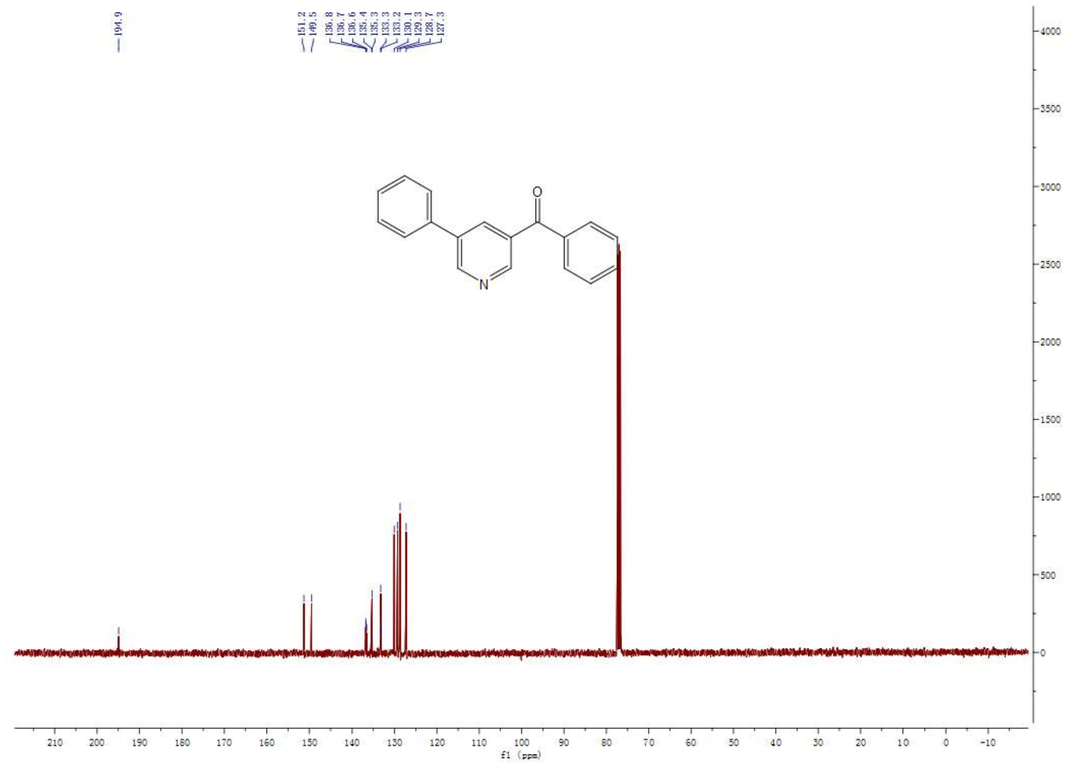

The NMR spectra of 1l

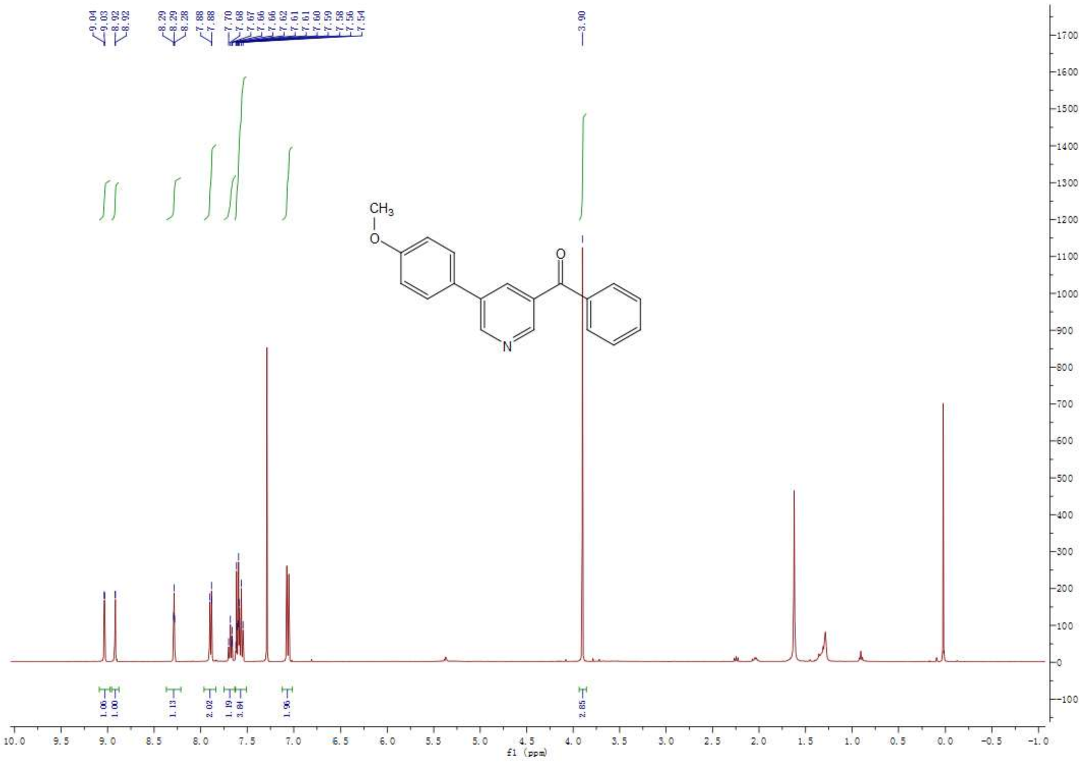

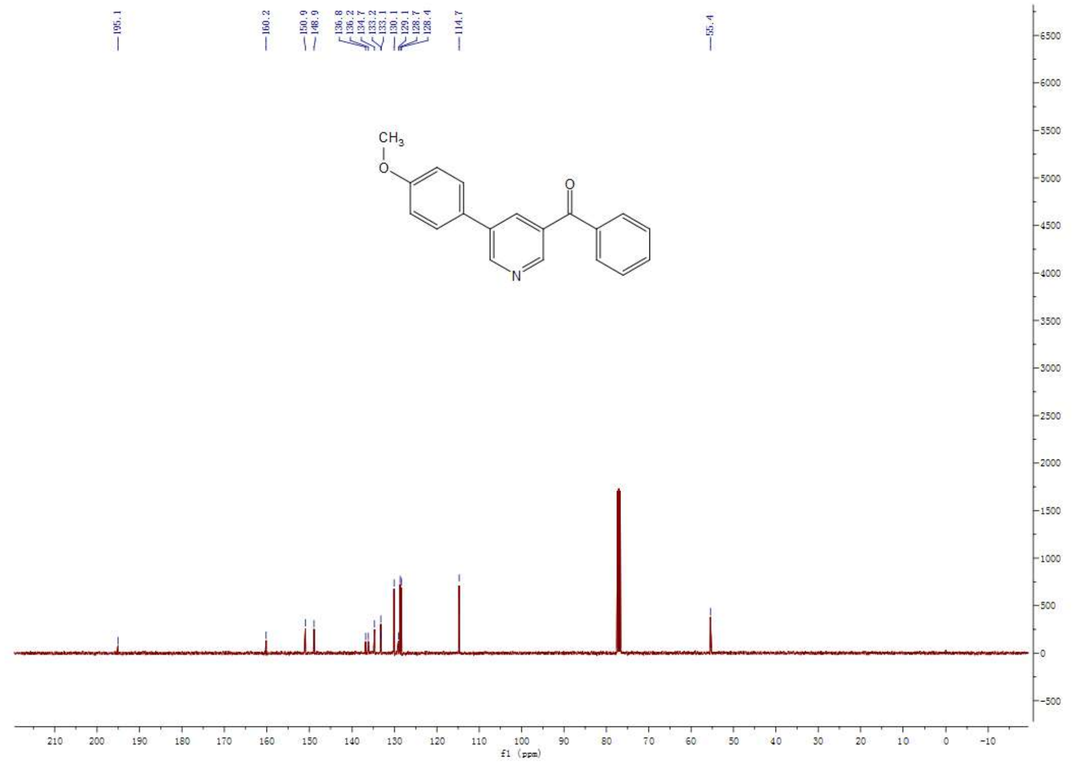

The NMR spectra of 1m

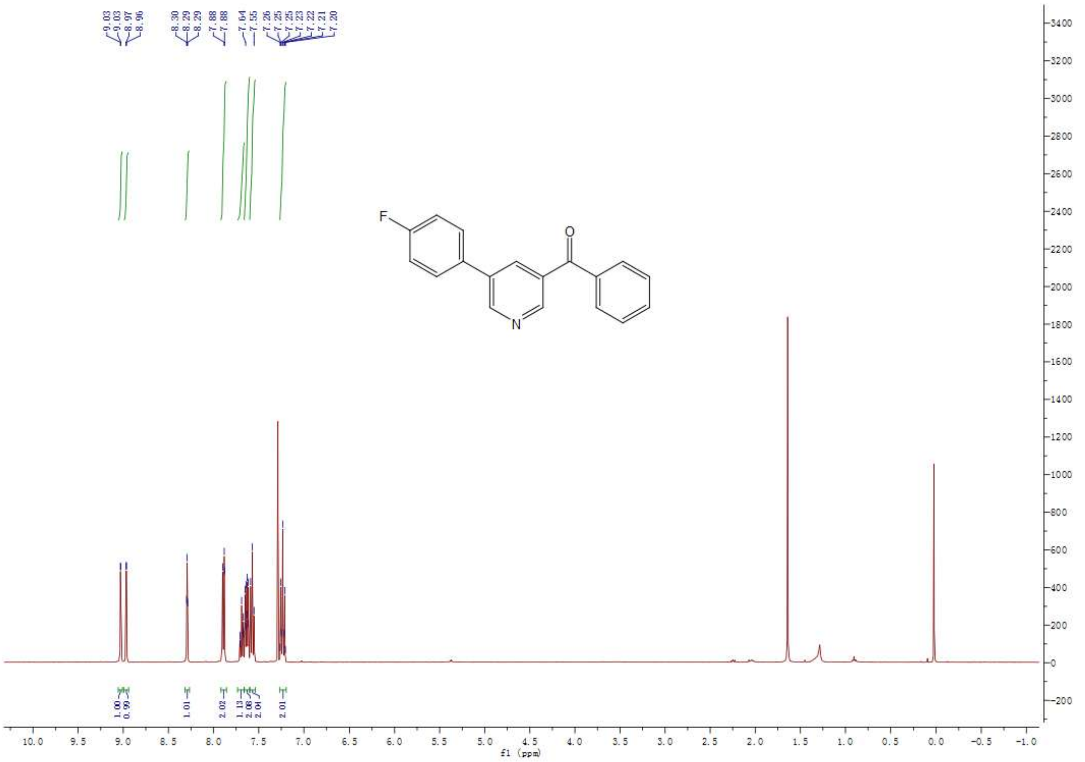

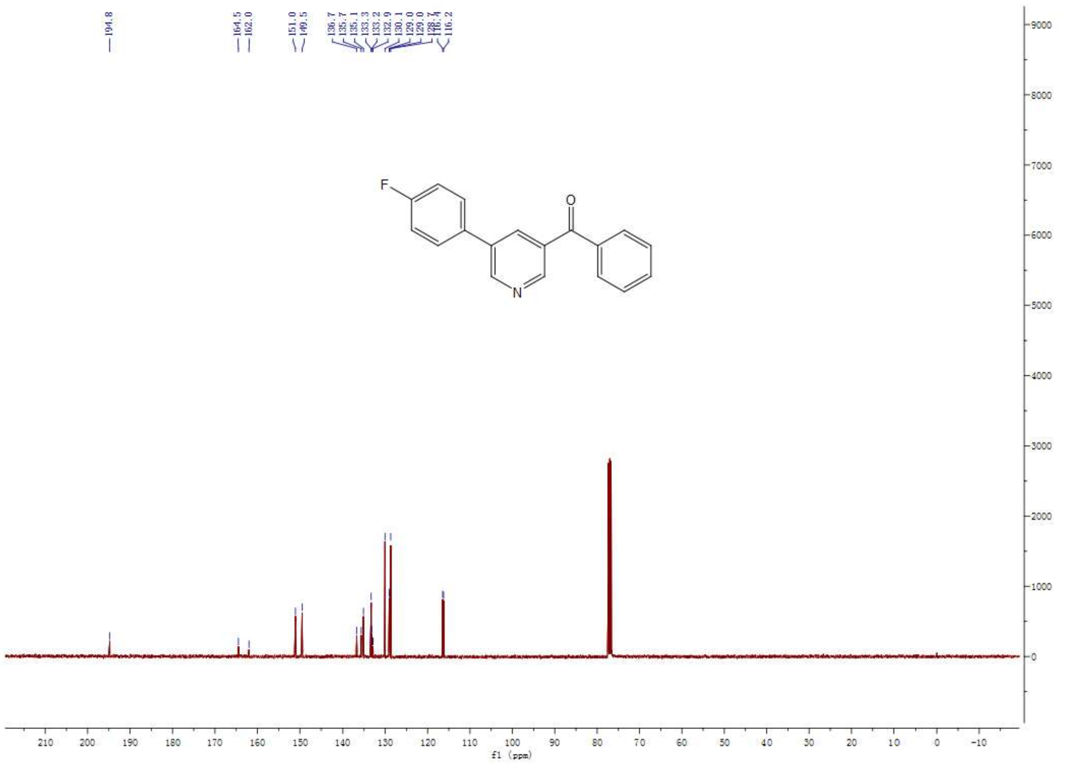

The NMR spectra of 1n

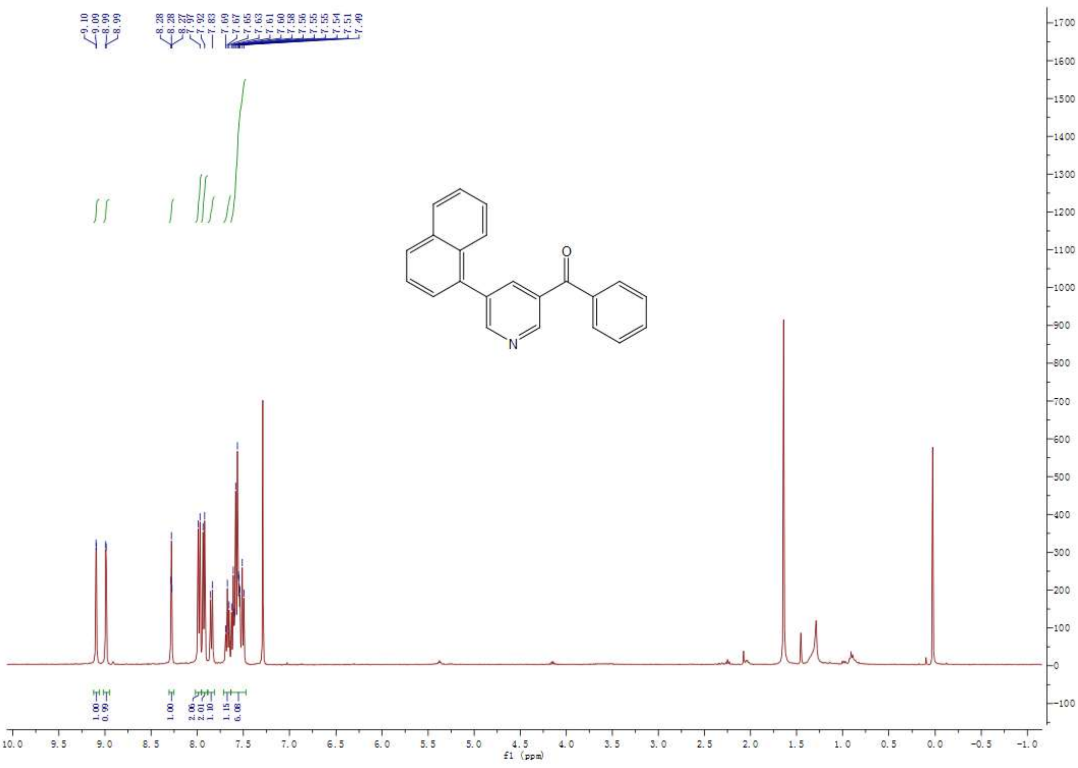

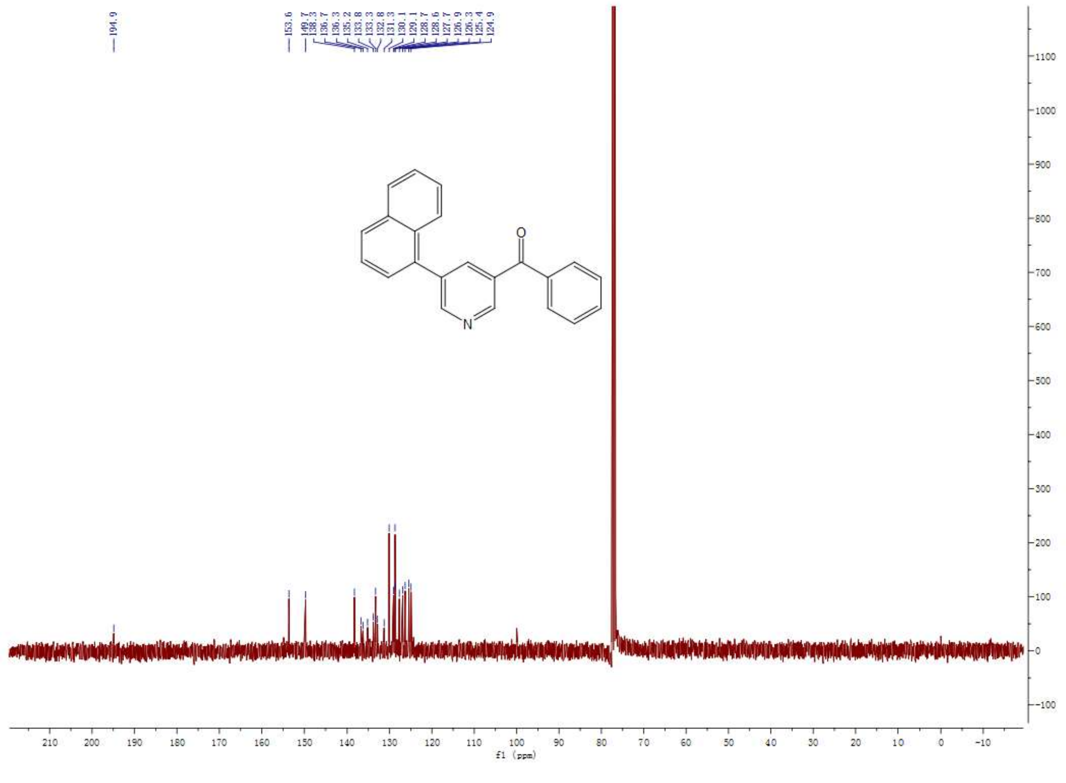

The NMR spectra of 1o

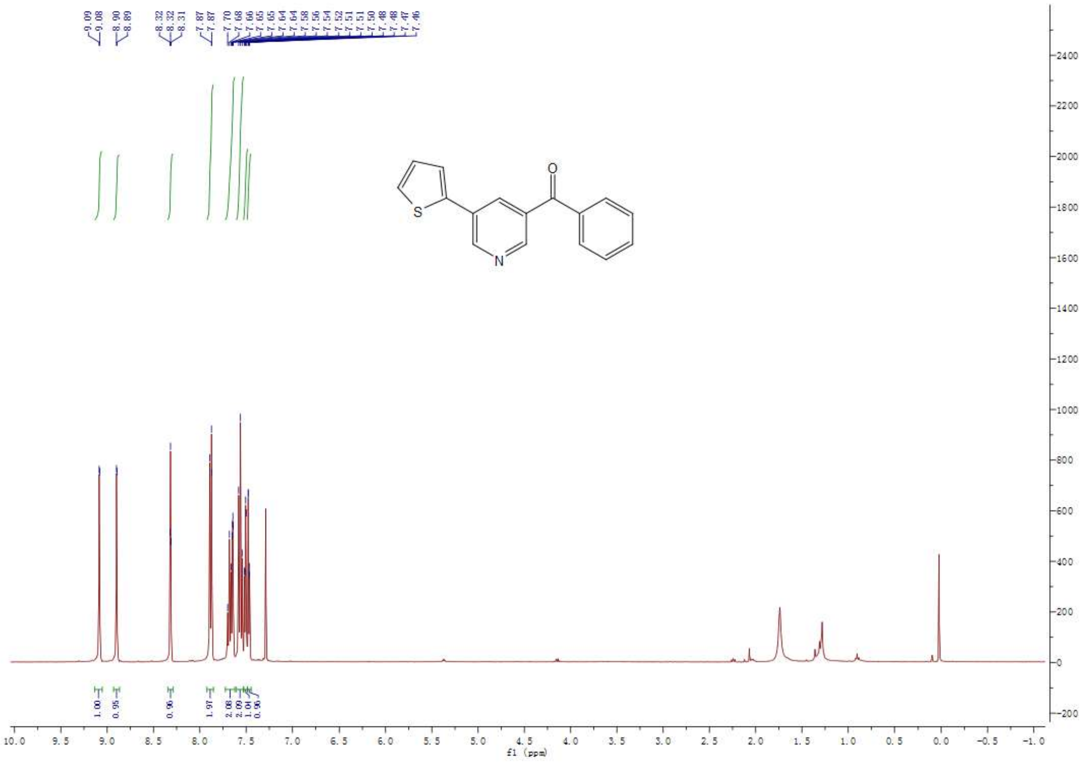

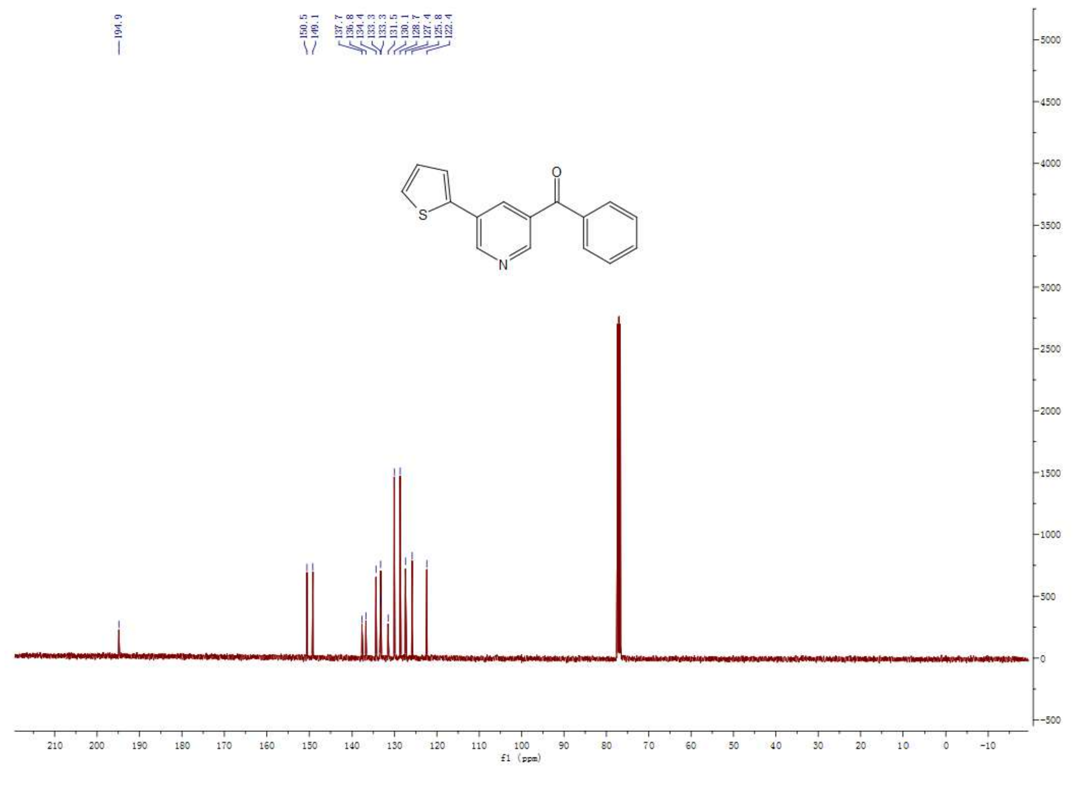

The NMR spectra of 1p

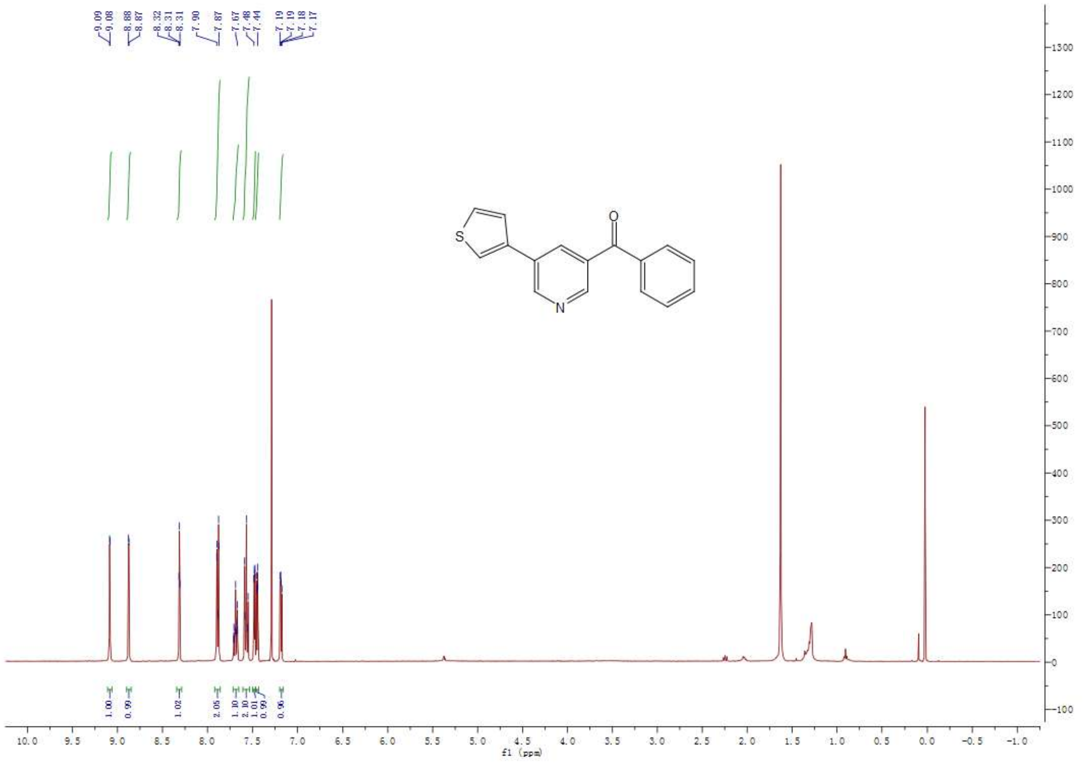

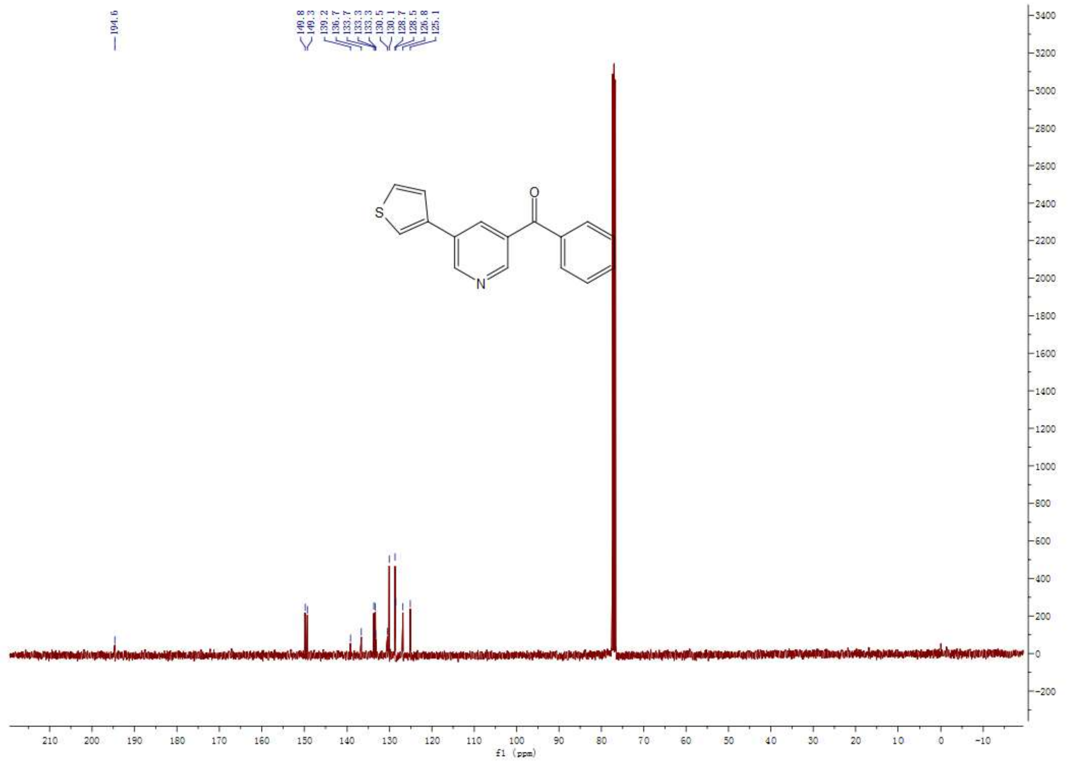

The NMR spectra of 1q

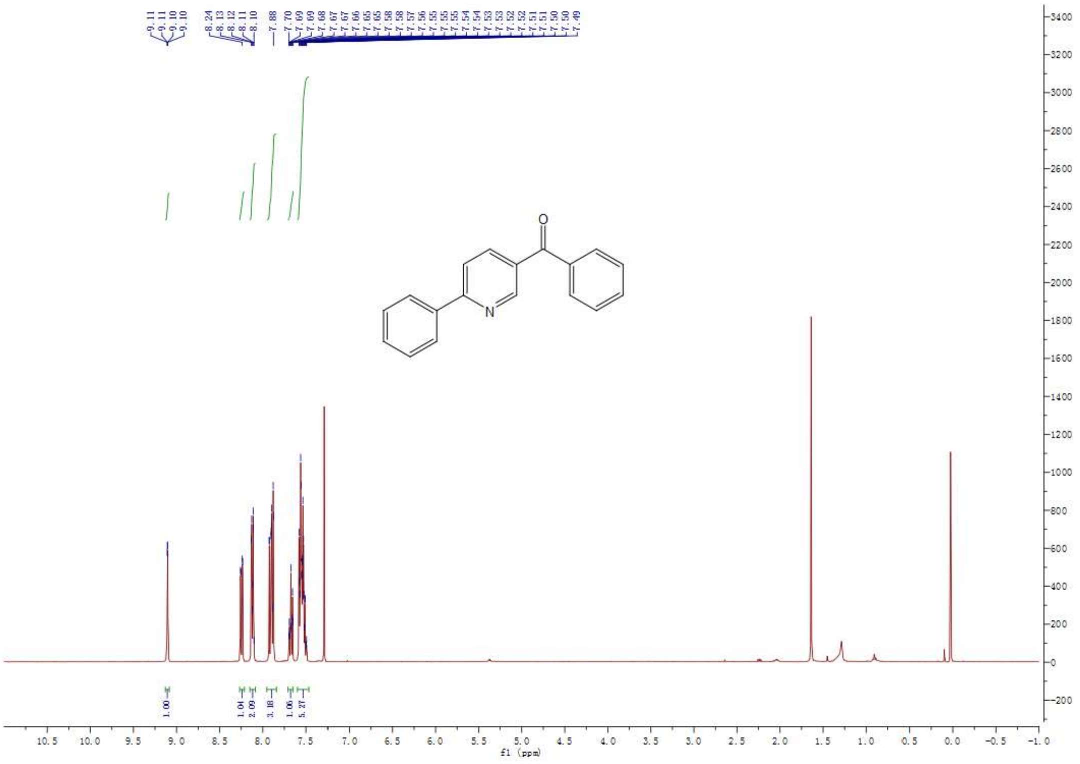

The NMR spectra of 1r

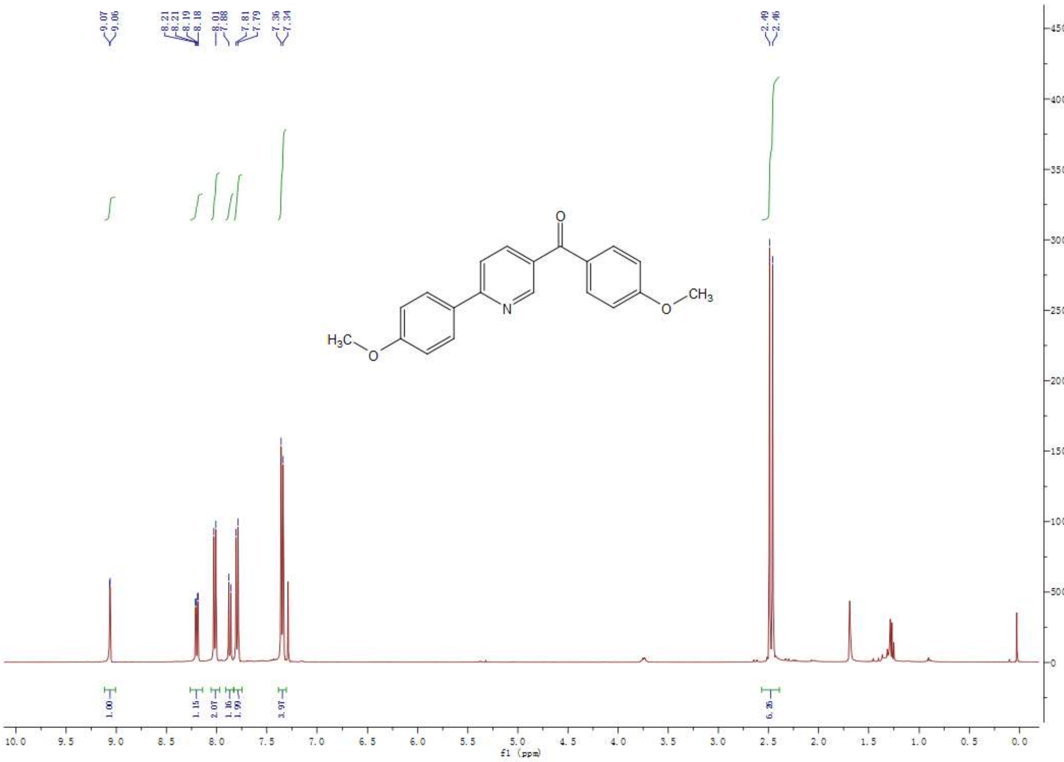

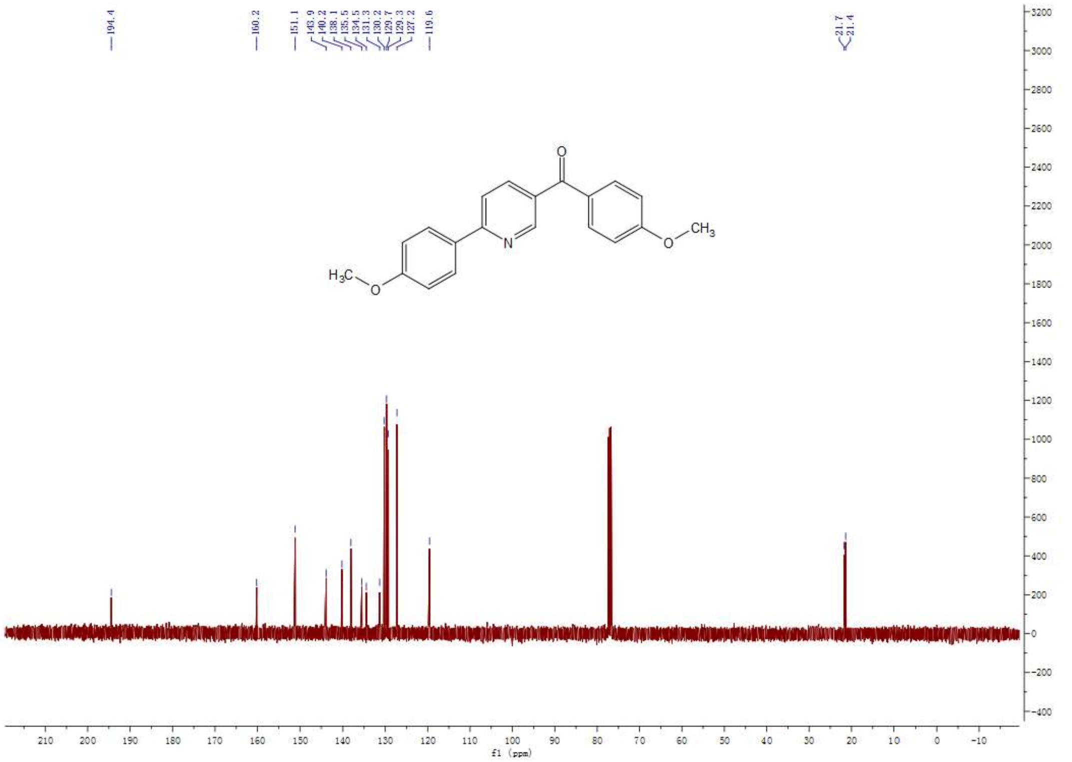

The NMR spectra of 1s

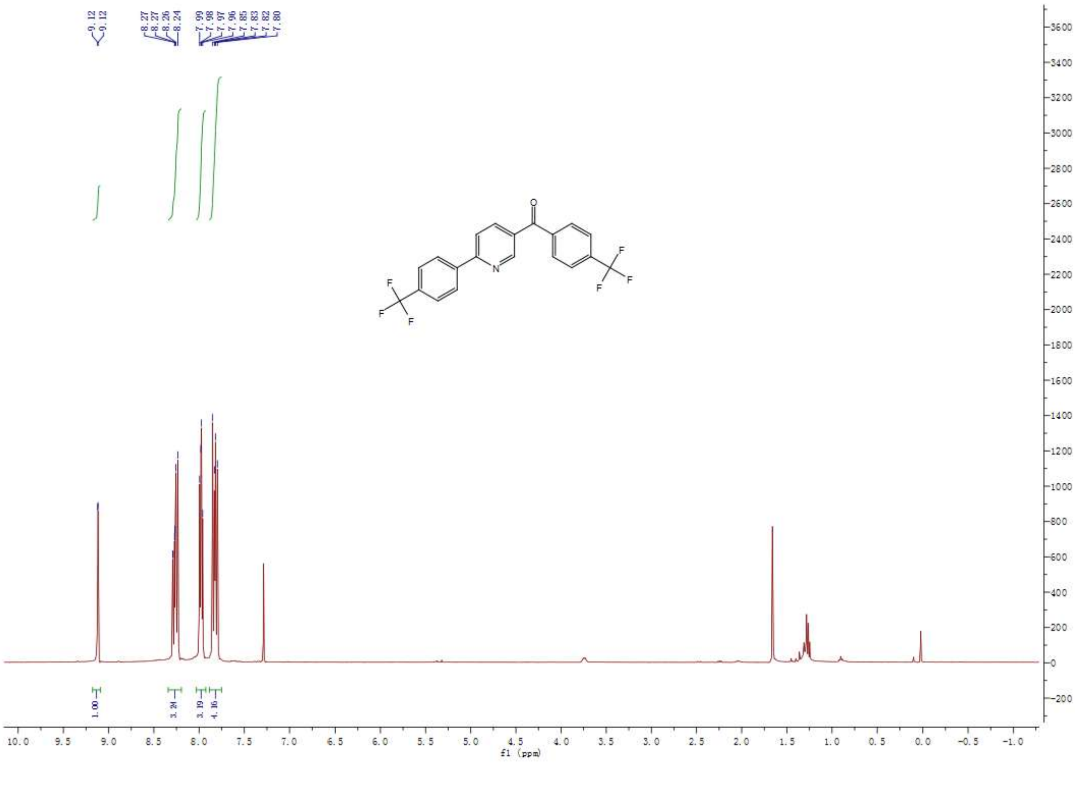

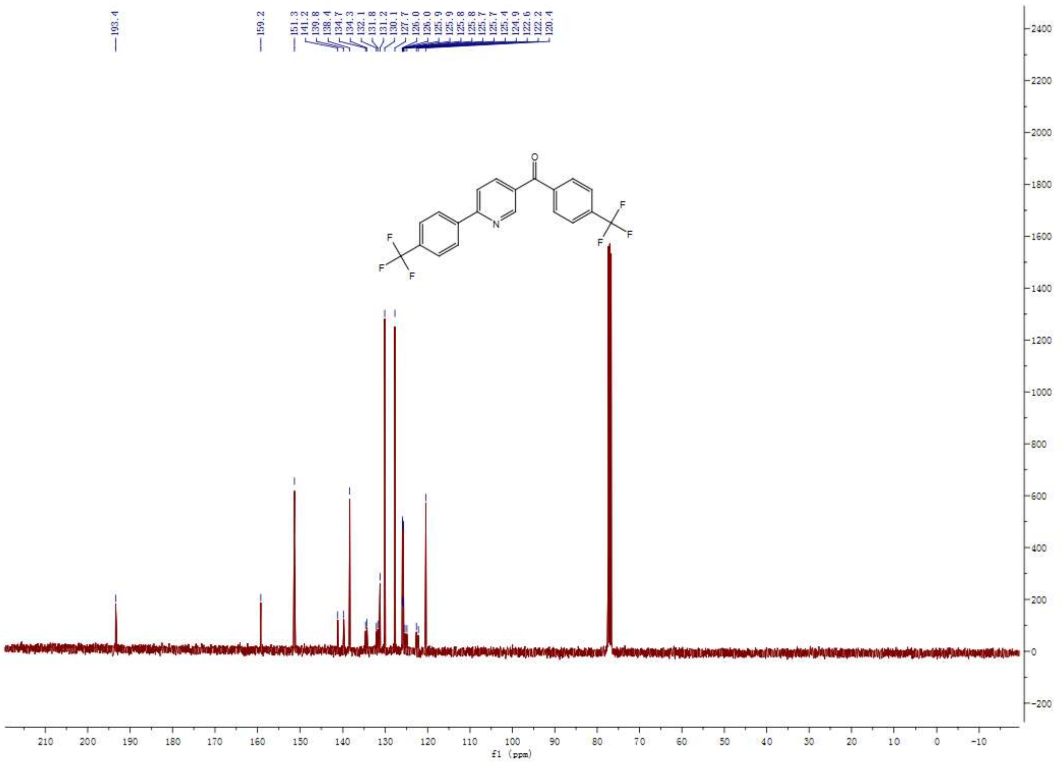

The NMR spectra of 1u

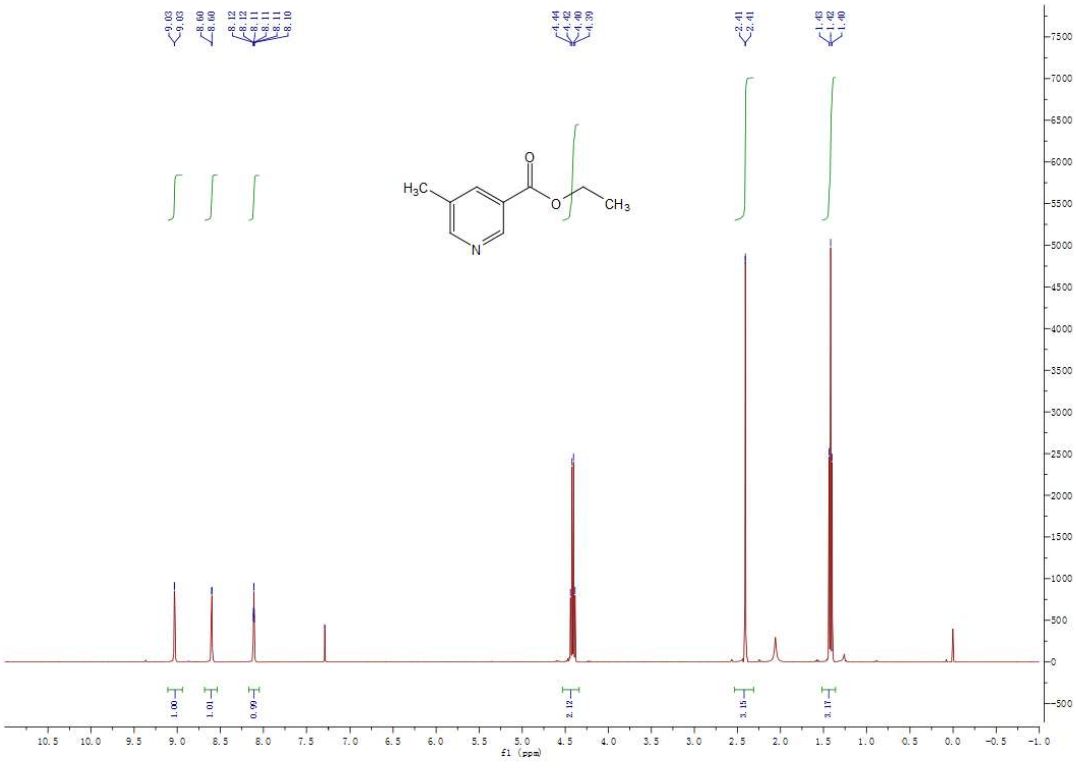

The NMR spectra of 1v

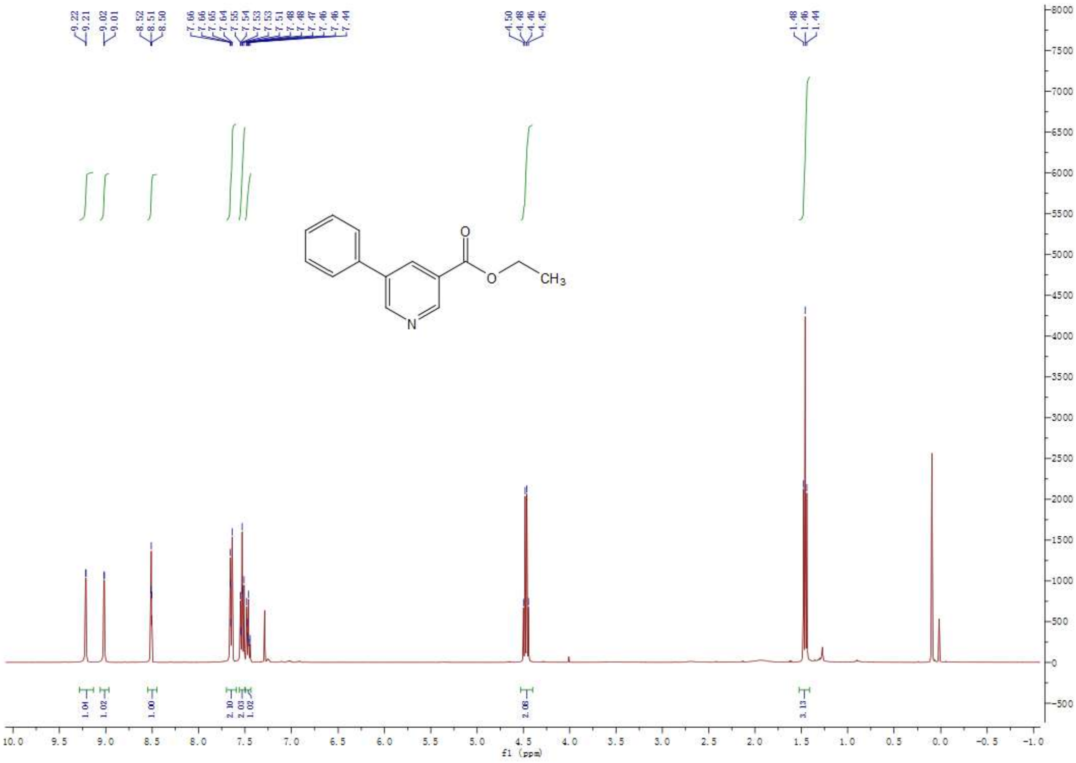

The NMR spectra of 1w

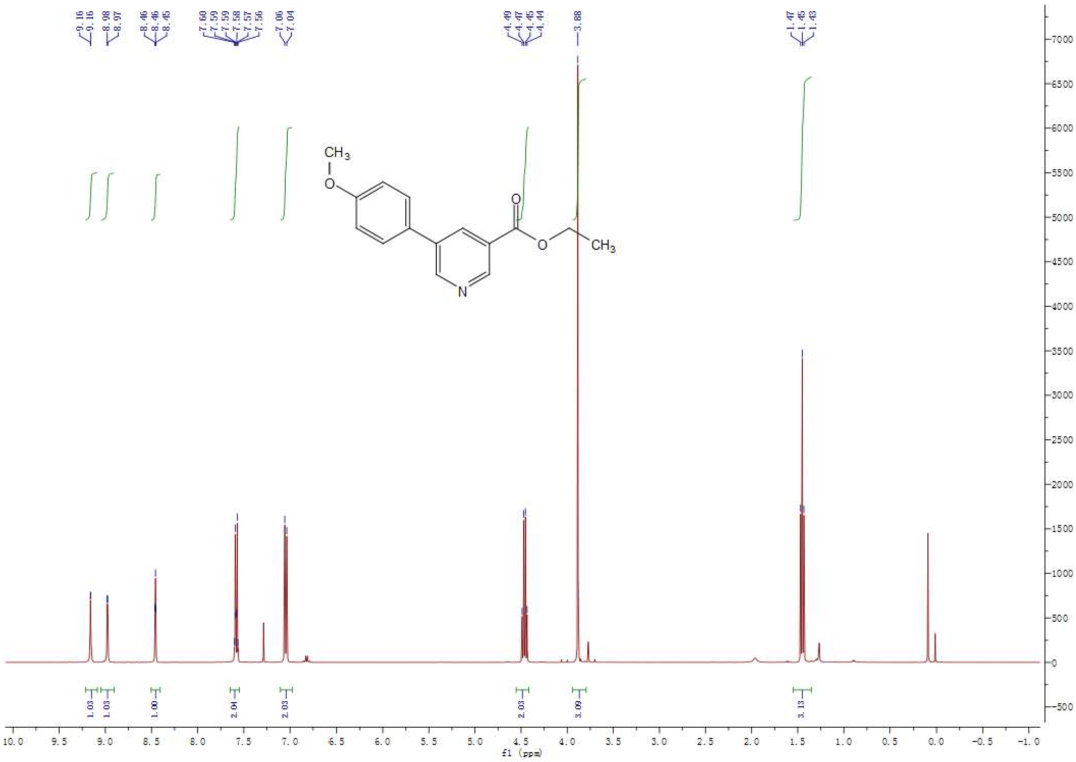

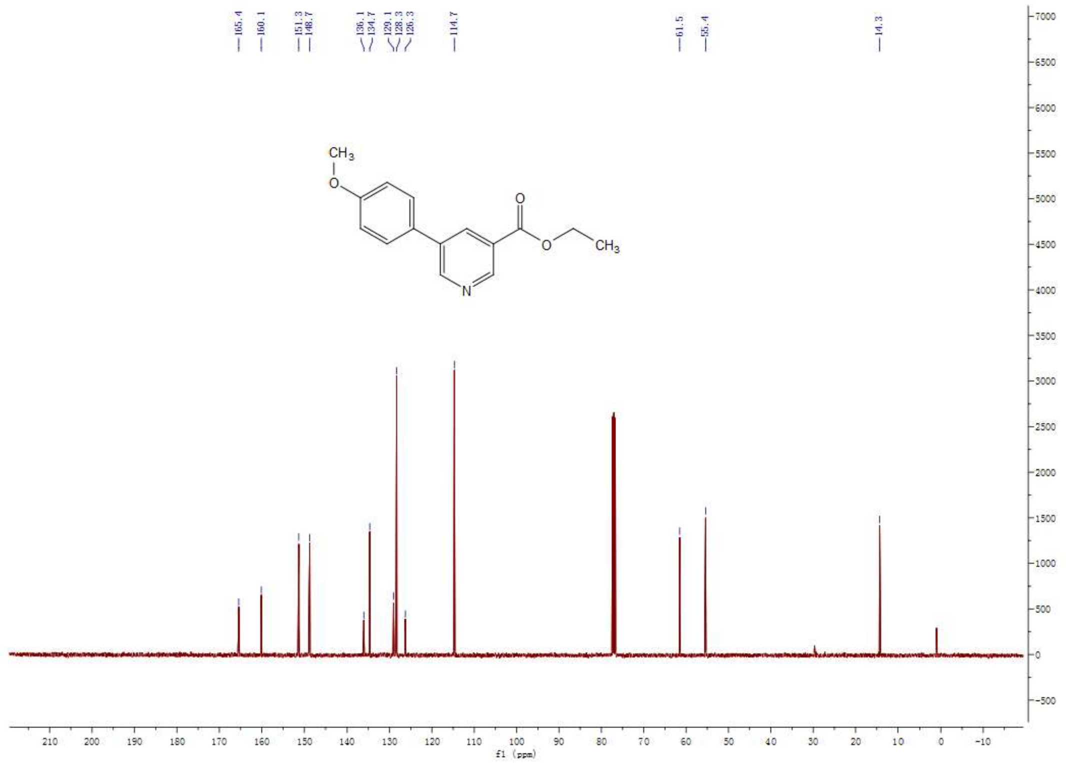

The NMR spectra of 1x

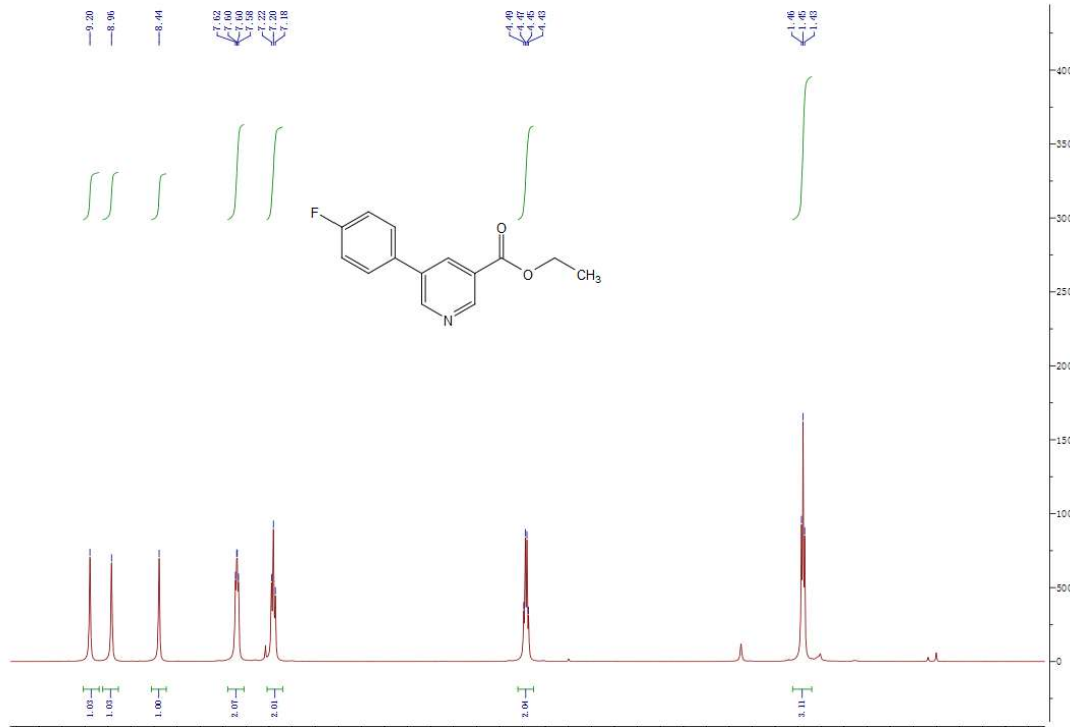

The NMR spectra of 1y

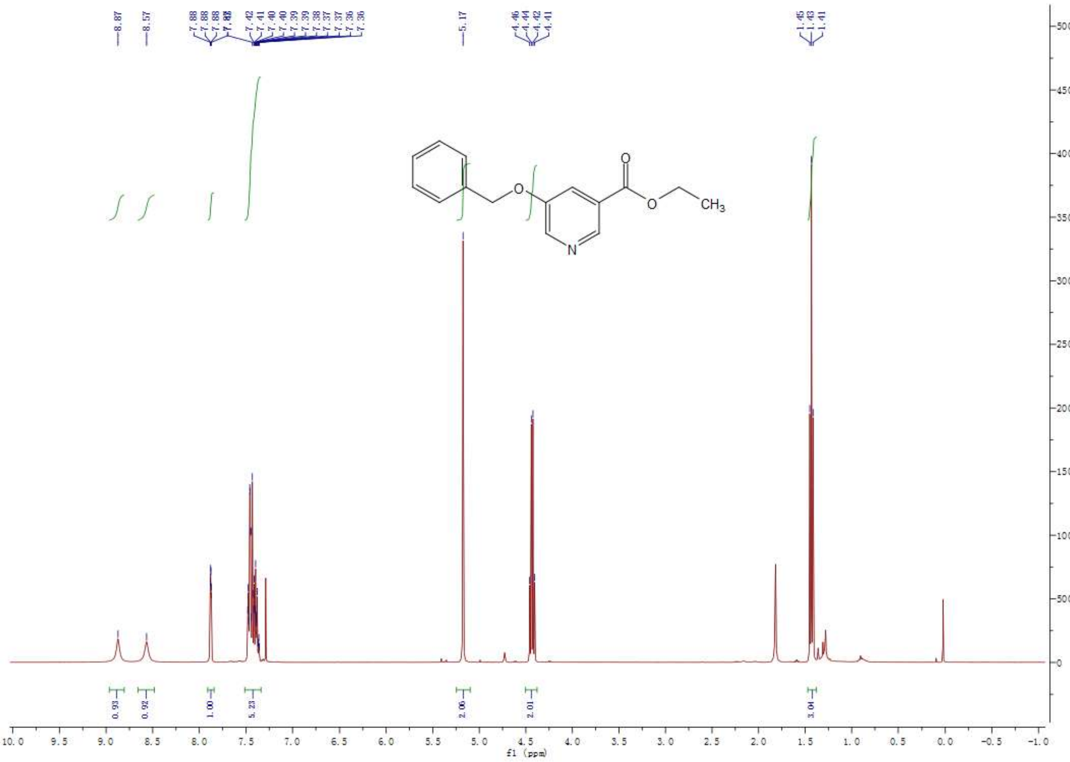

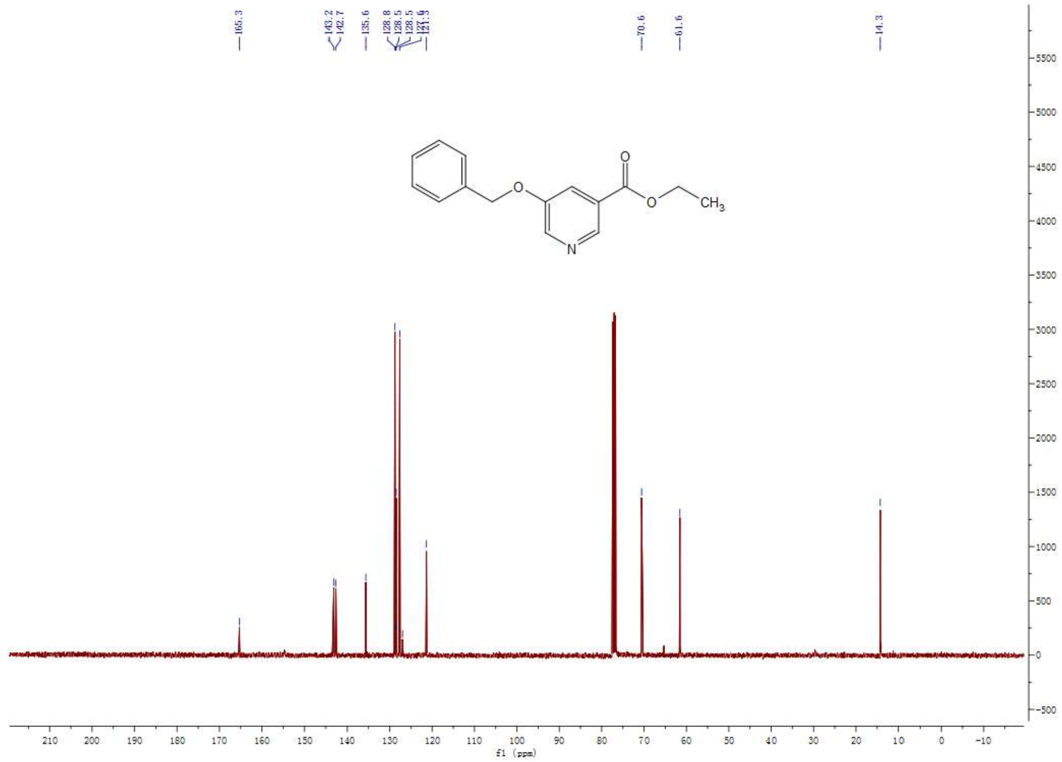

The NMR spectra of 2a

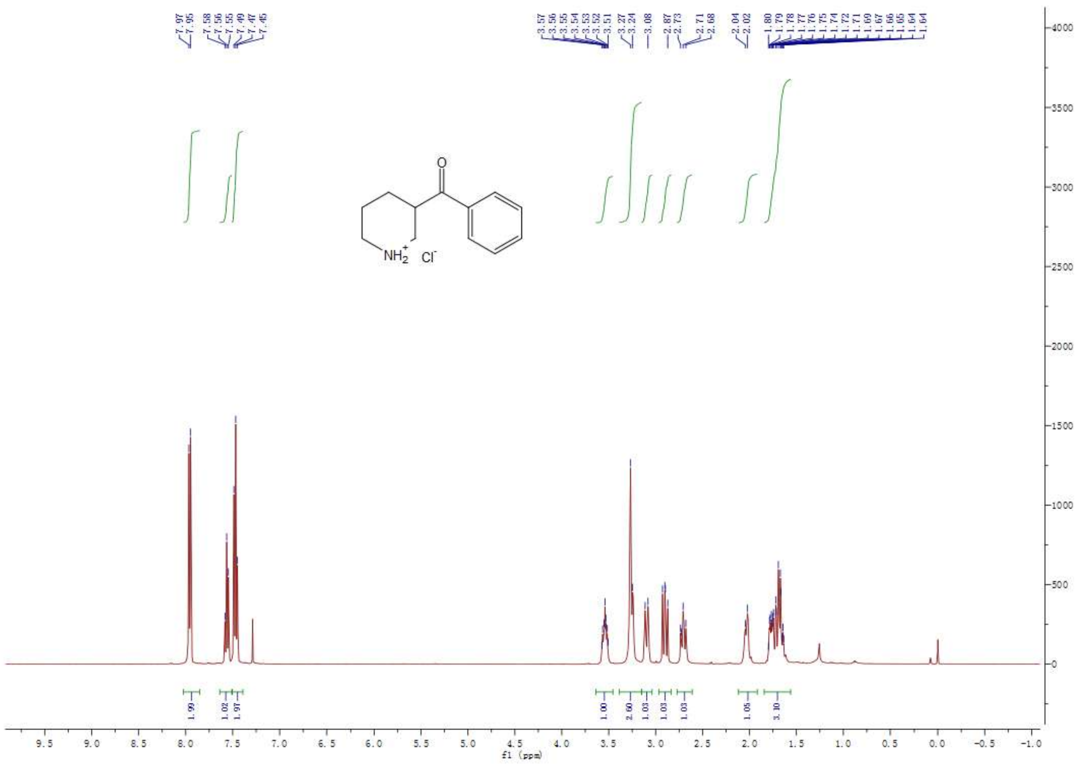

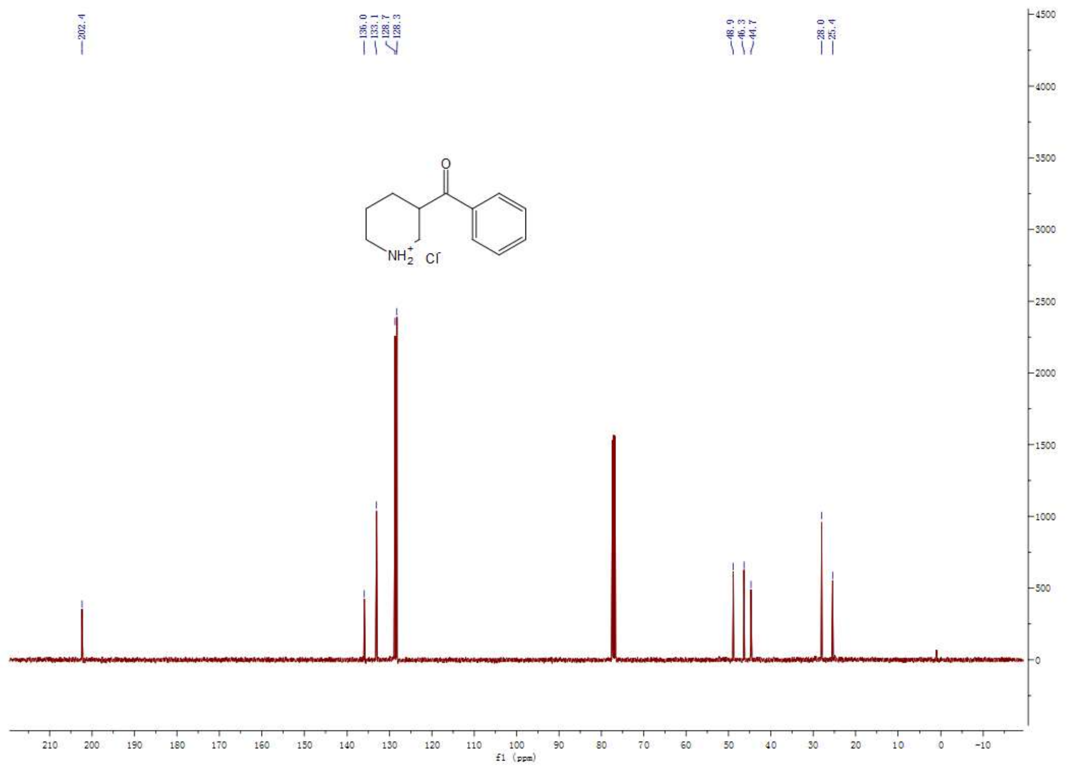

The NMR spectra of 2b

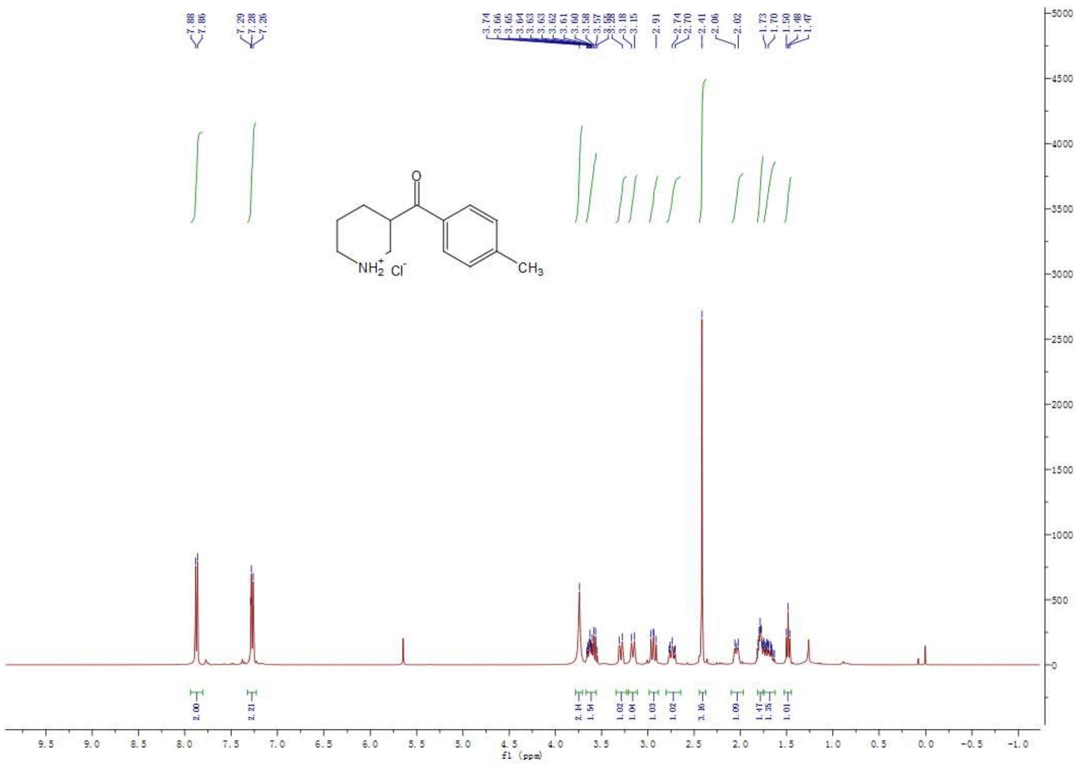

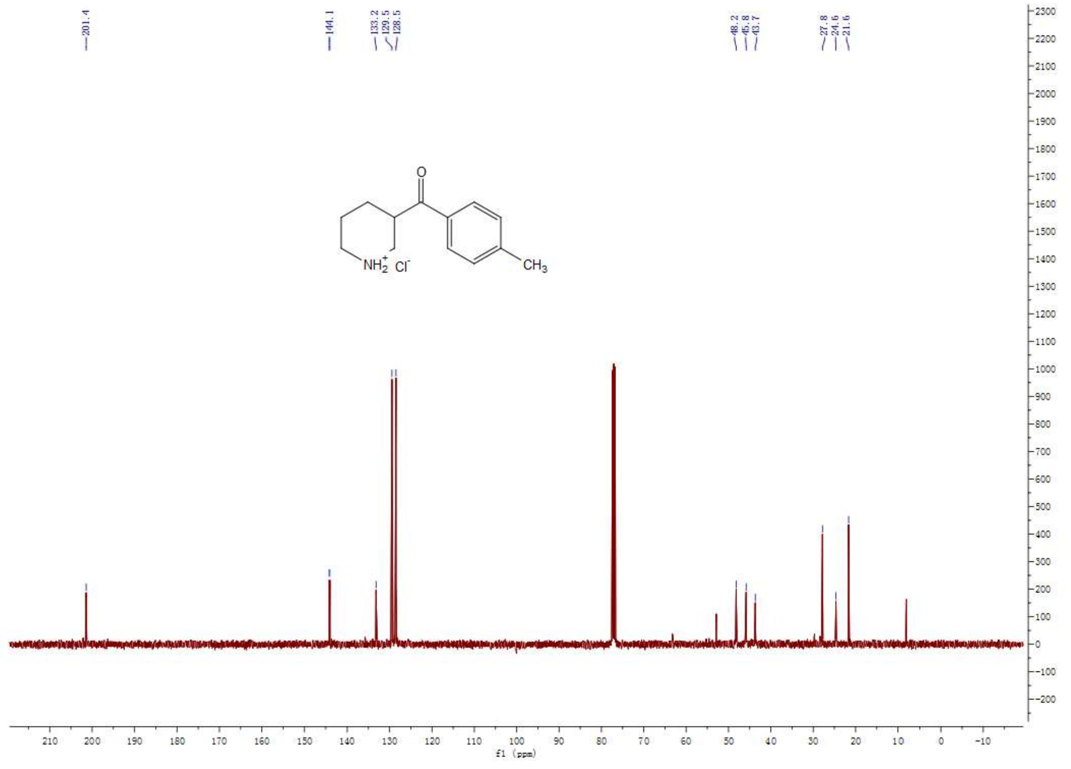

The NMR spectra of 2c

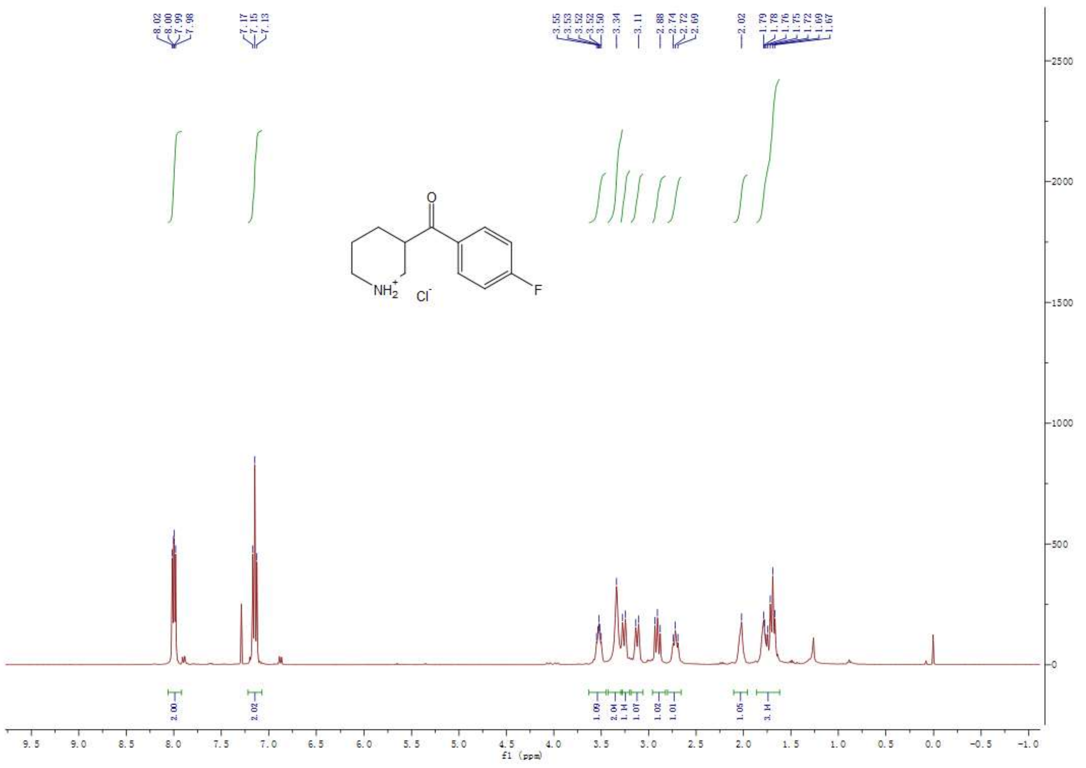

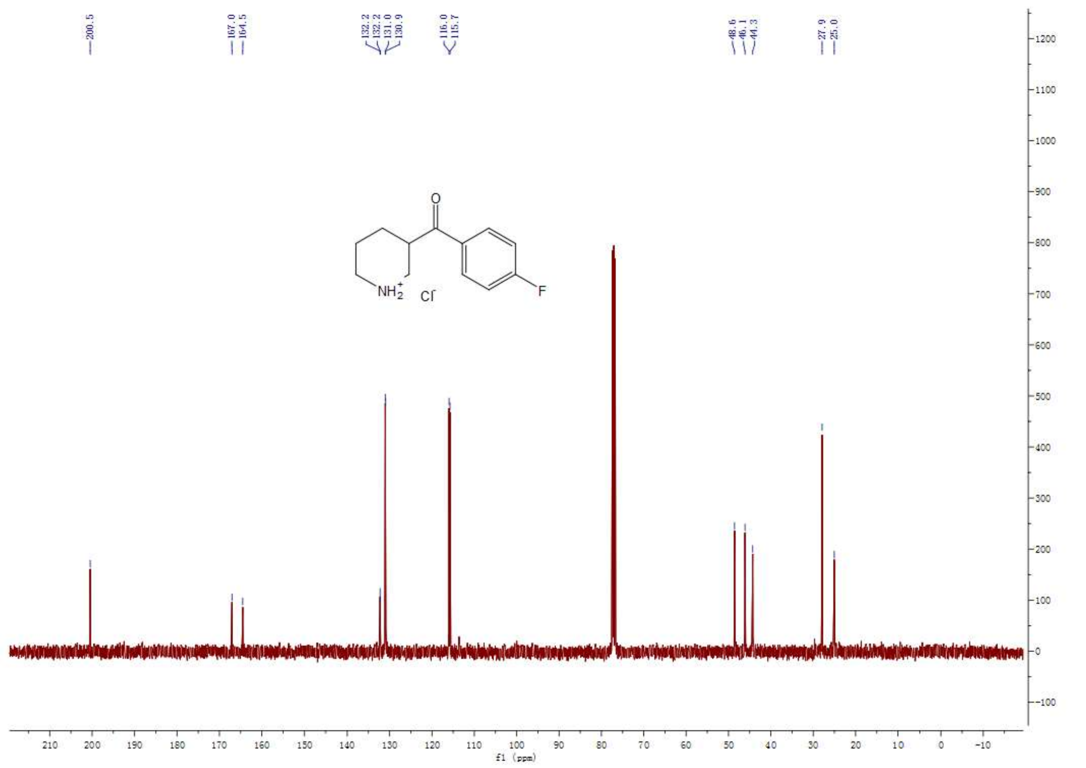

The NMR spectra of 2d

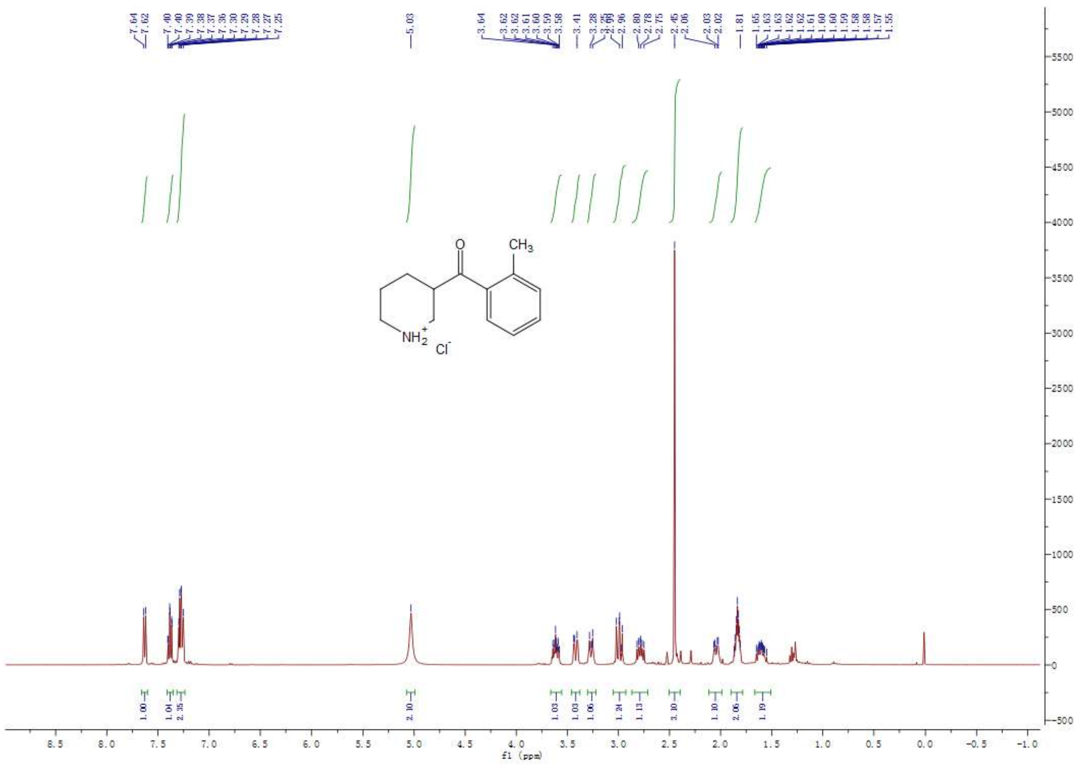

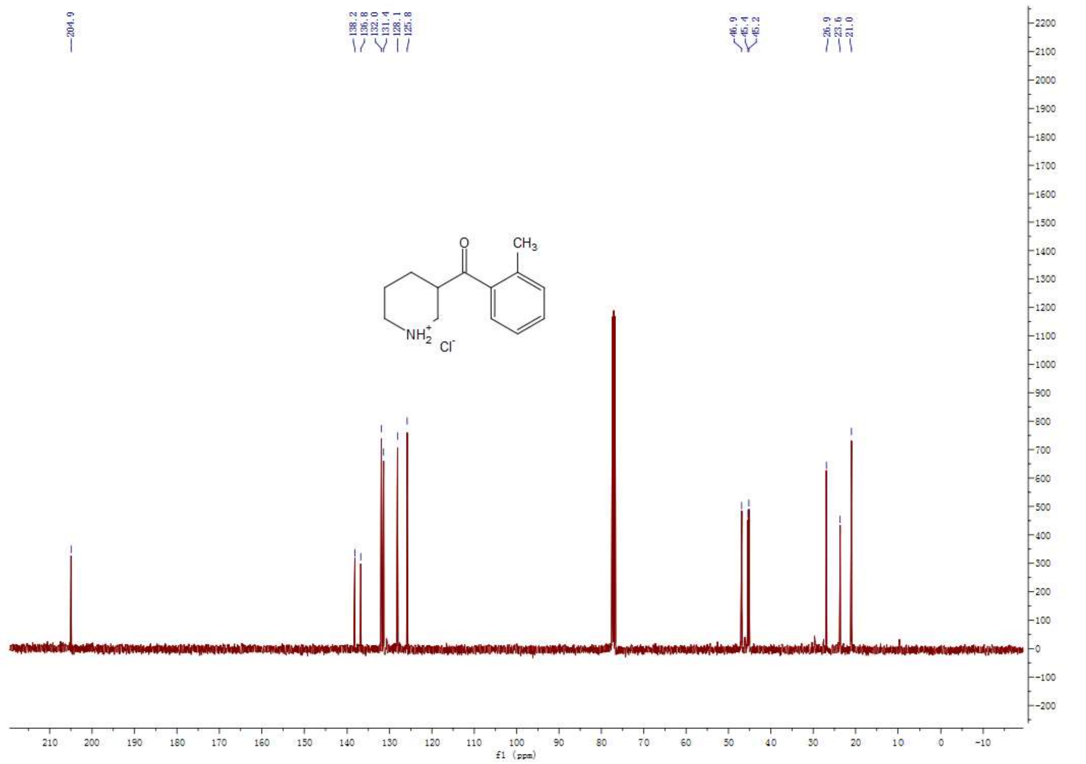

The NMR spectra of 2e

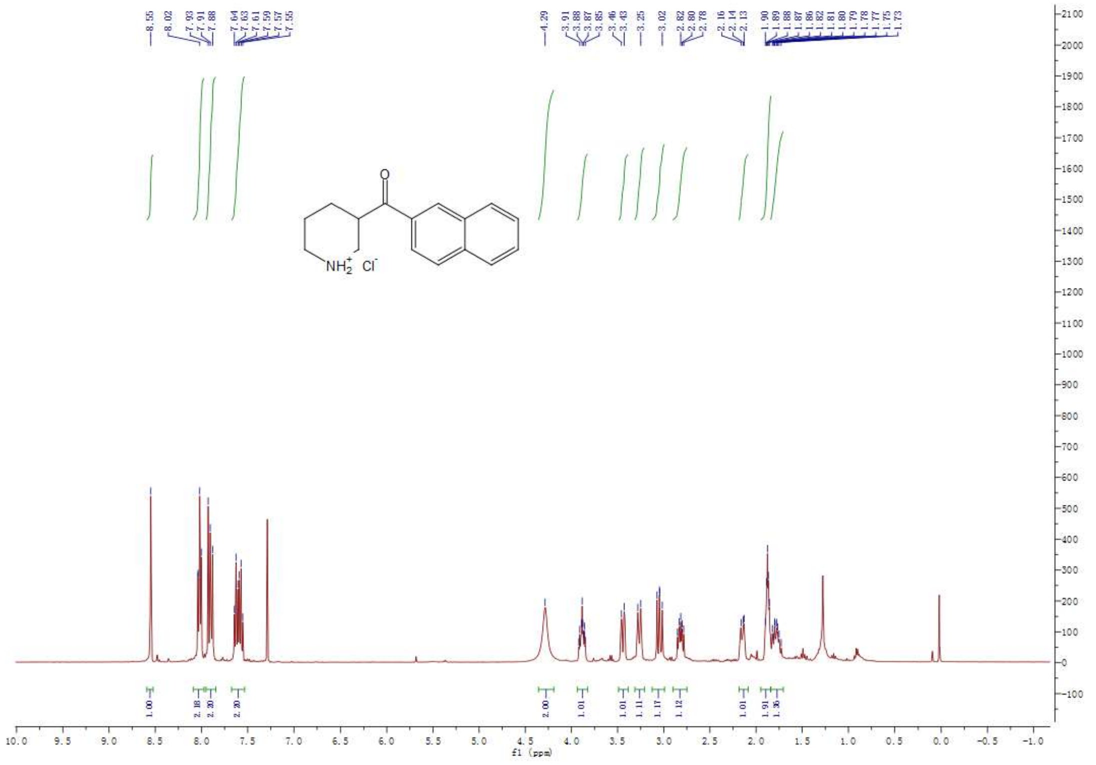

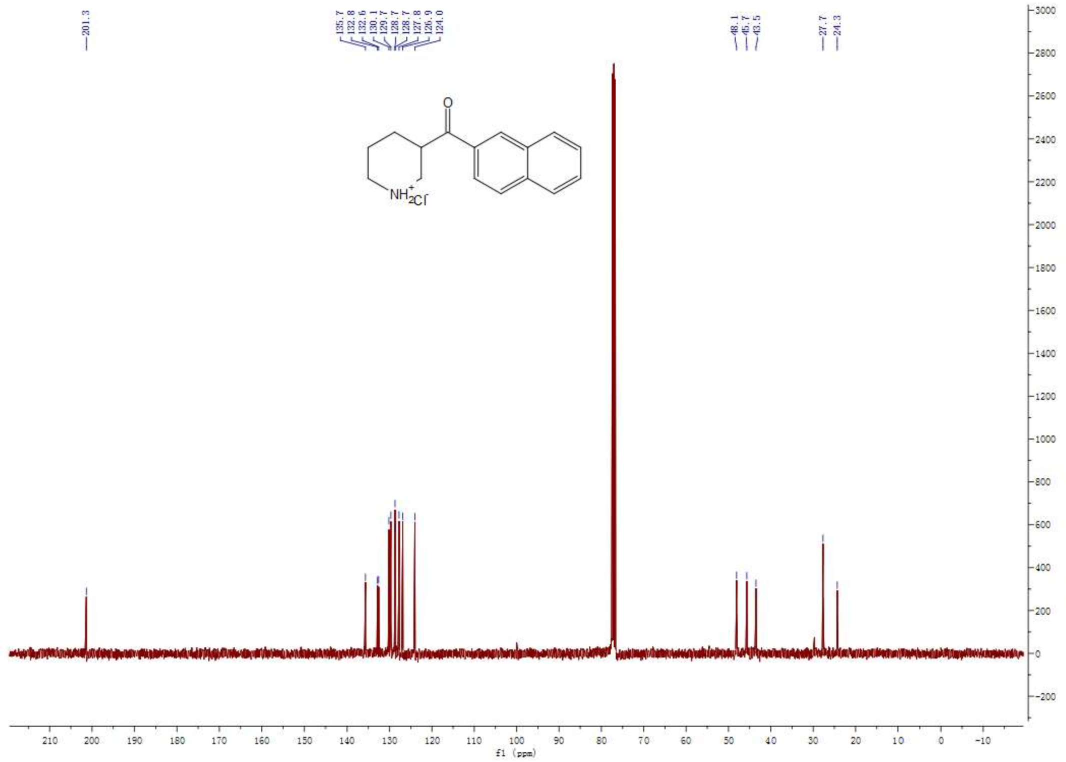

The NMR spectra of 2f

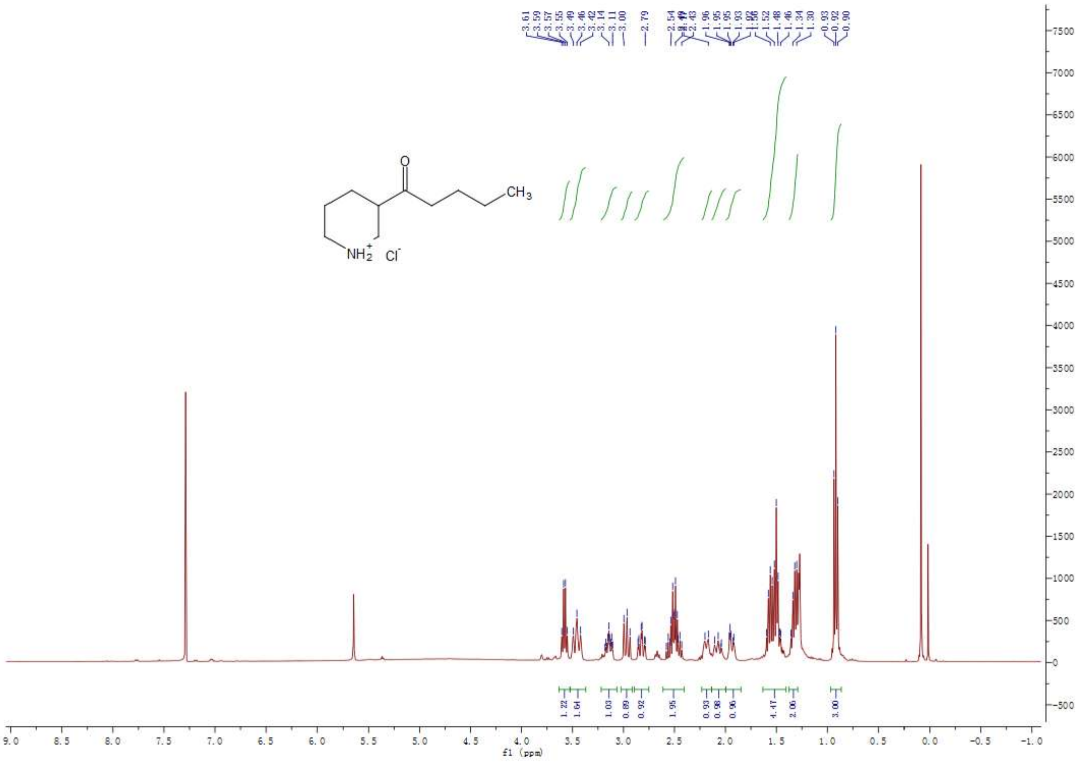

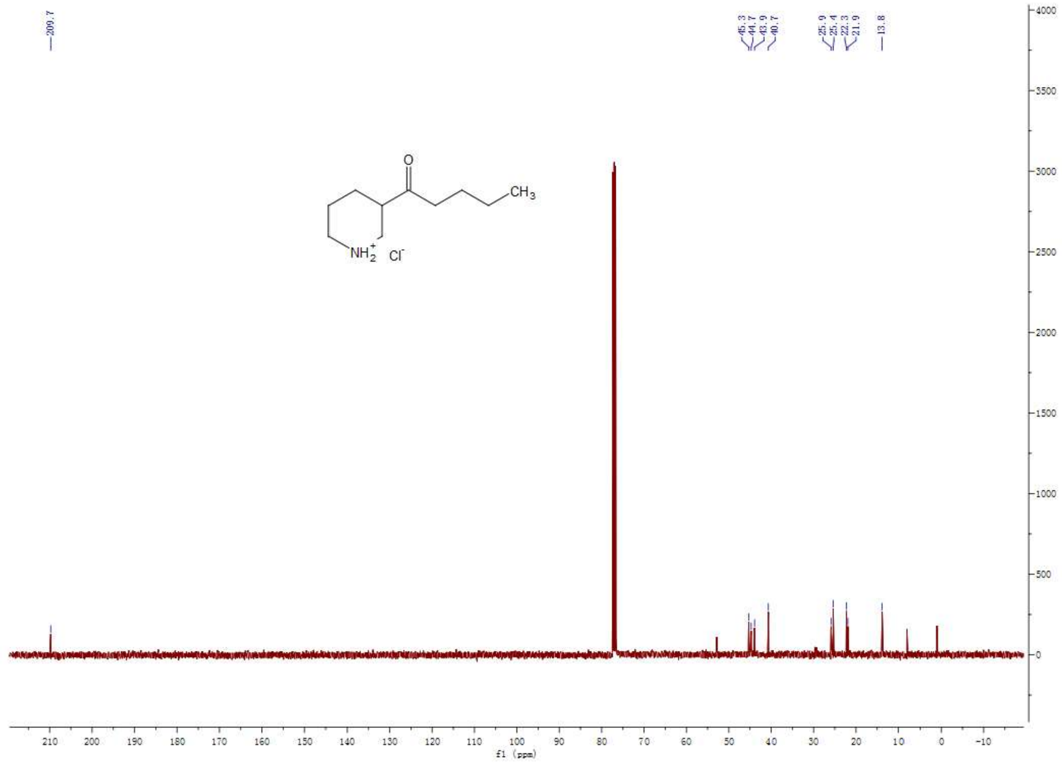

The NMR spectra of 2g

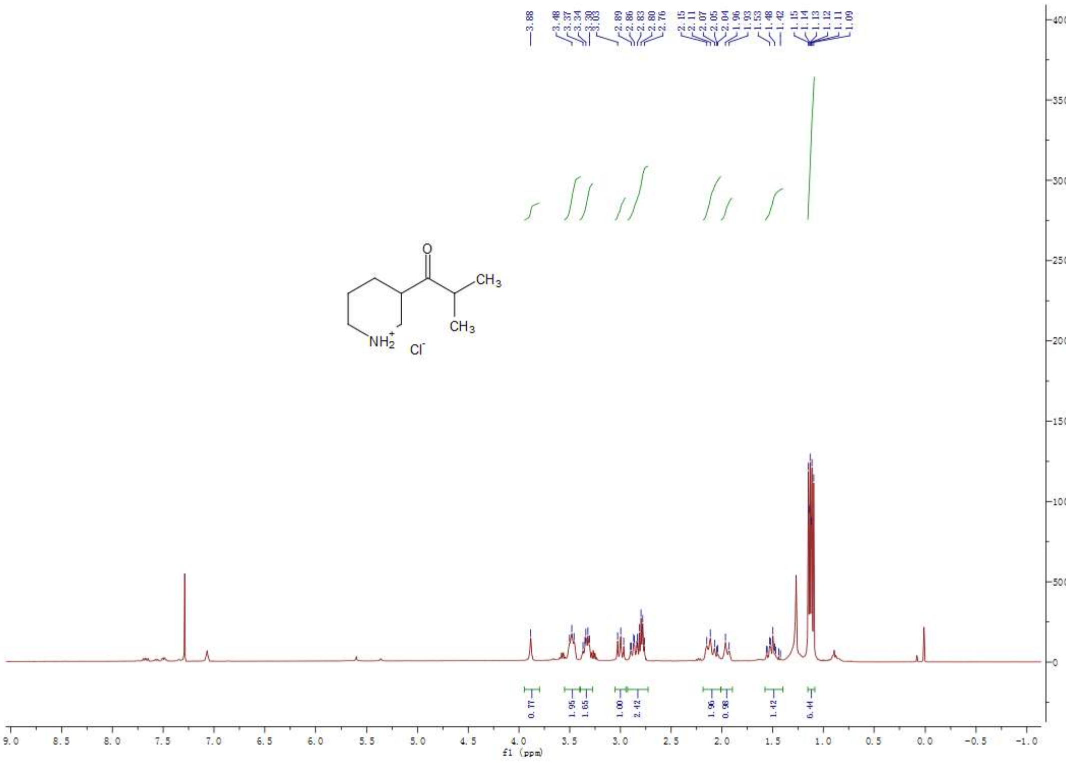

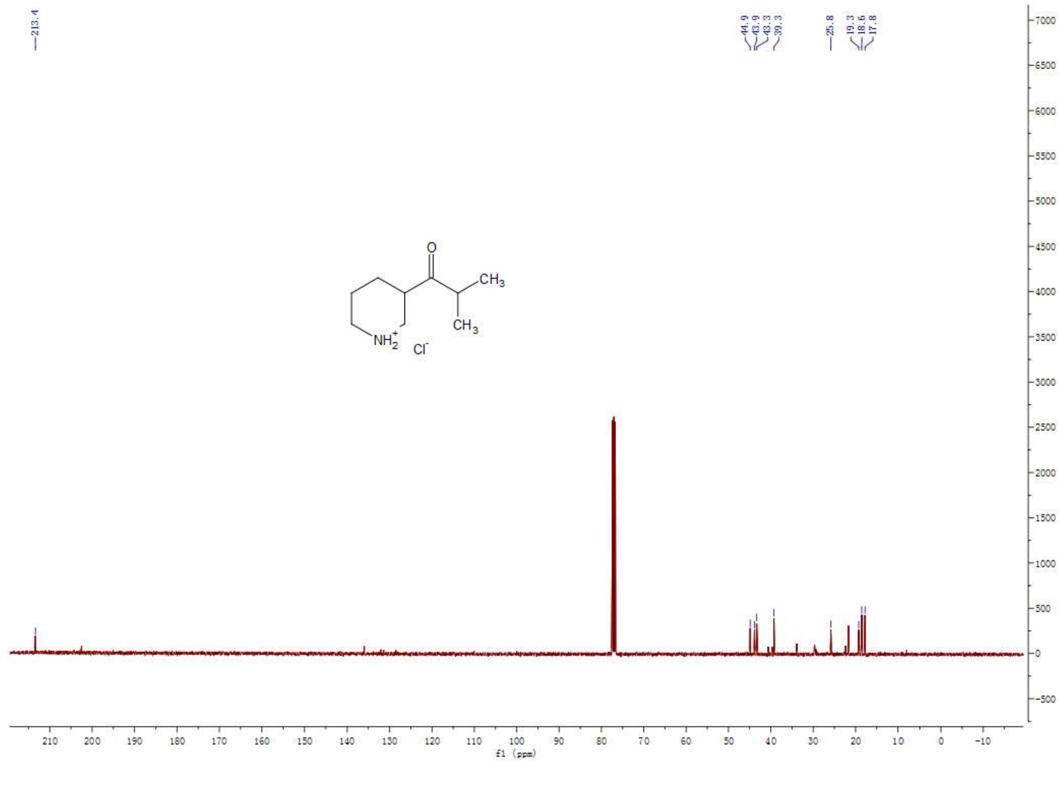

The NMR spectra of 2h

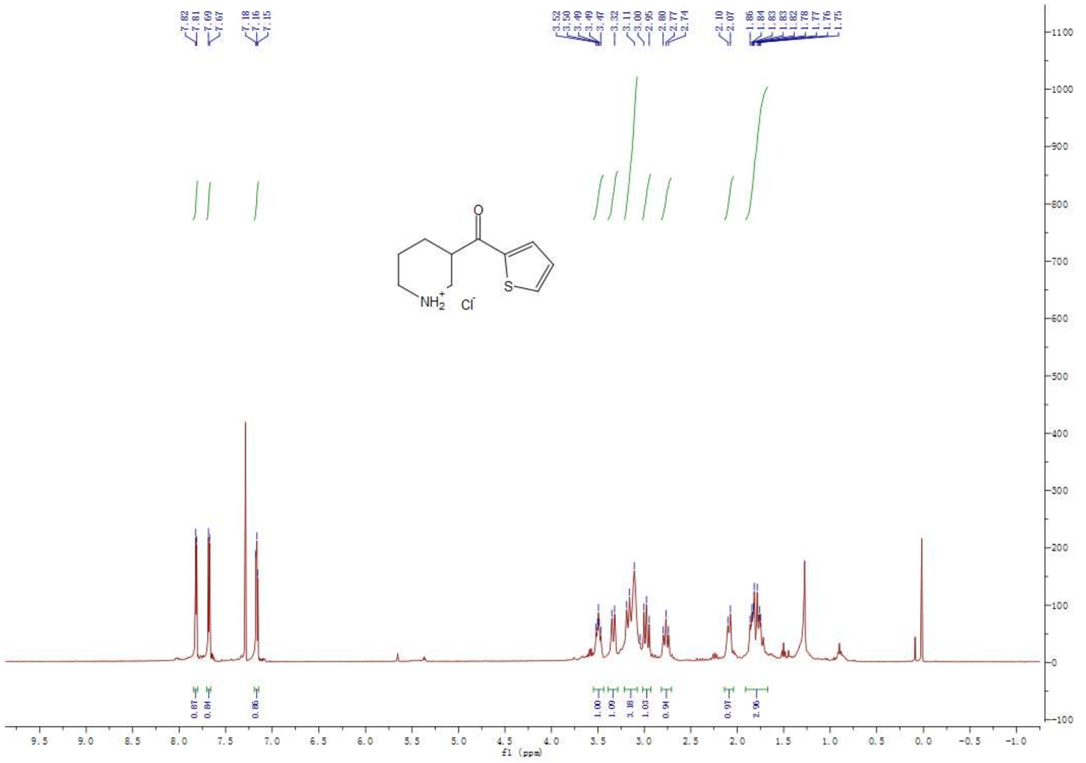

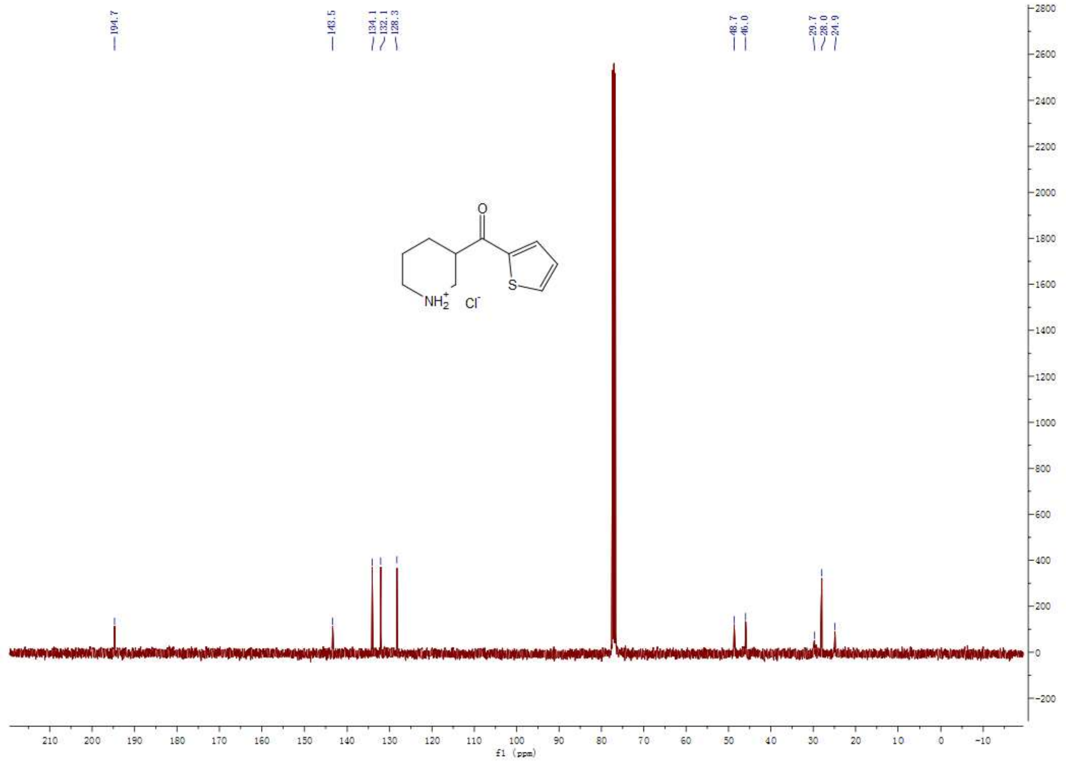

The NMR spectra of 2i

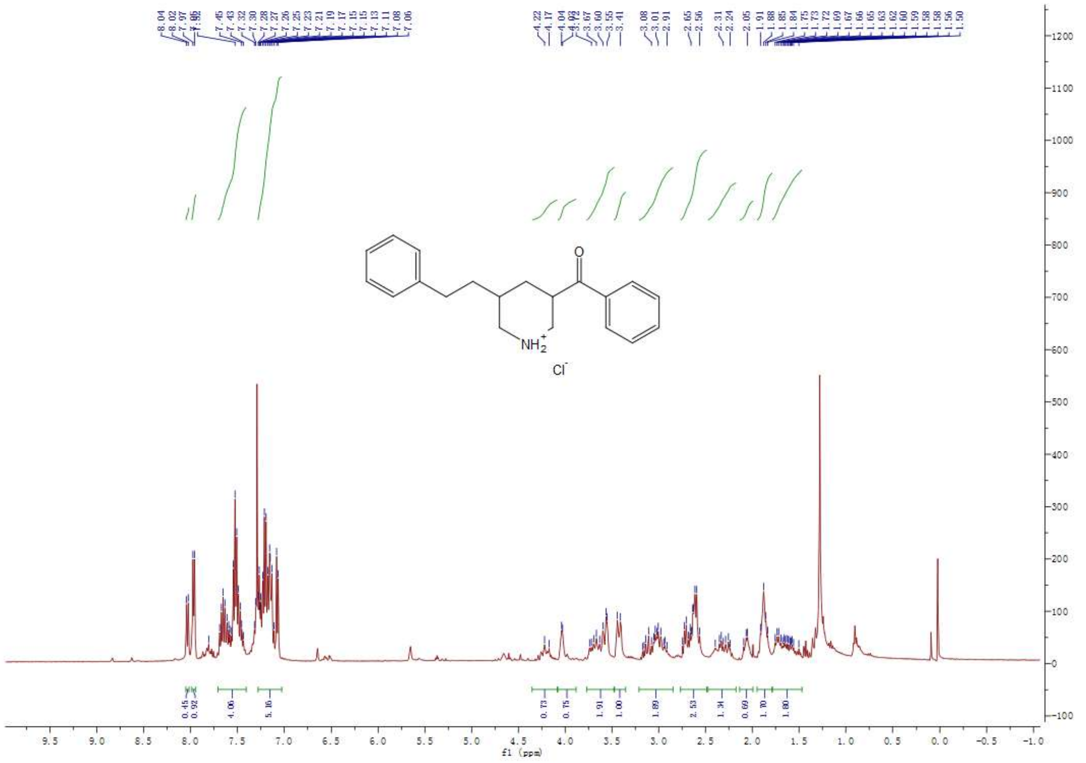

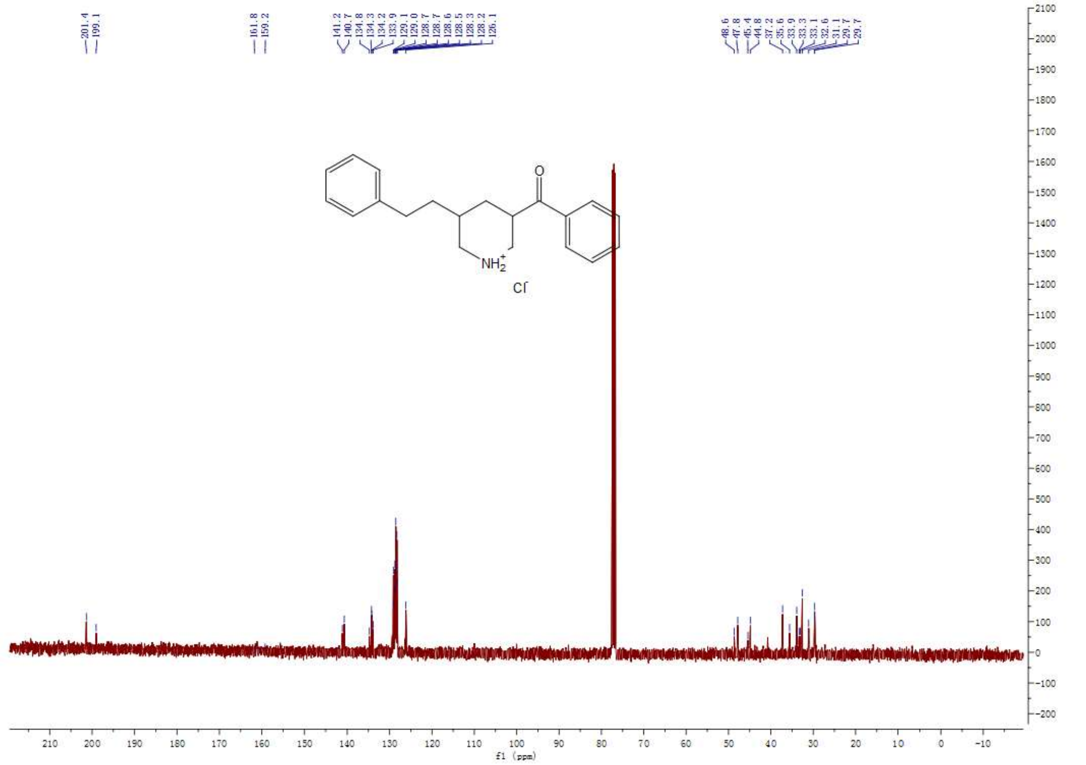

The NMR spectra of 2j

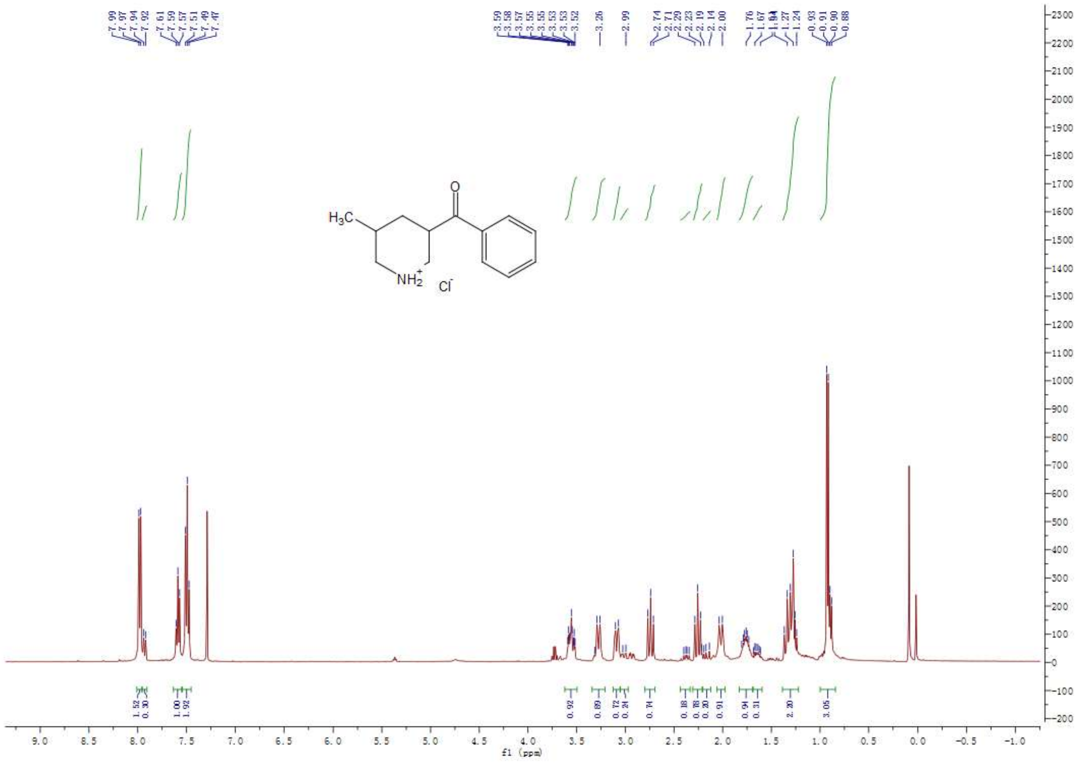

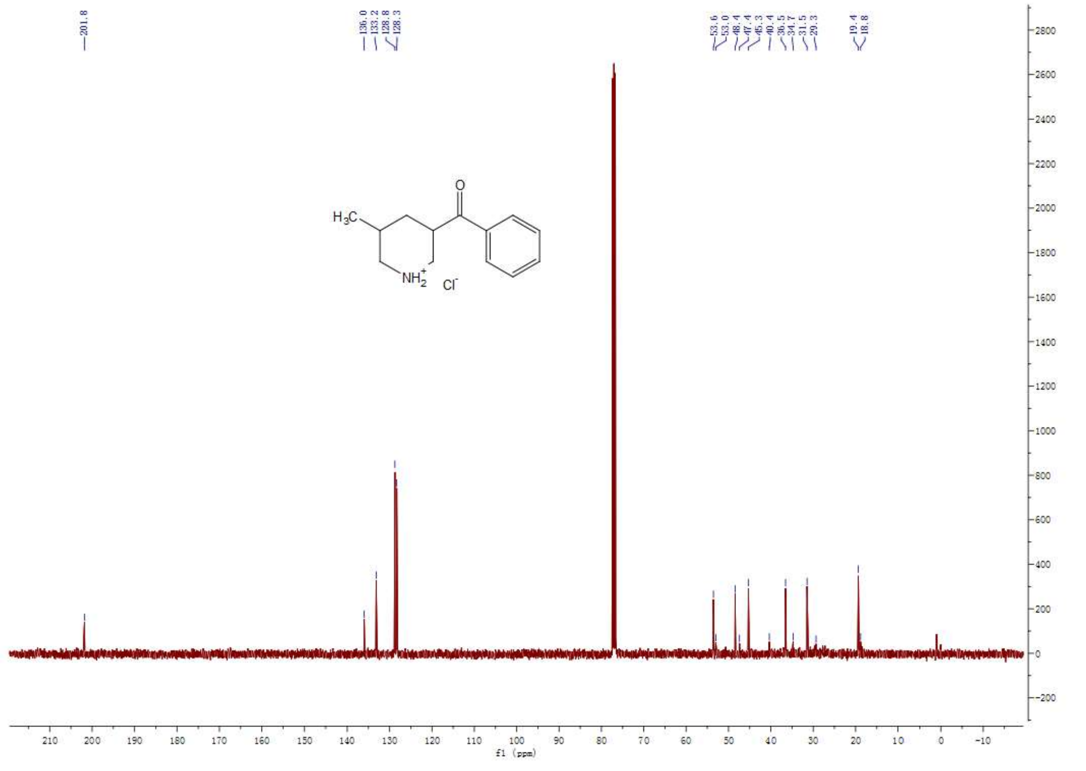

The NMR spectra of 2k

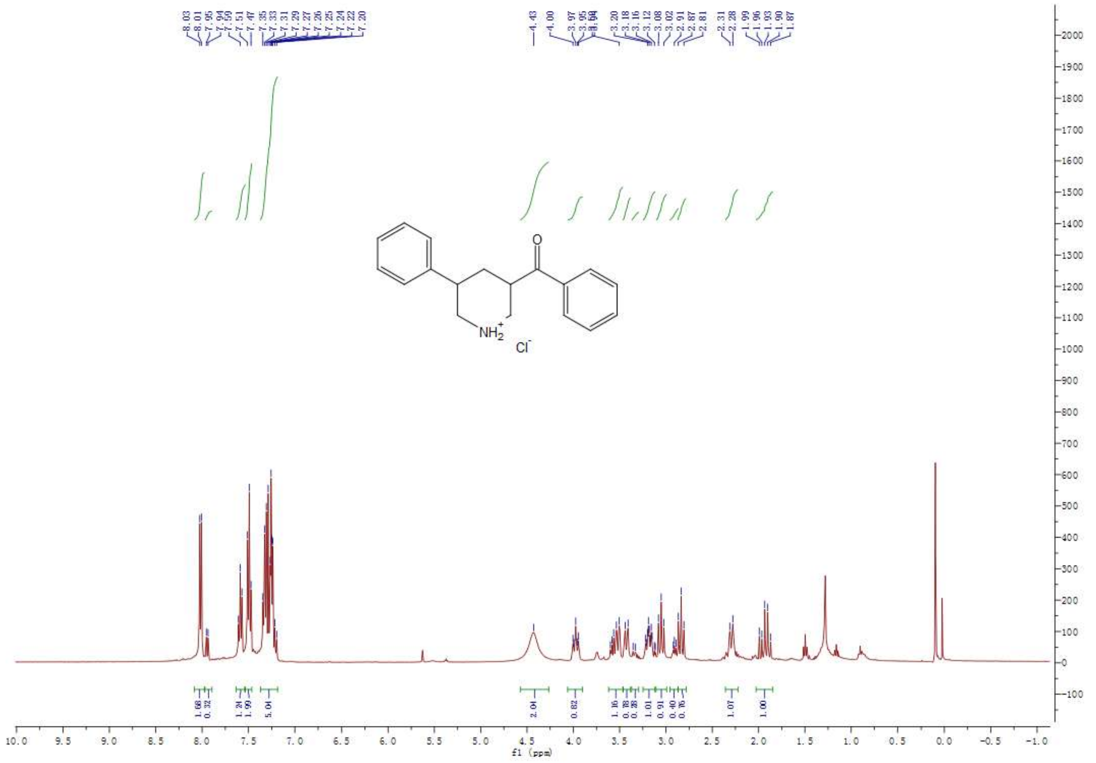

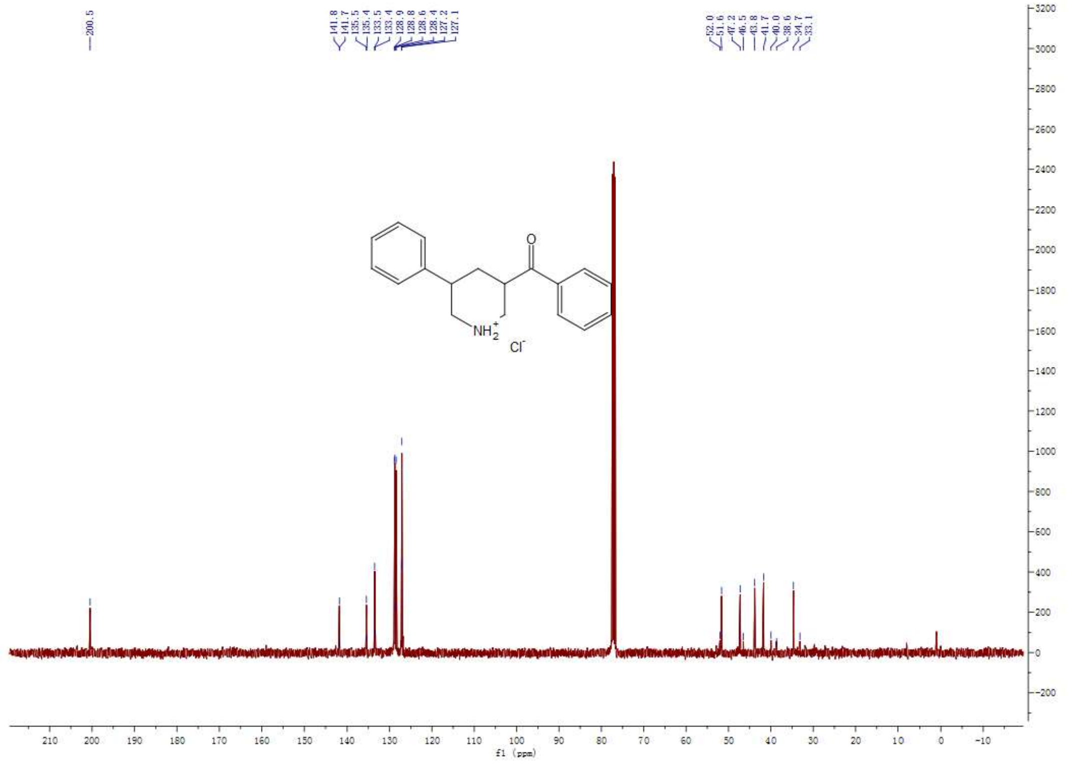

The NMR spectra of 2l

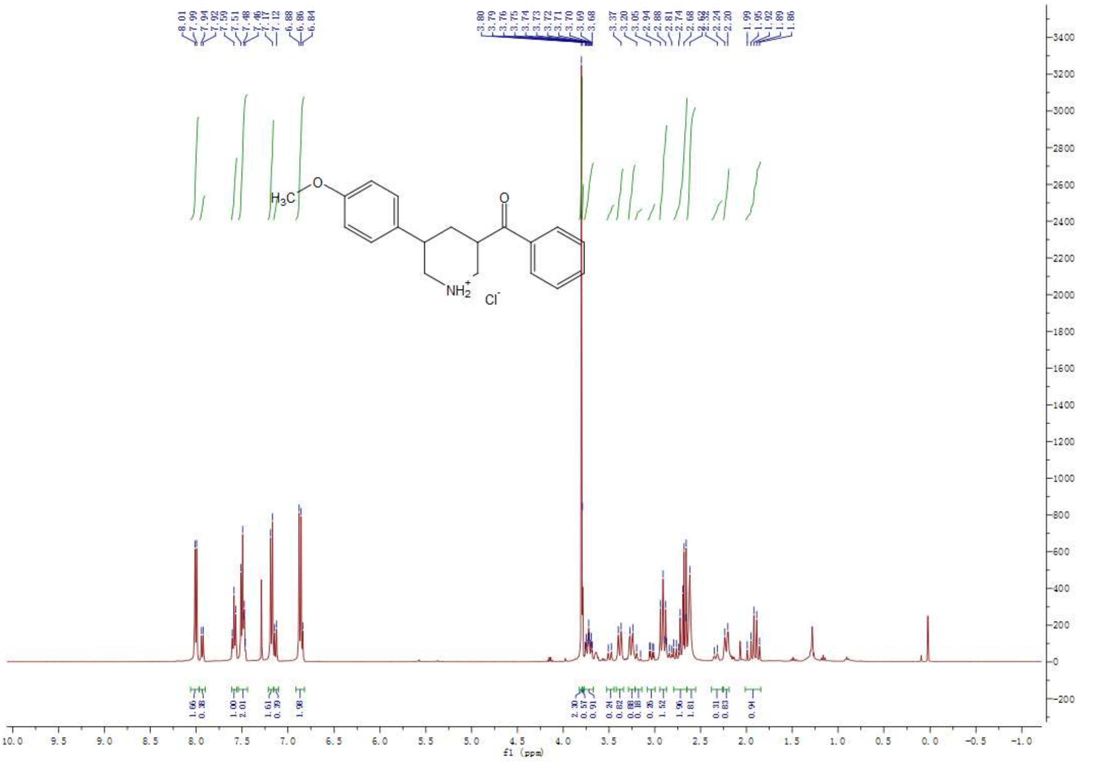

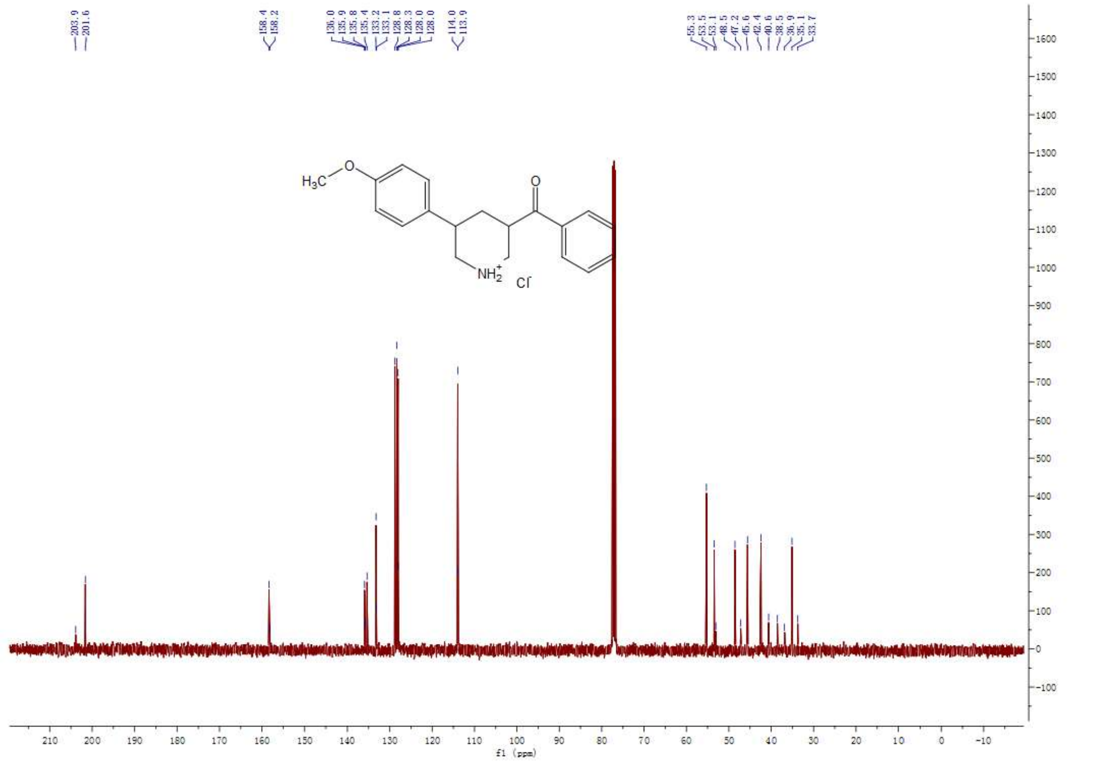

The NMR spectra of 2m

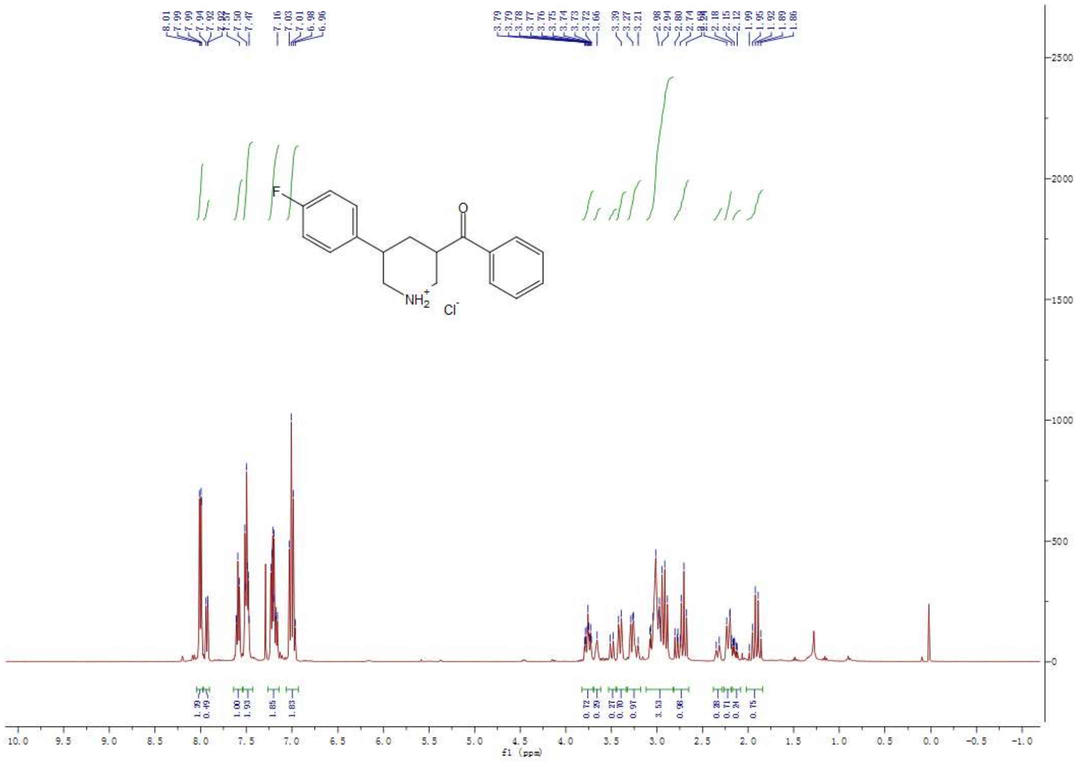

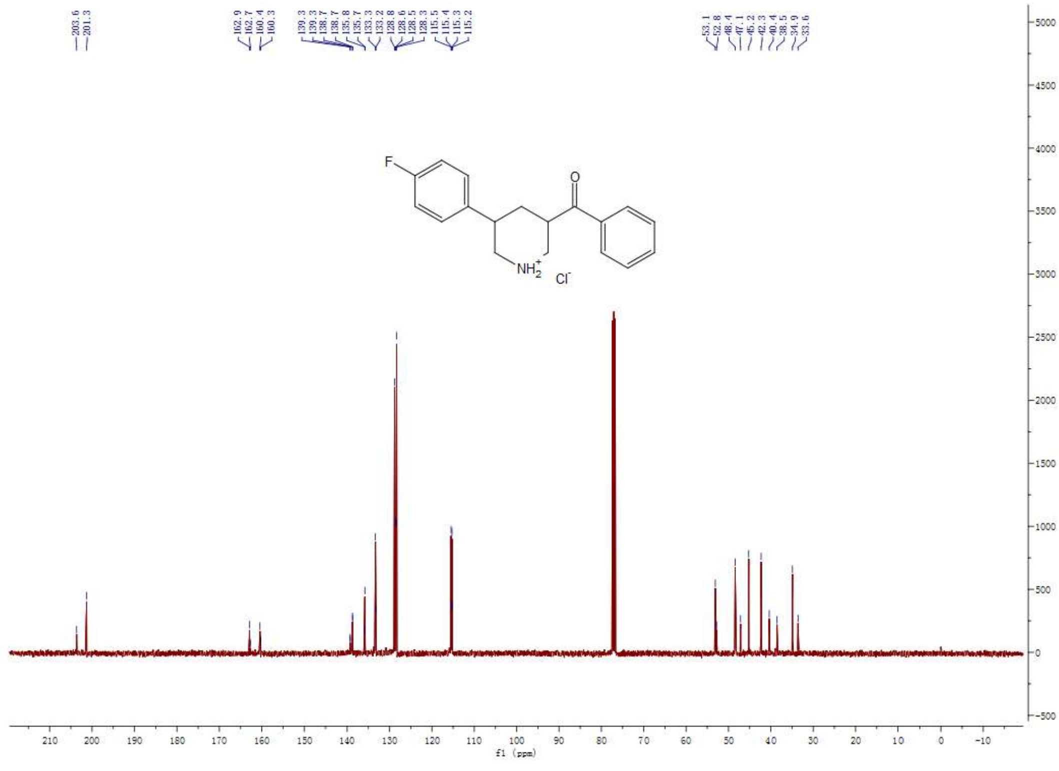

The NMR spectra of 2n

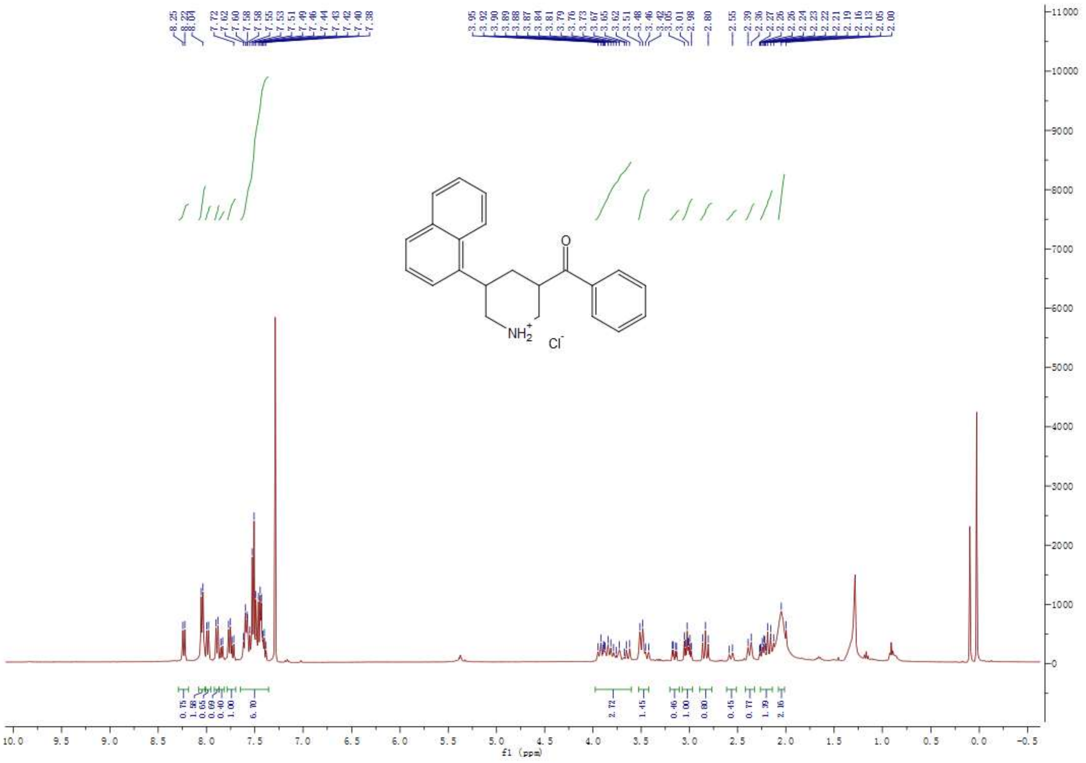

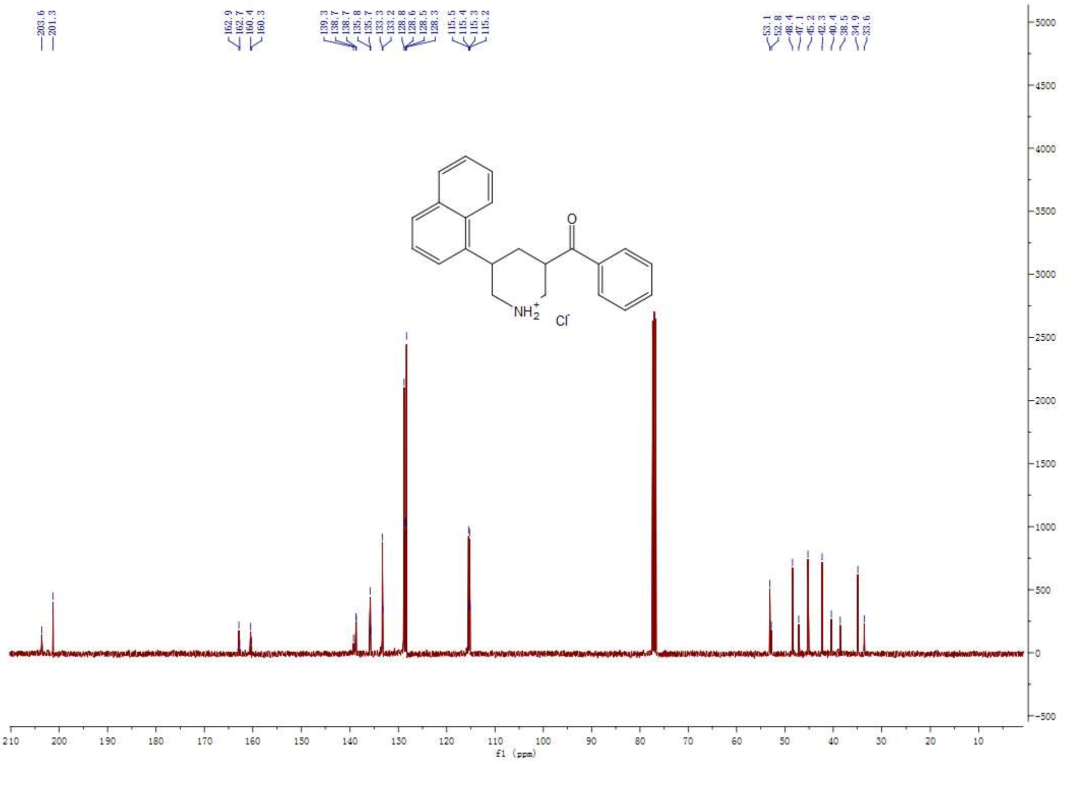

The NMR spectra of 2o

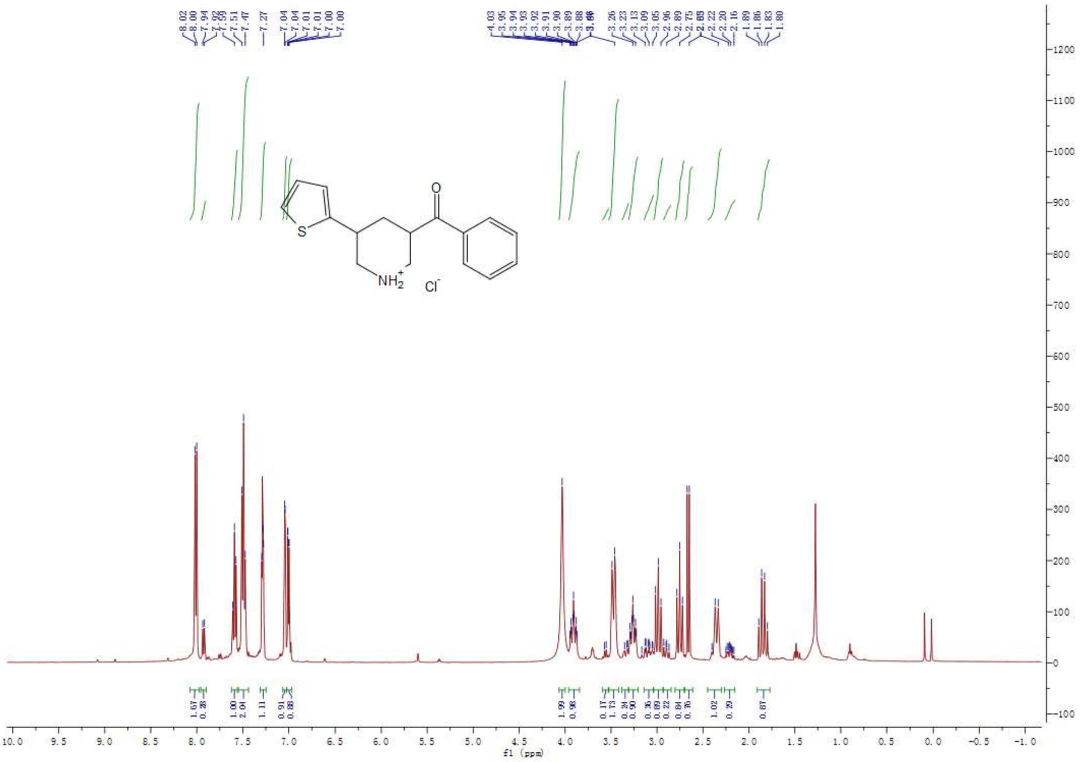

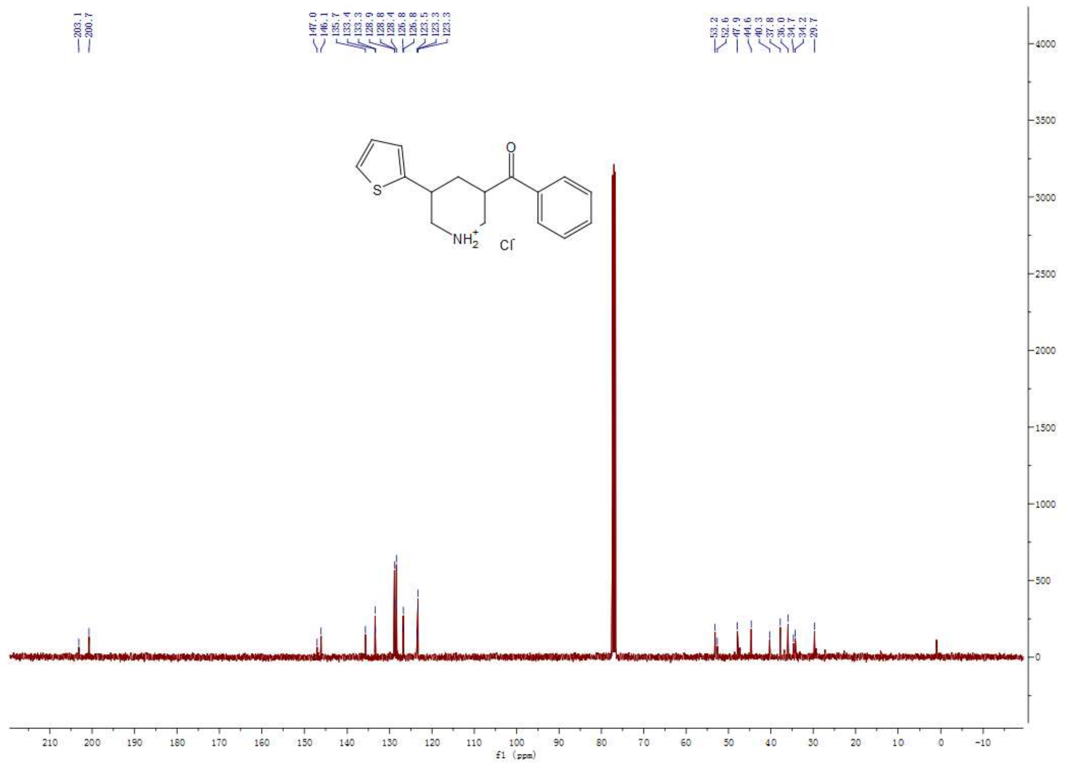

The NMR spectra of 2p

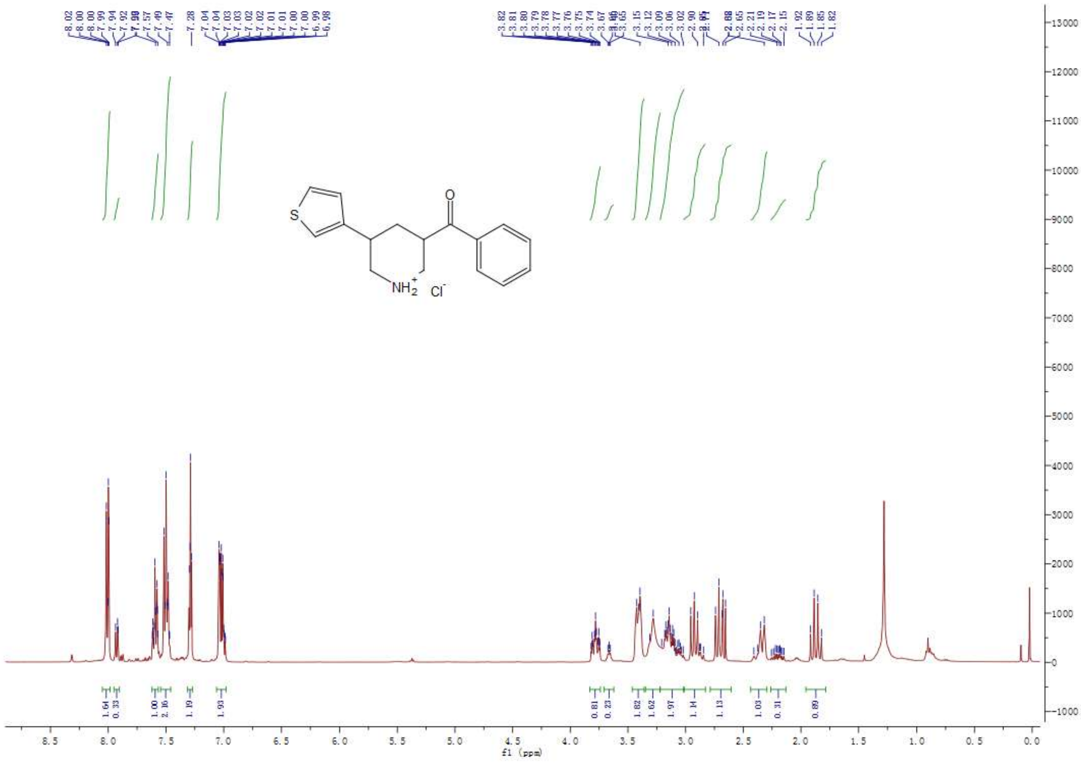

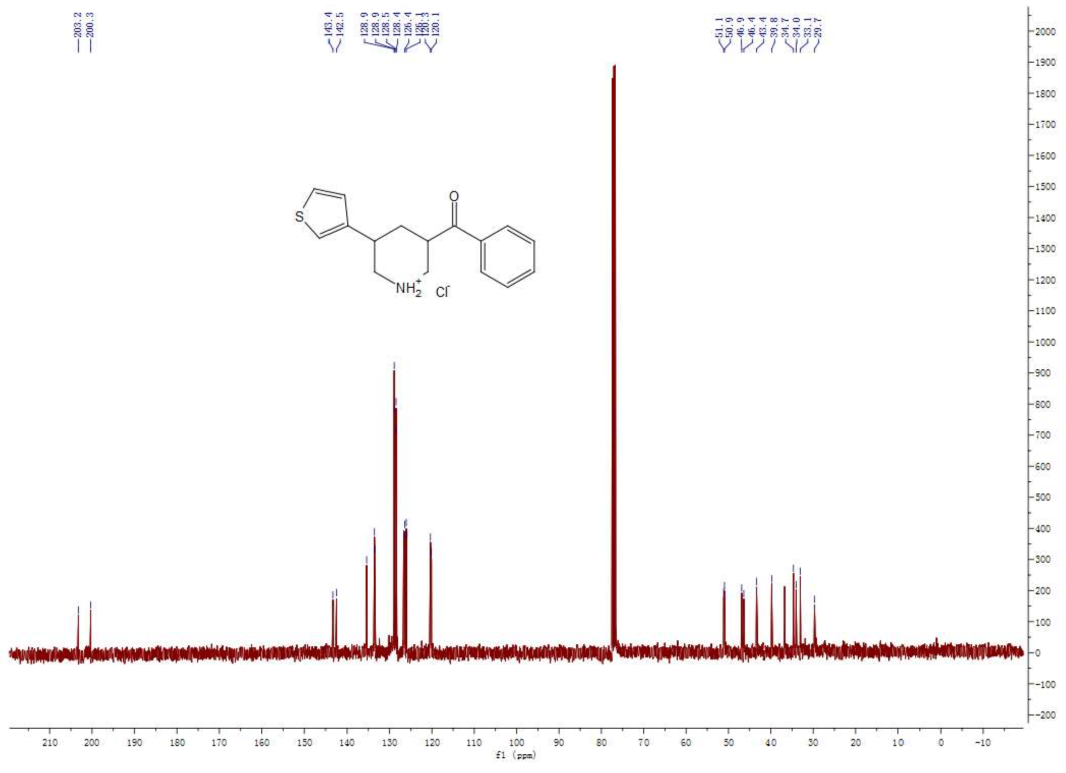

The NMR spectra of 2q

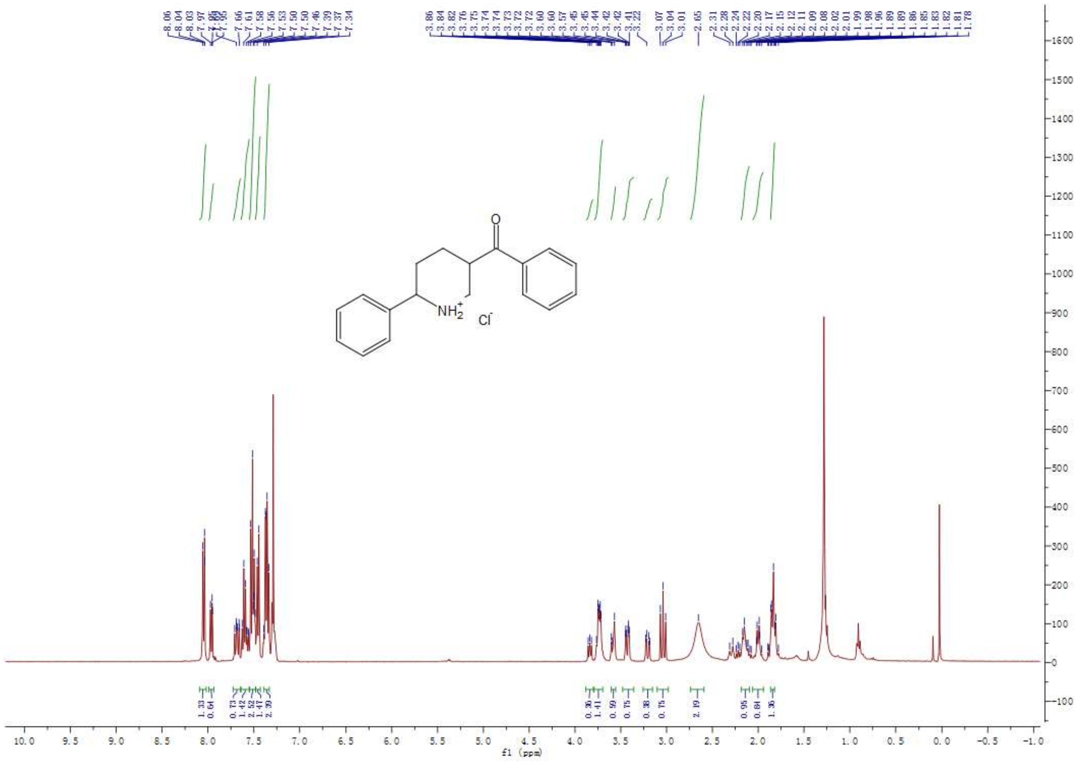

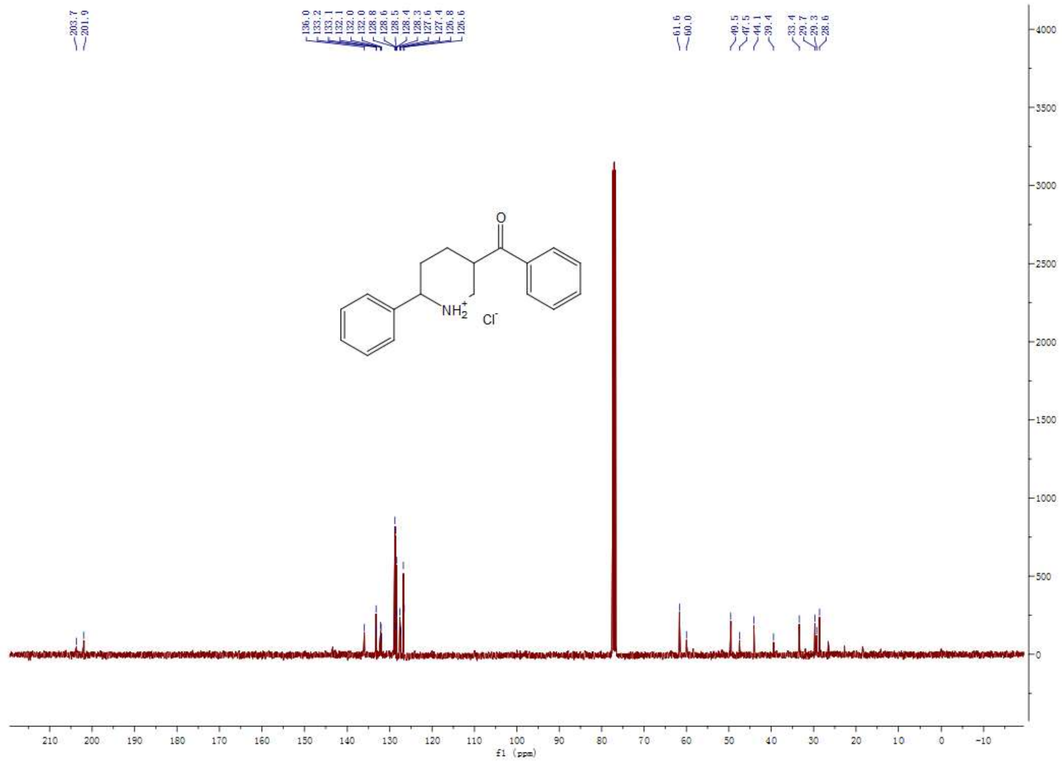

The NMR spectra of 2r

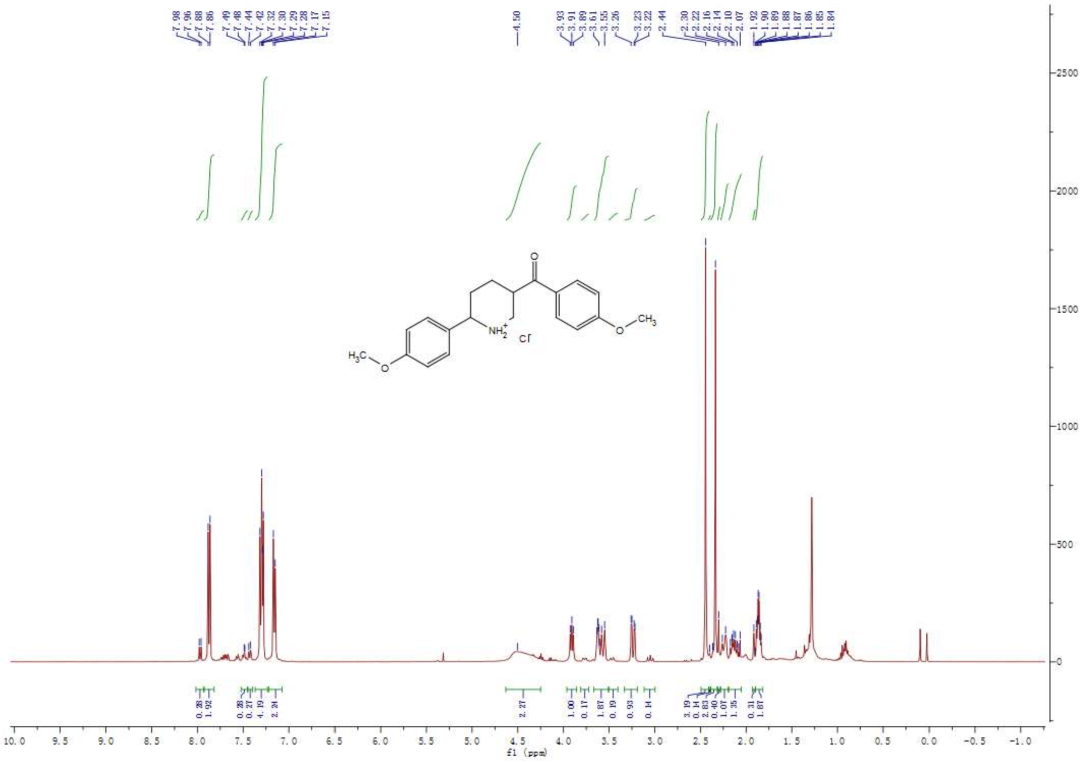

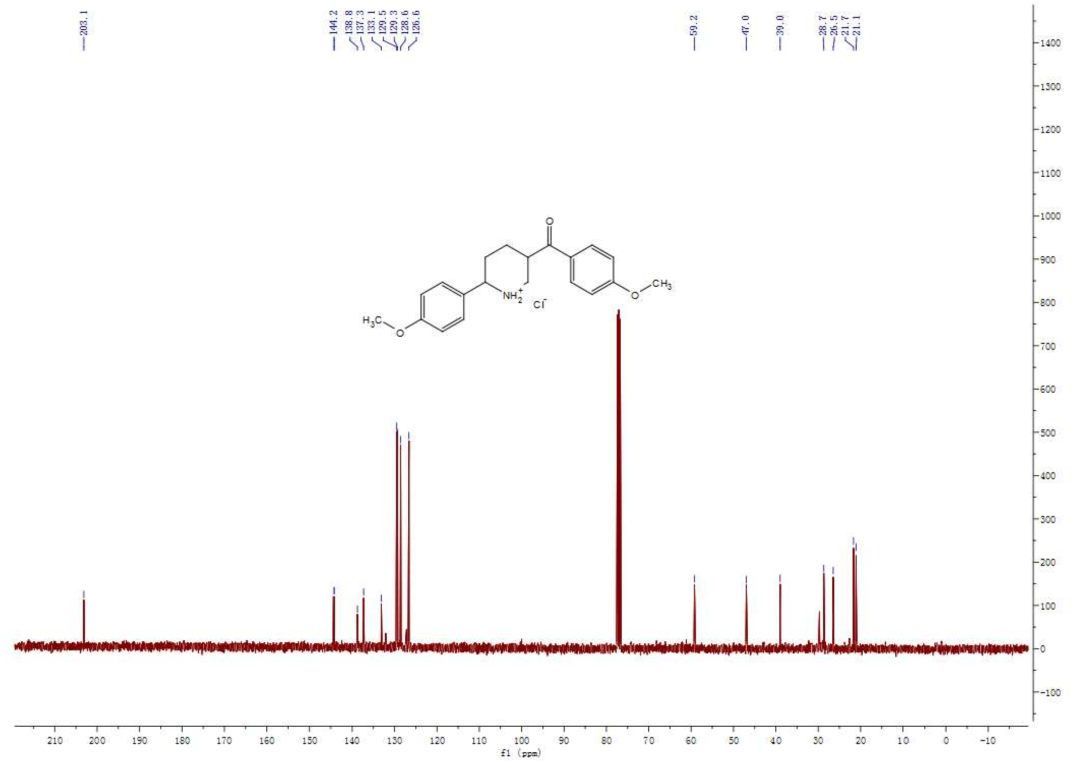

The NMR spectra of 2s

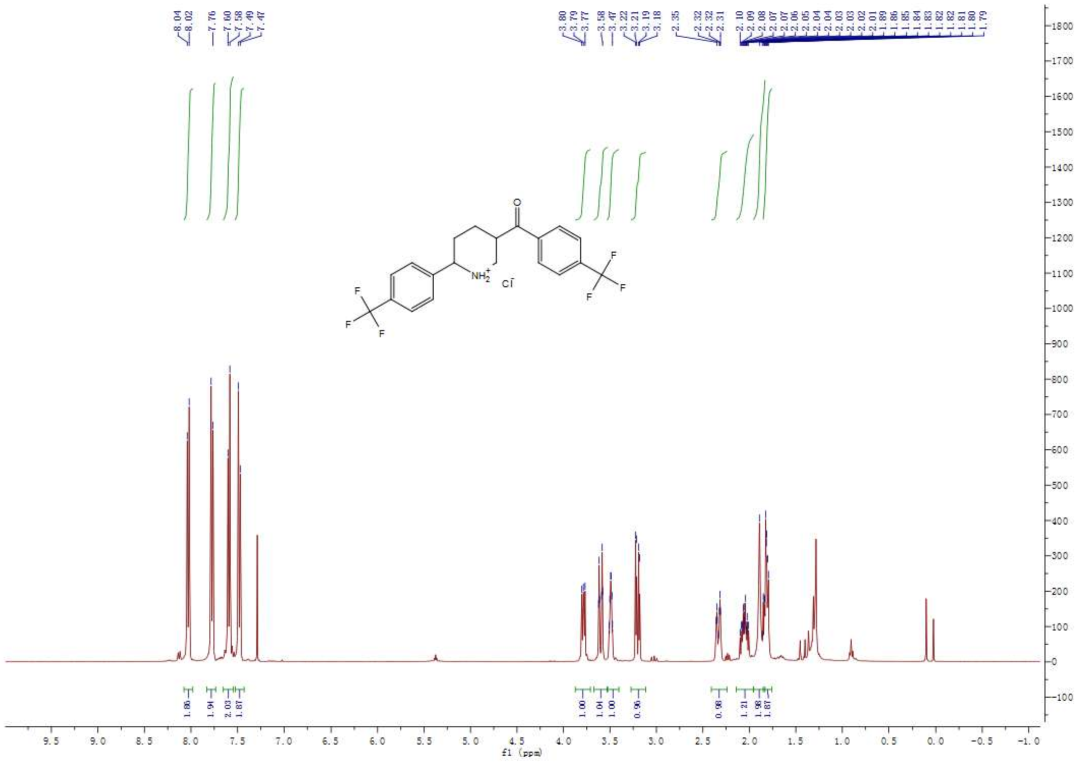

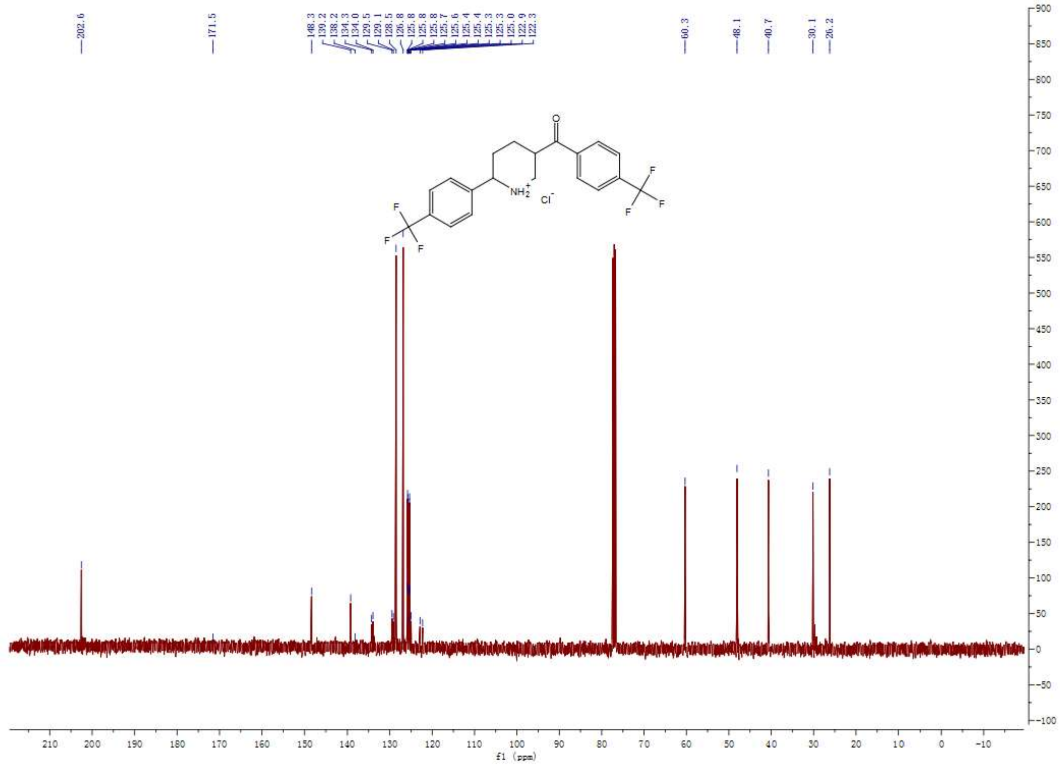

The NMR spectra of 2t

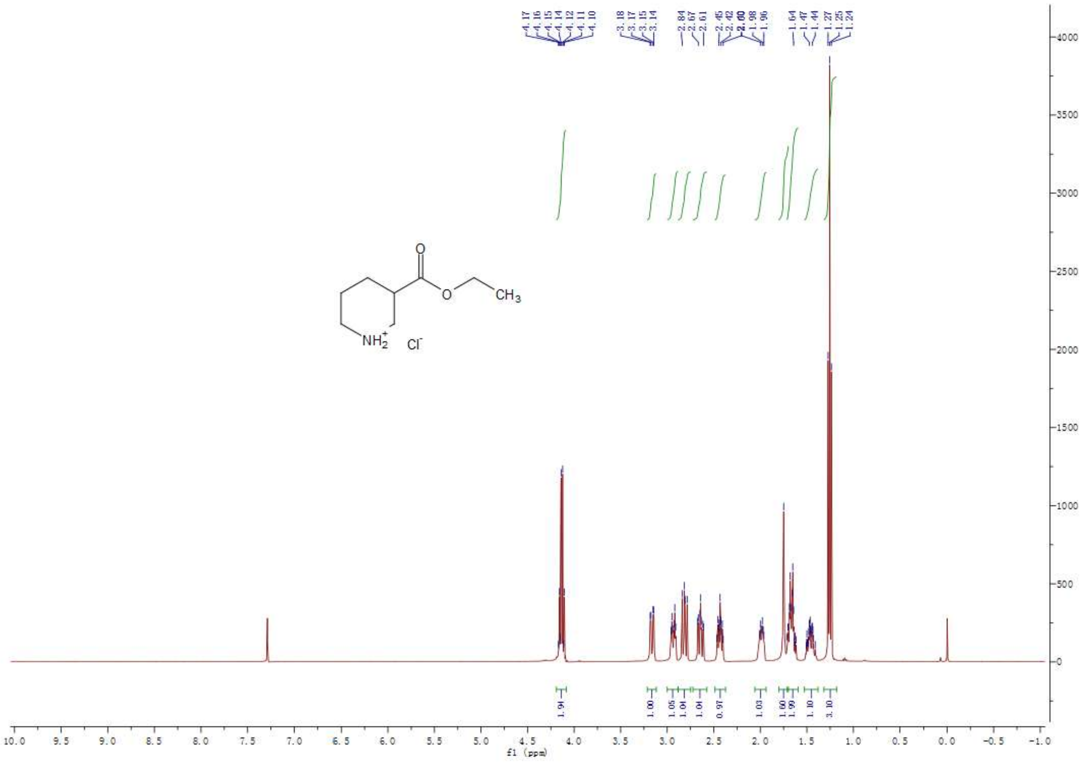

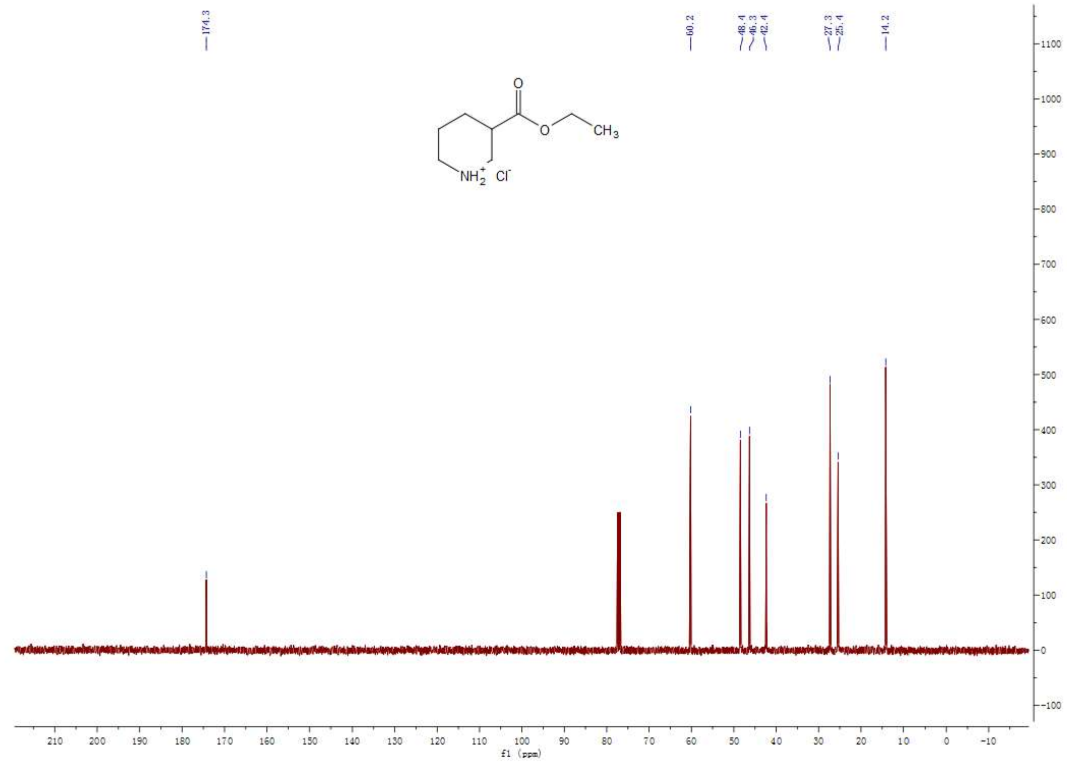

The NMR spectra of 2u

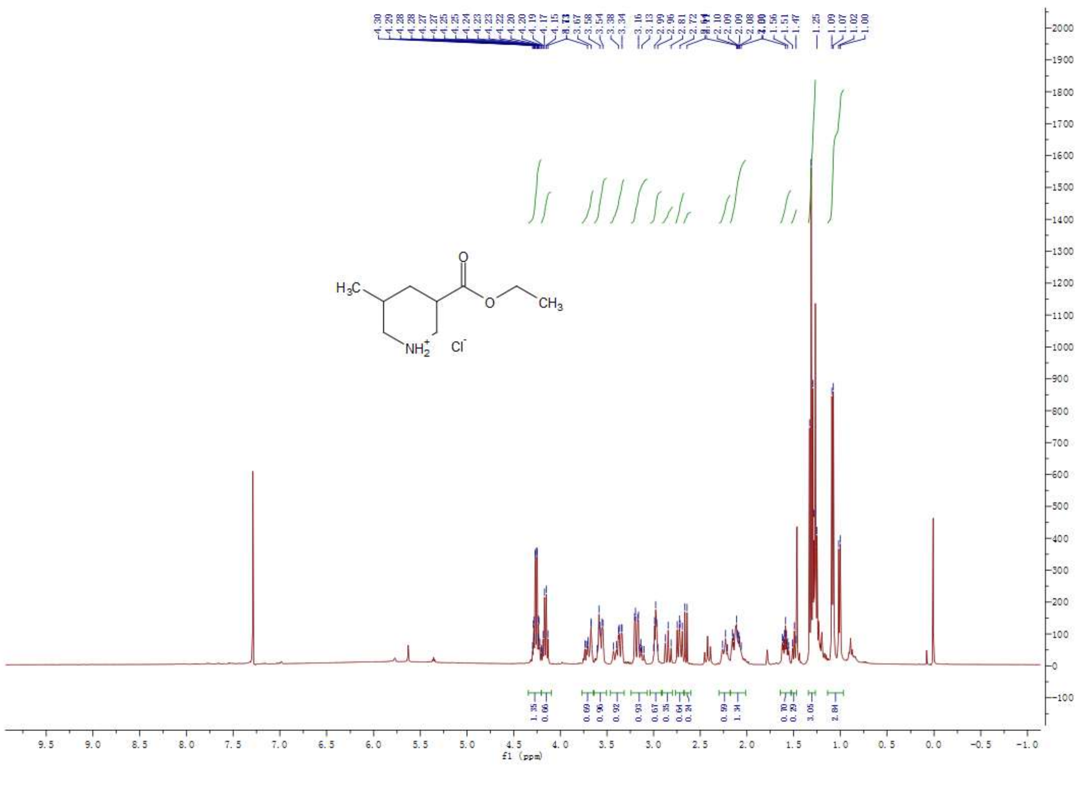

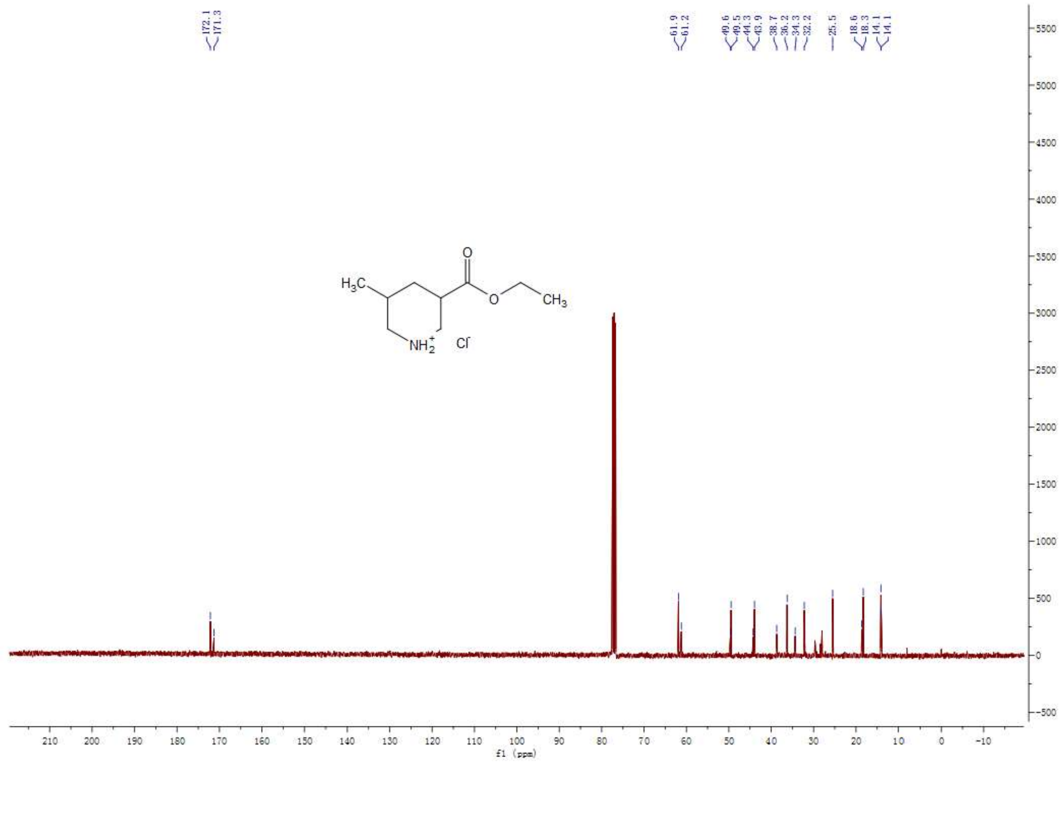

The NMR spectra of 2v

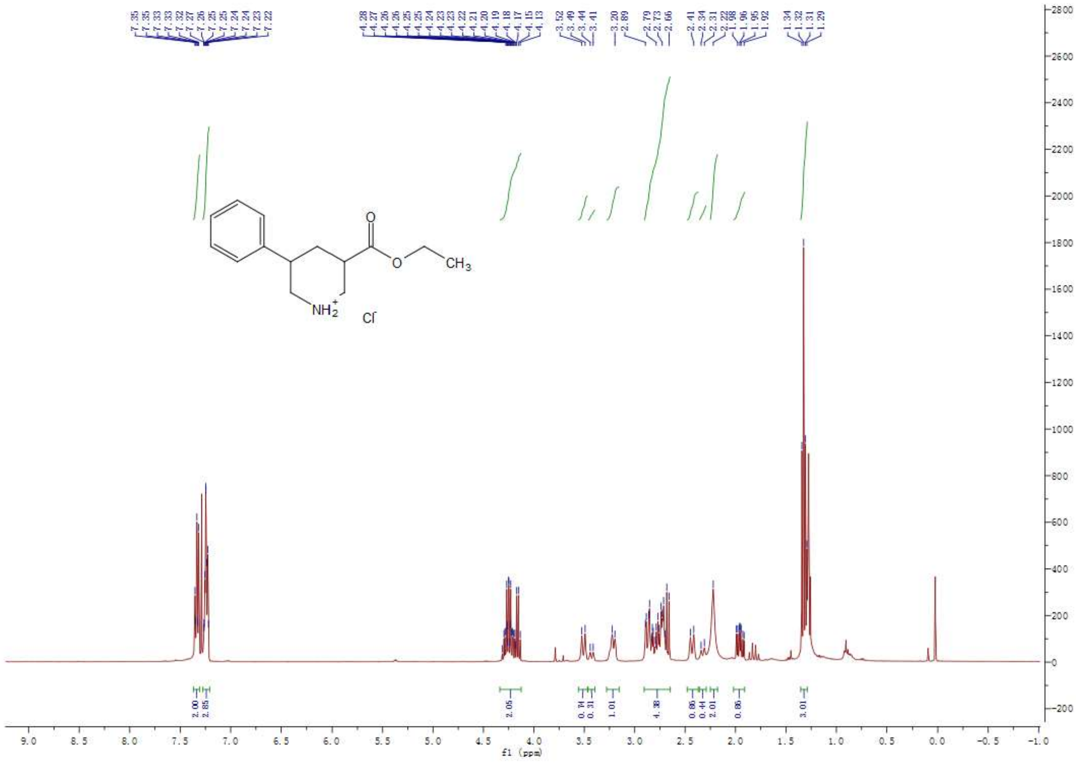

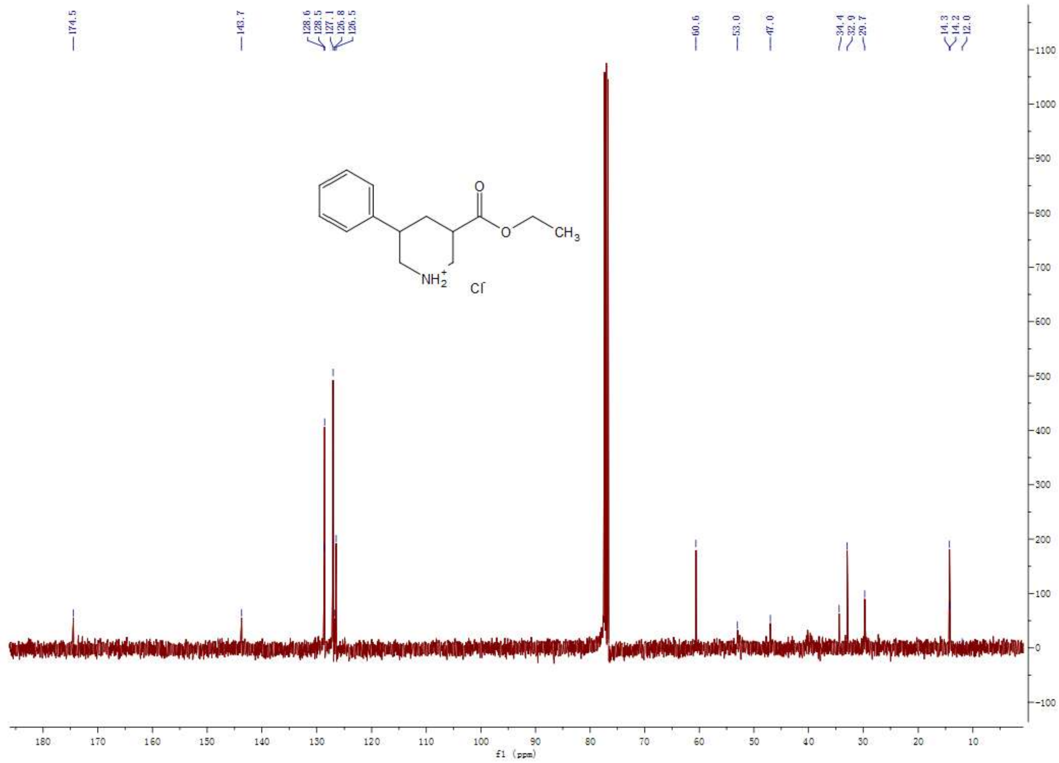

The NMR spectra of 2w

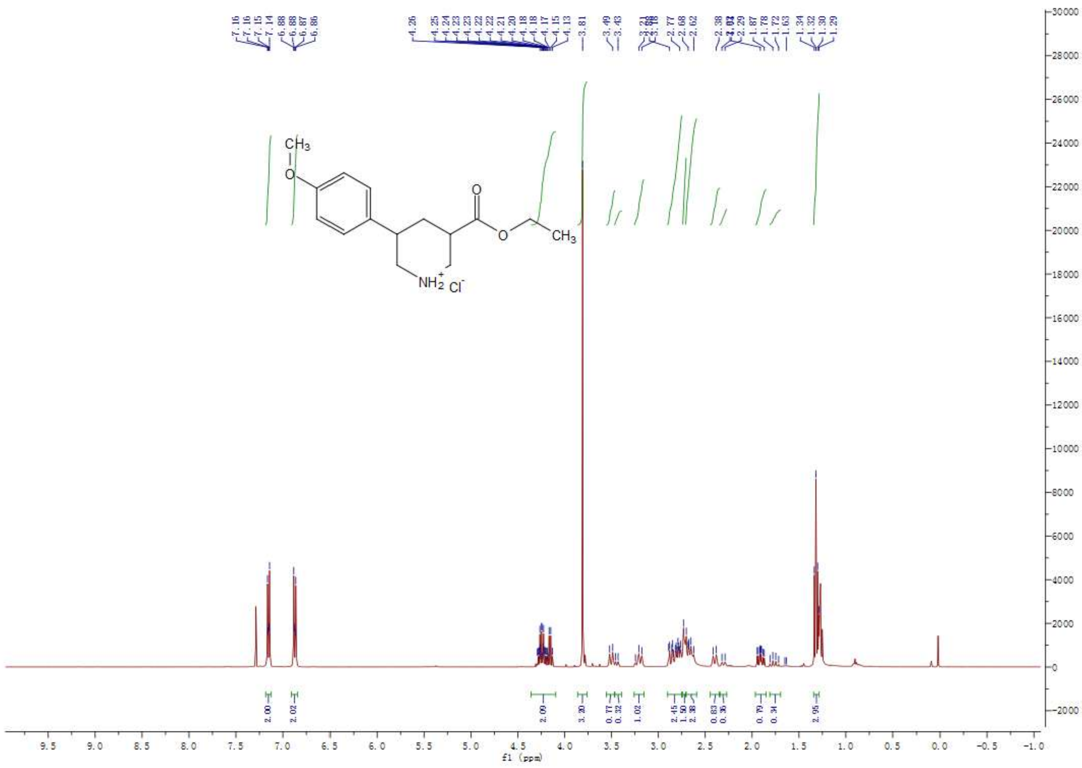

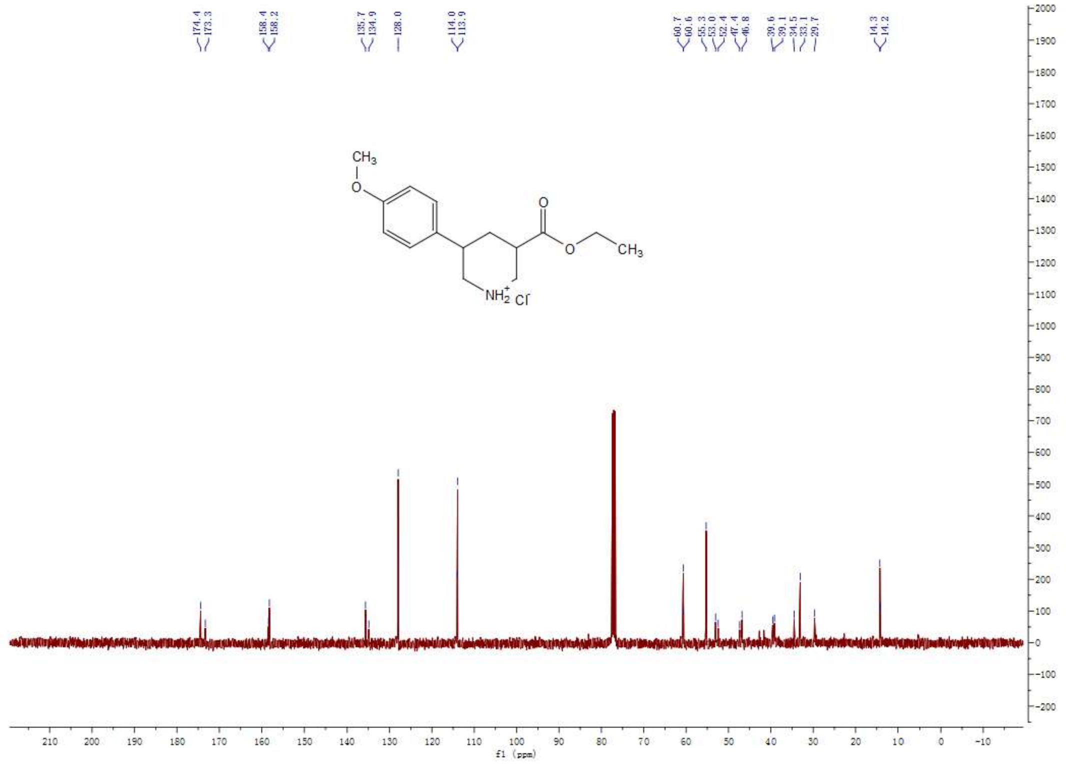

The NMR spectra of 2x

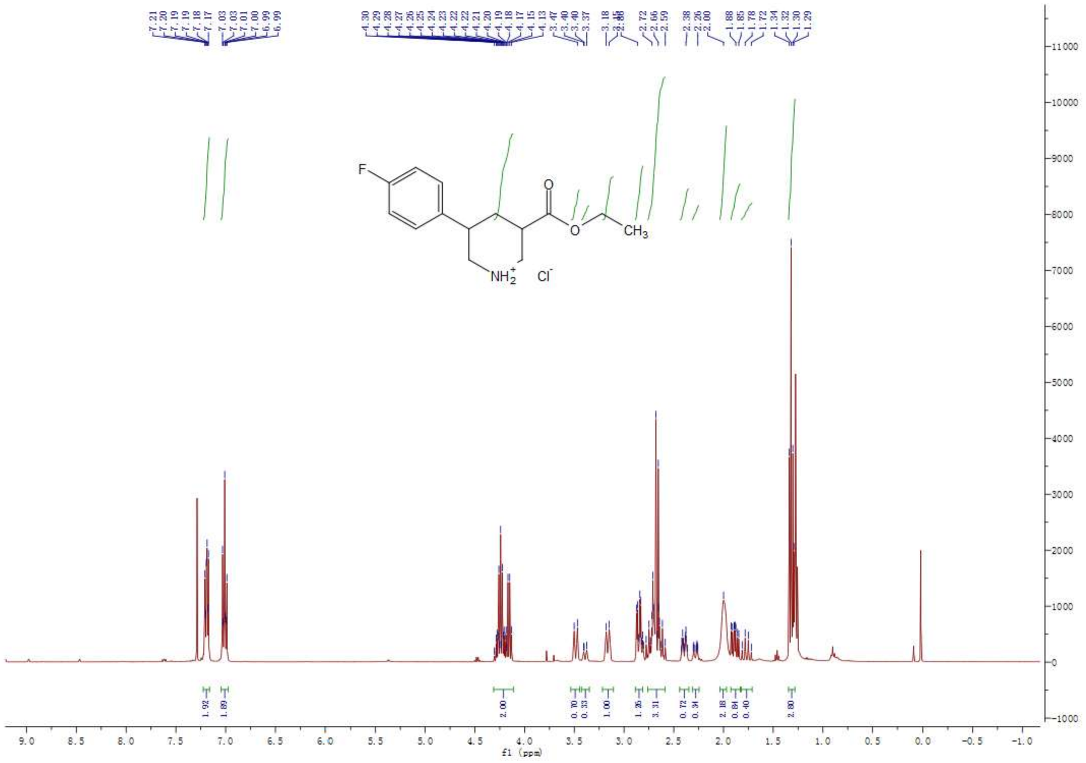

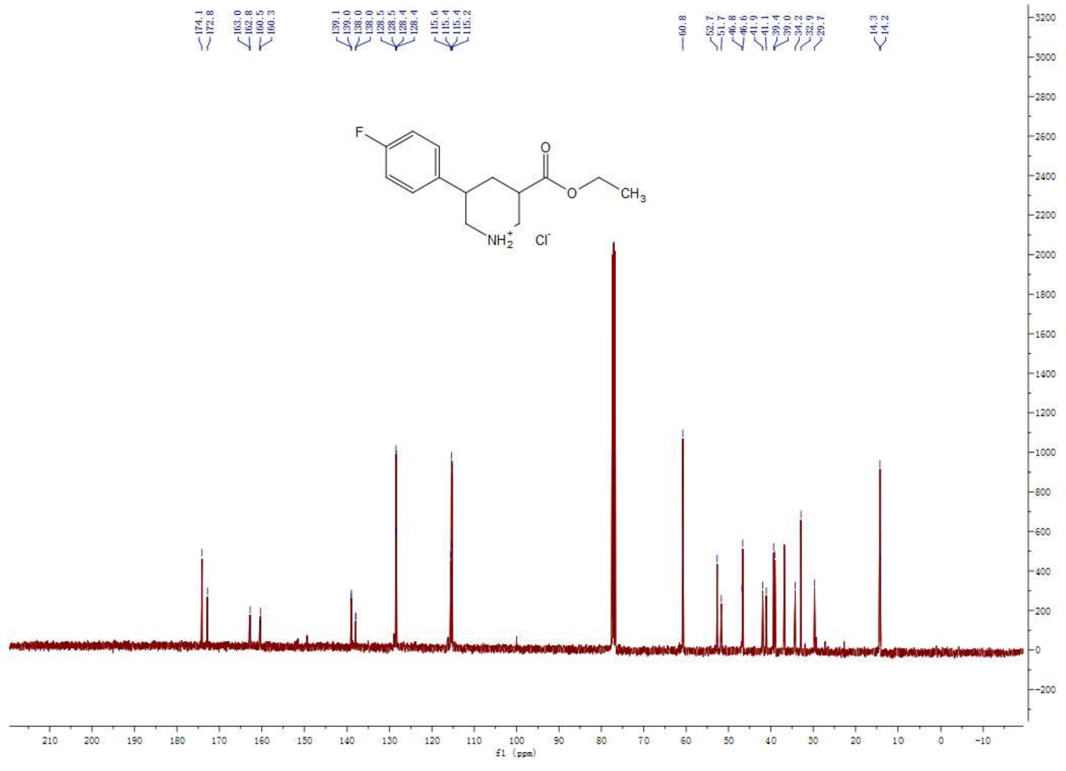

The NMR spectra of 2y

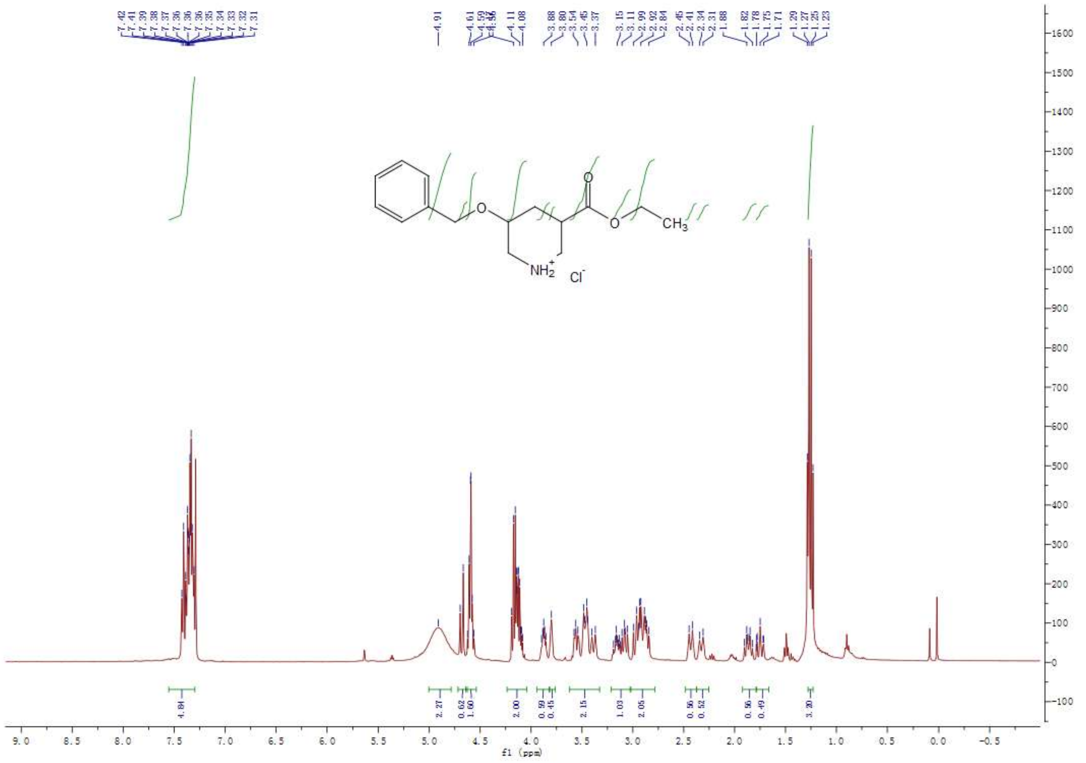

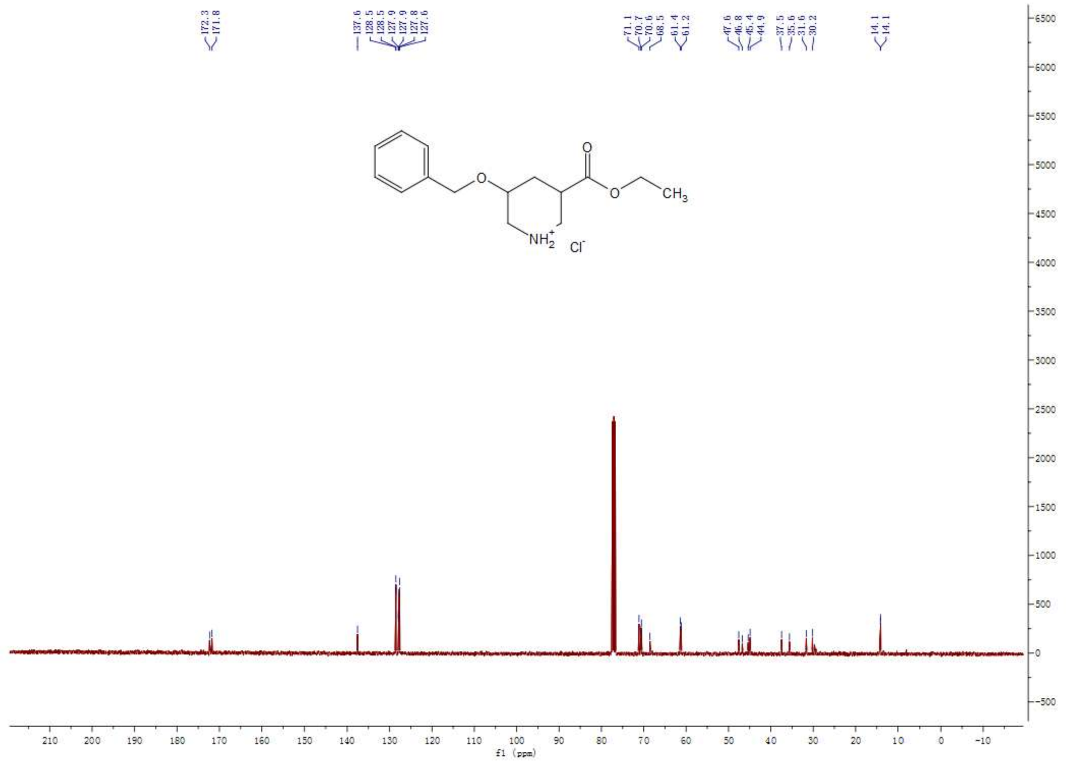

The NMR spectra of 3c

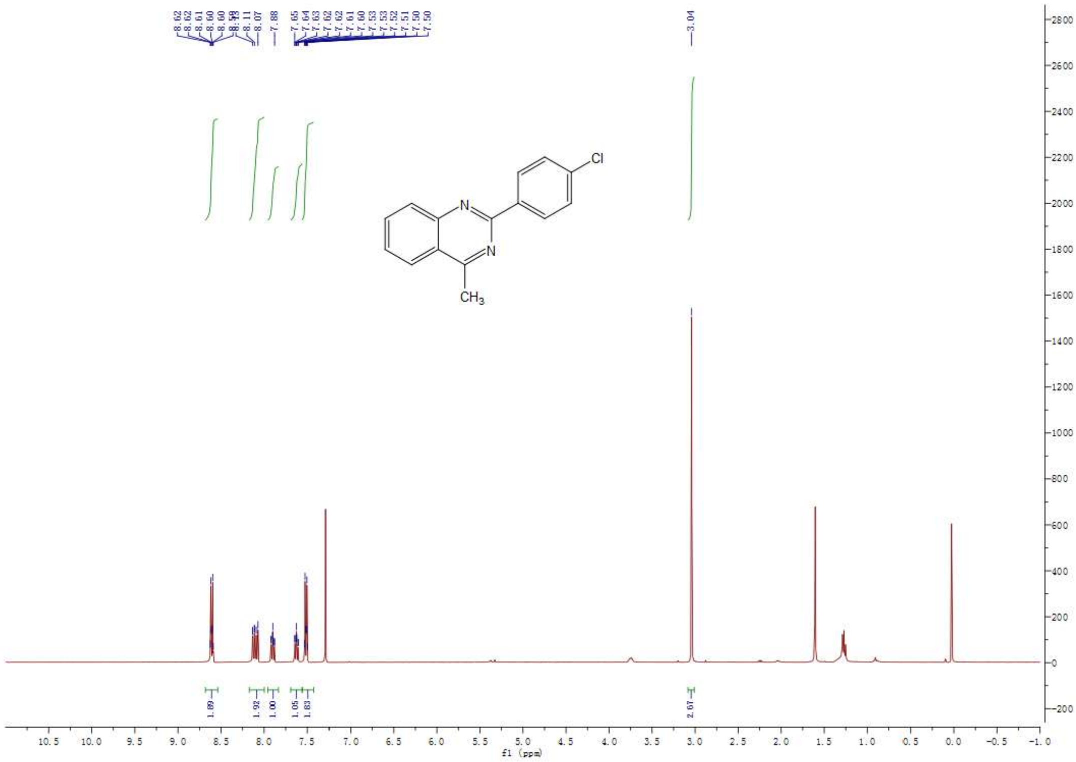

The NMR spectra of 3d

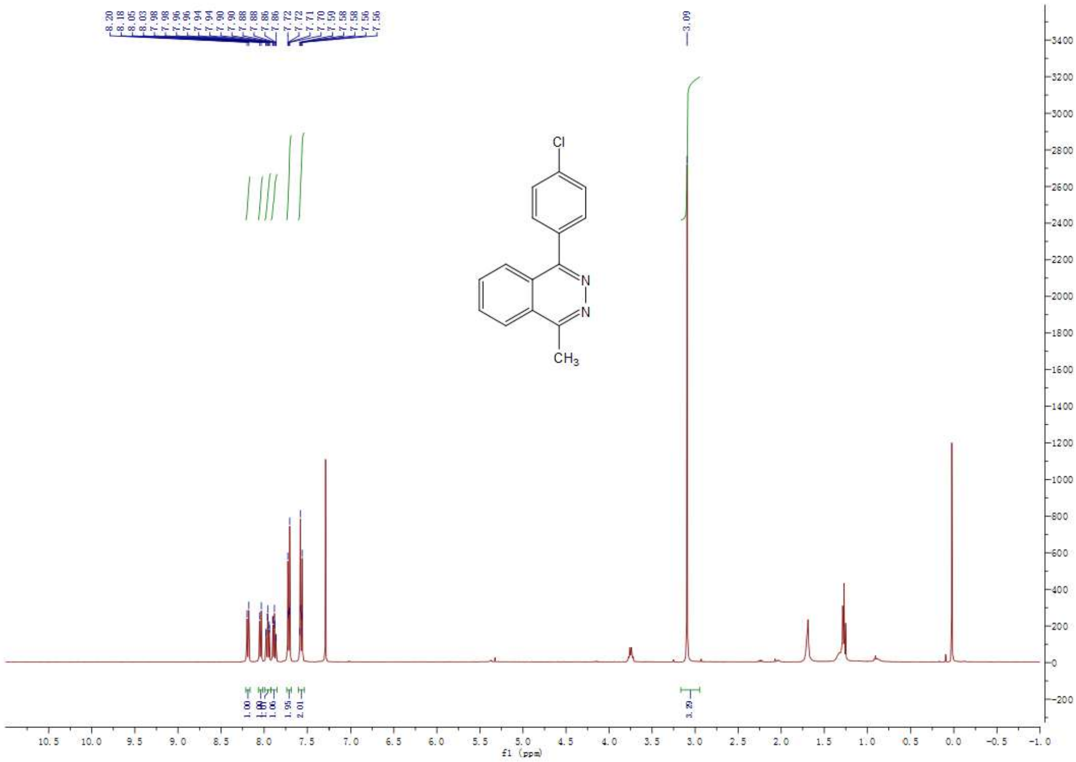

The NMR spectra of 4a

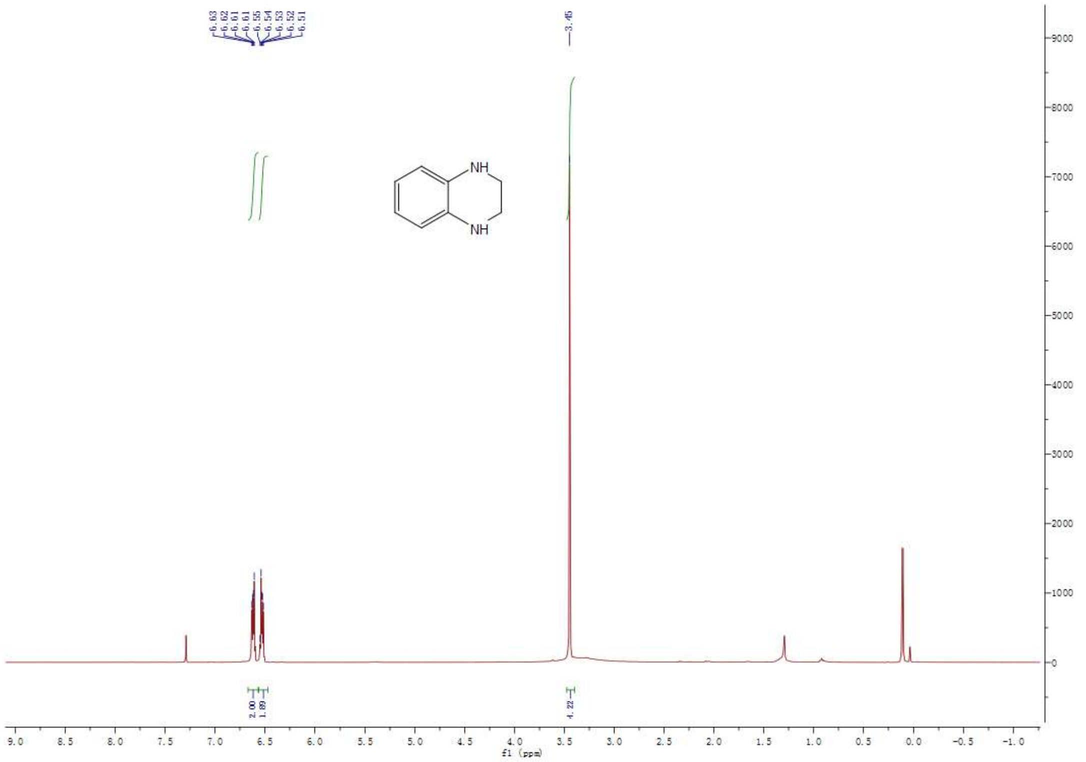

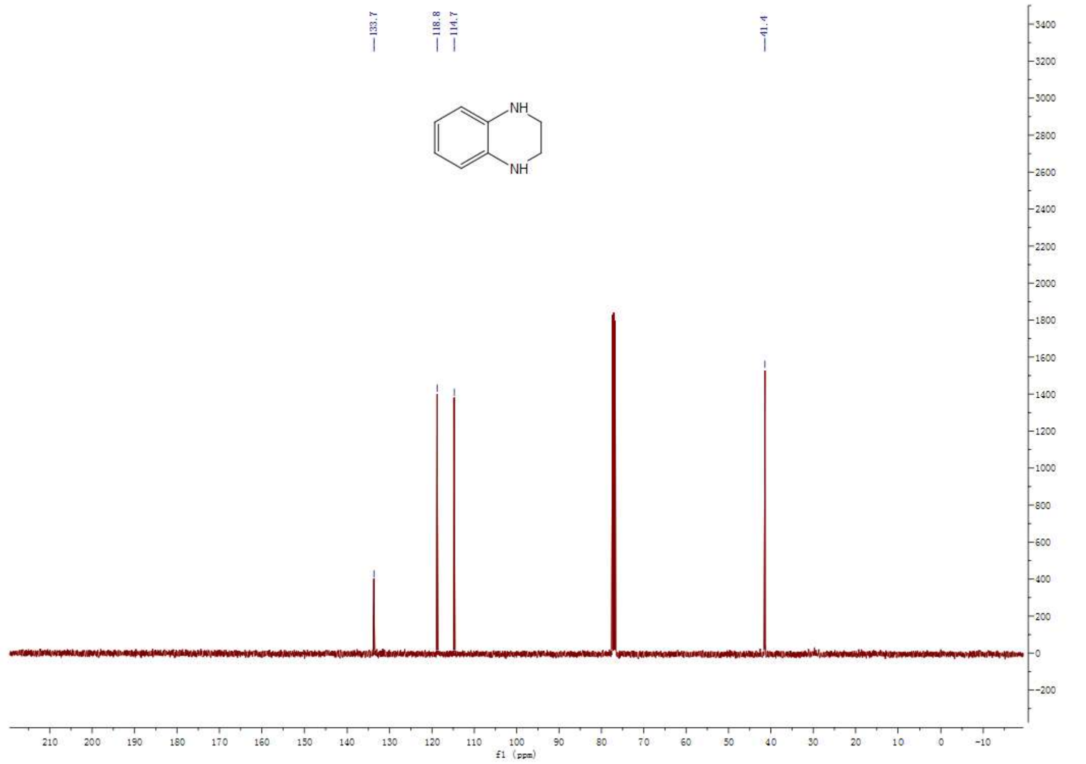

The NMR spectra of 4b

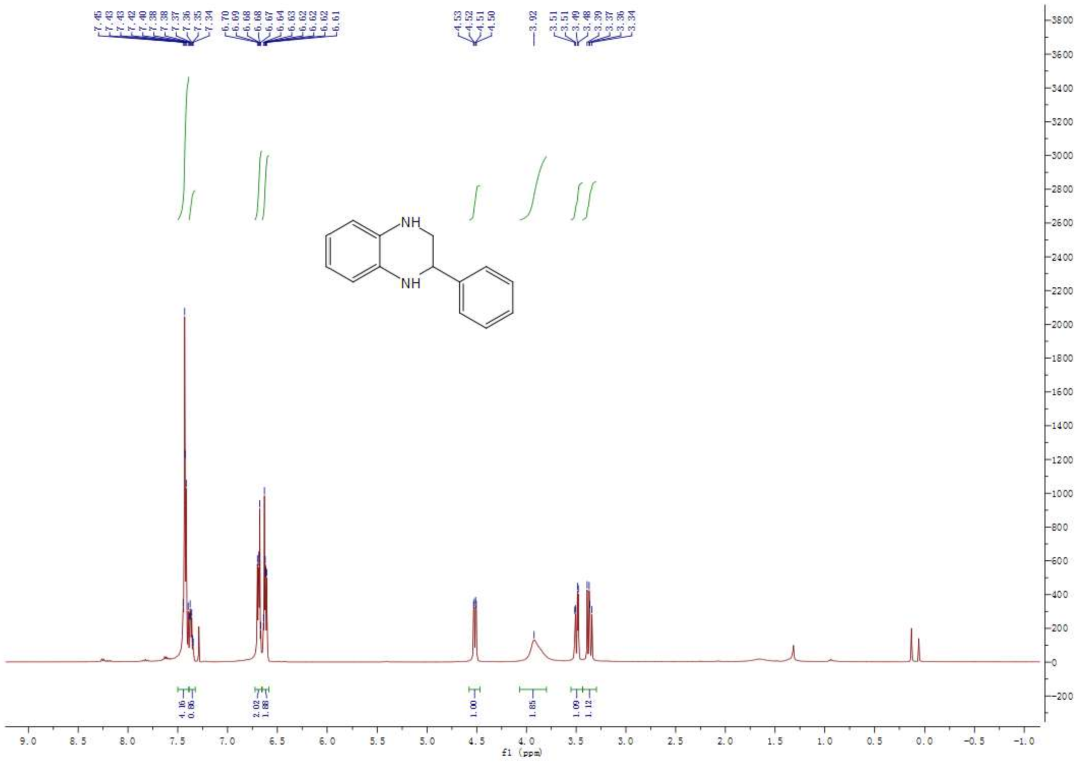

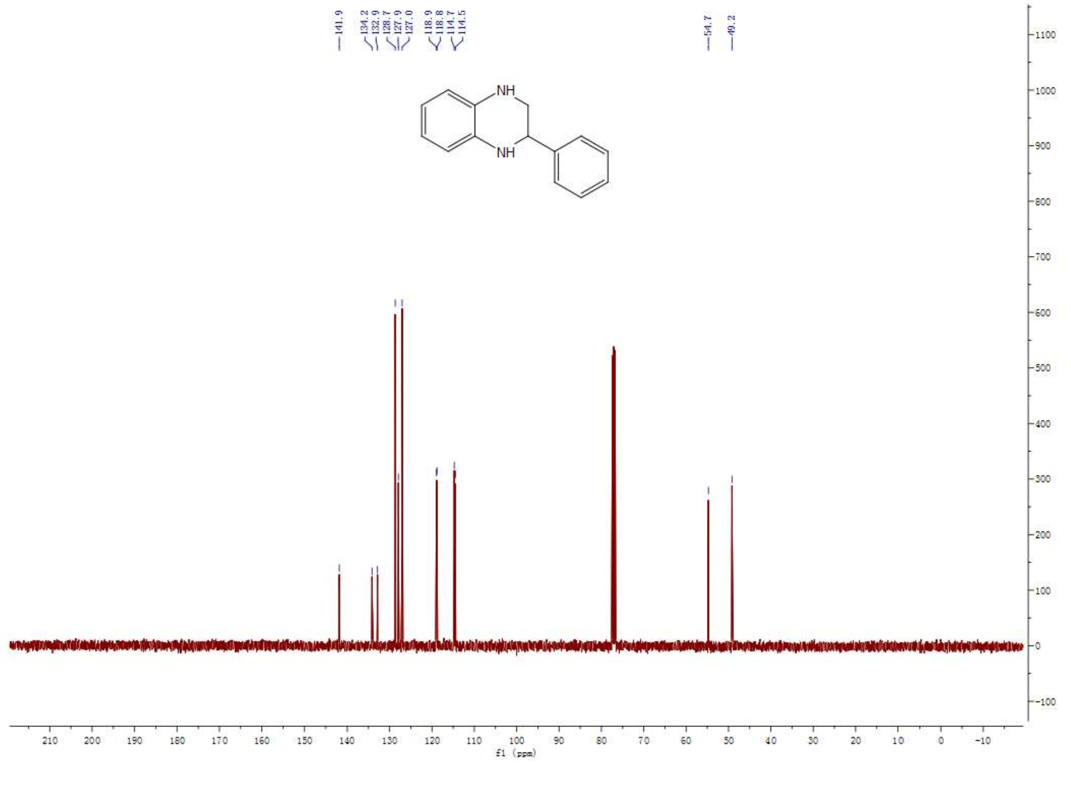

The NMR spectra of 4c

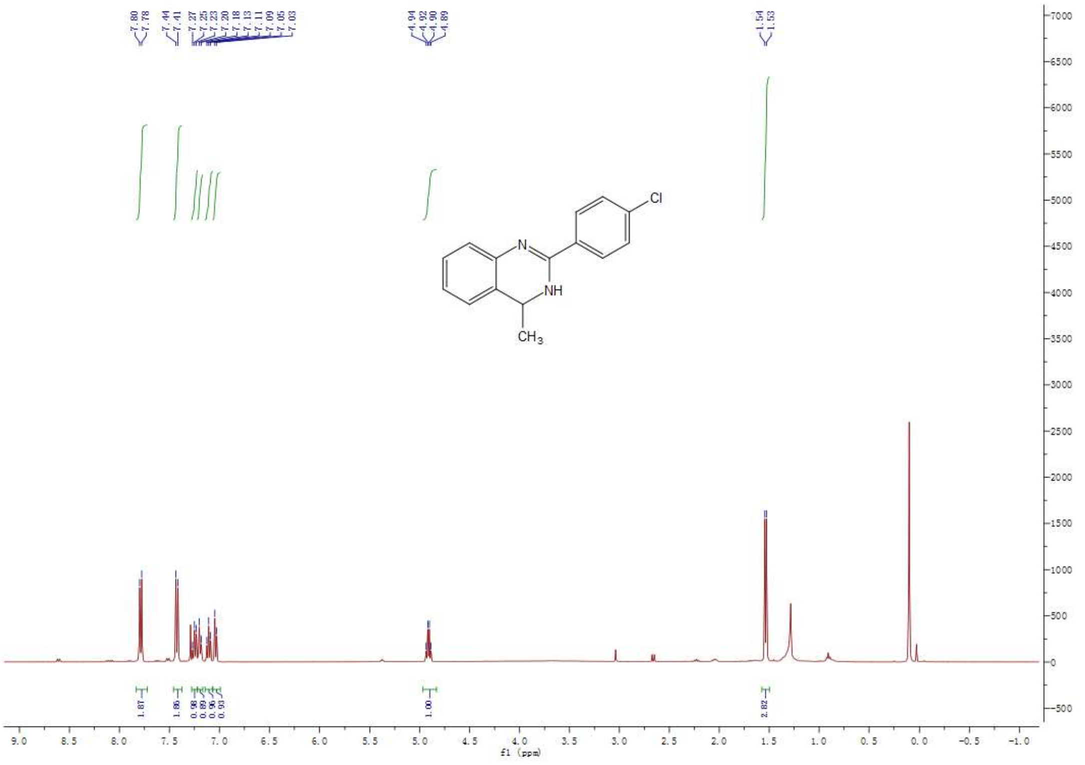

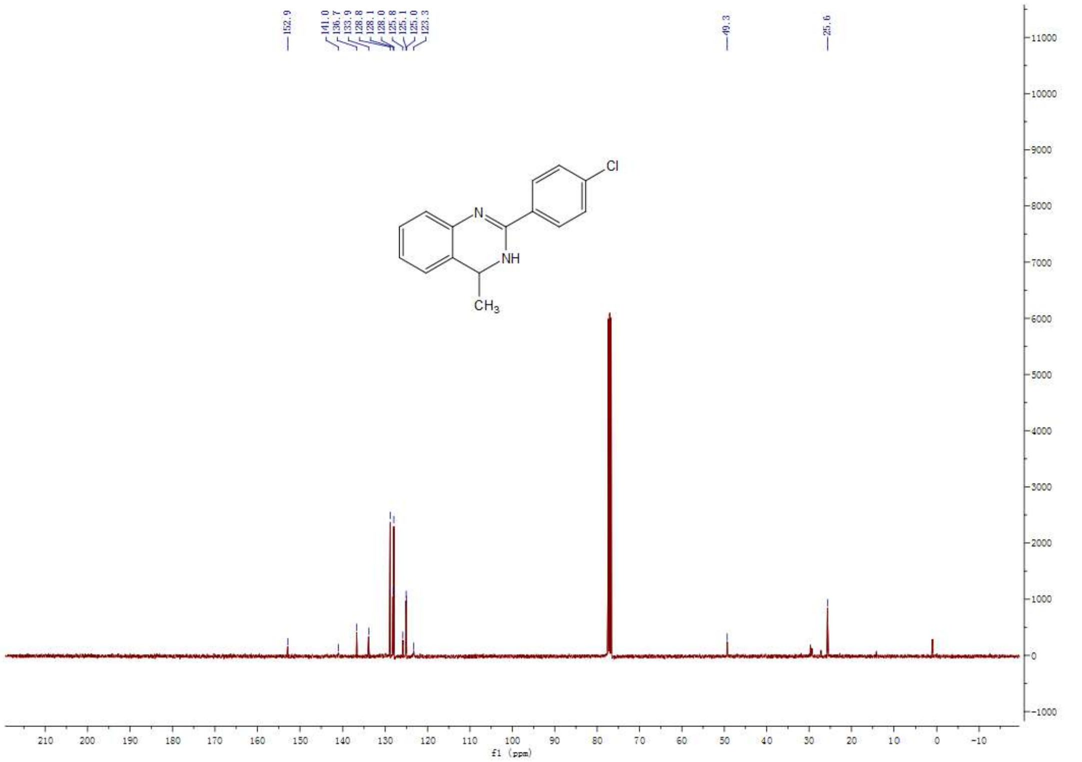

The NMR spectra of 4d

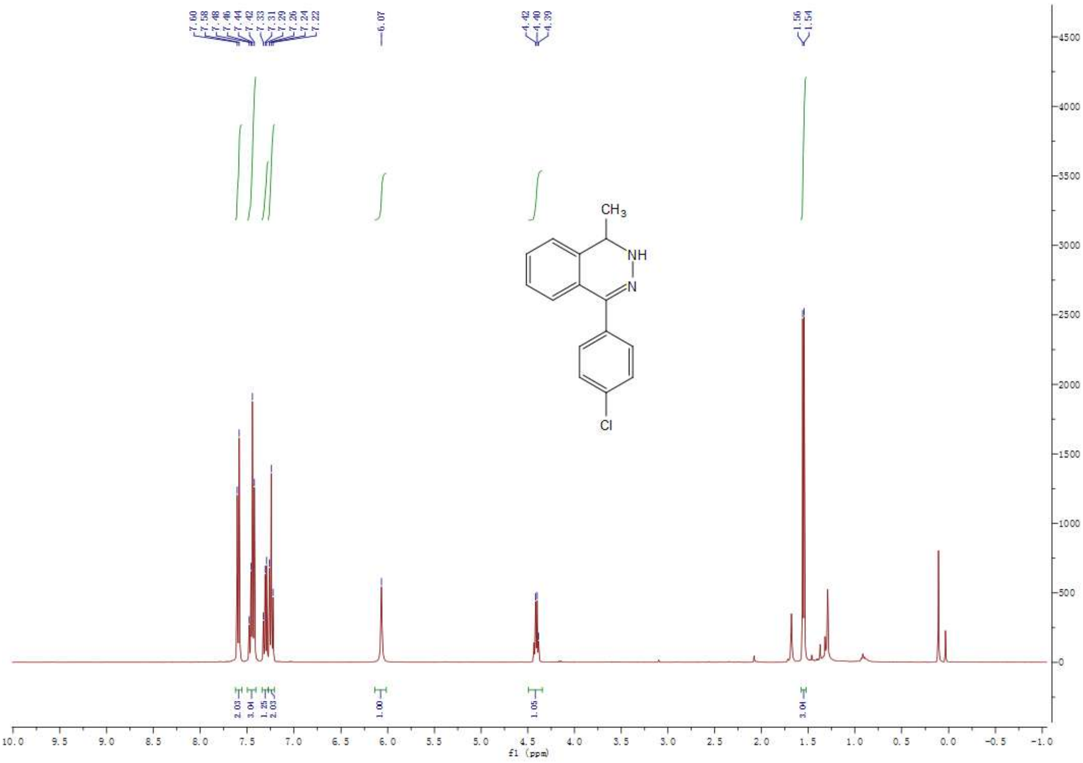

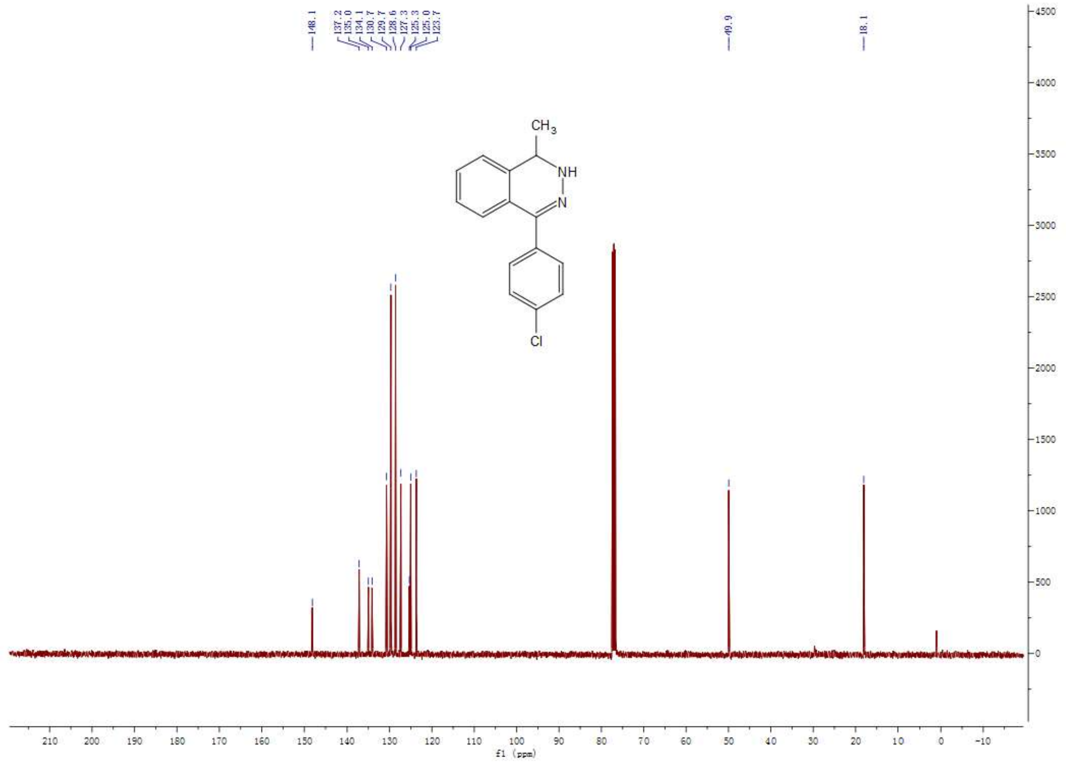

The NMR spectra of 4e

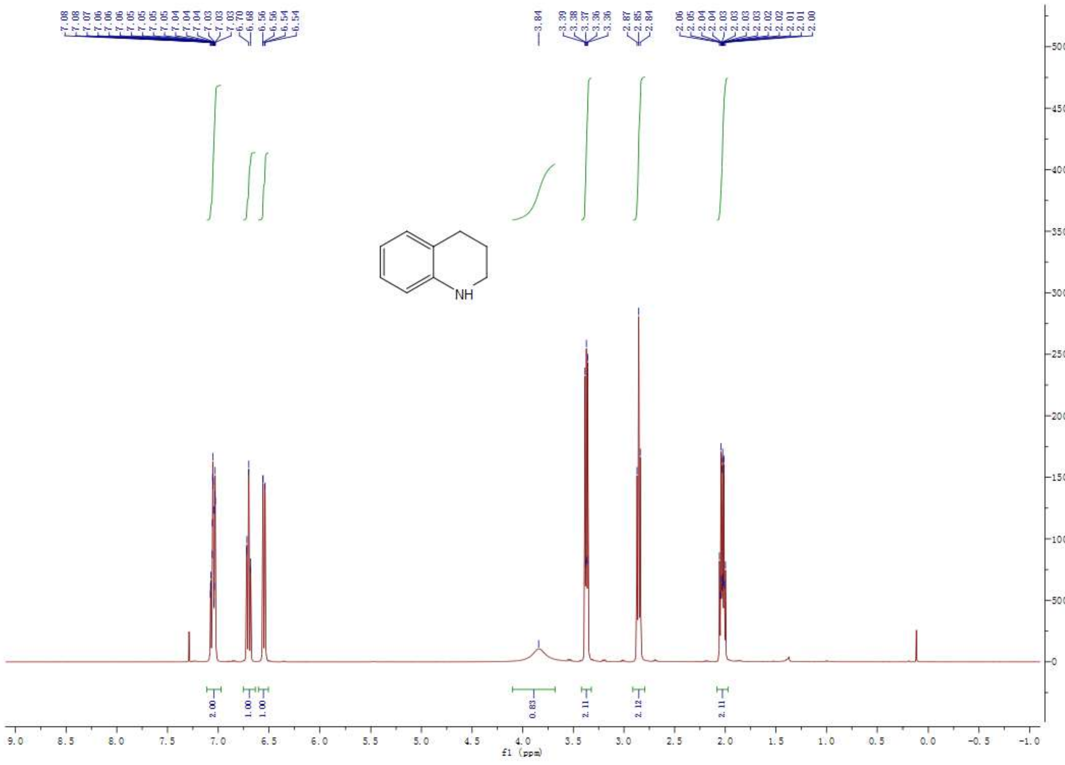

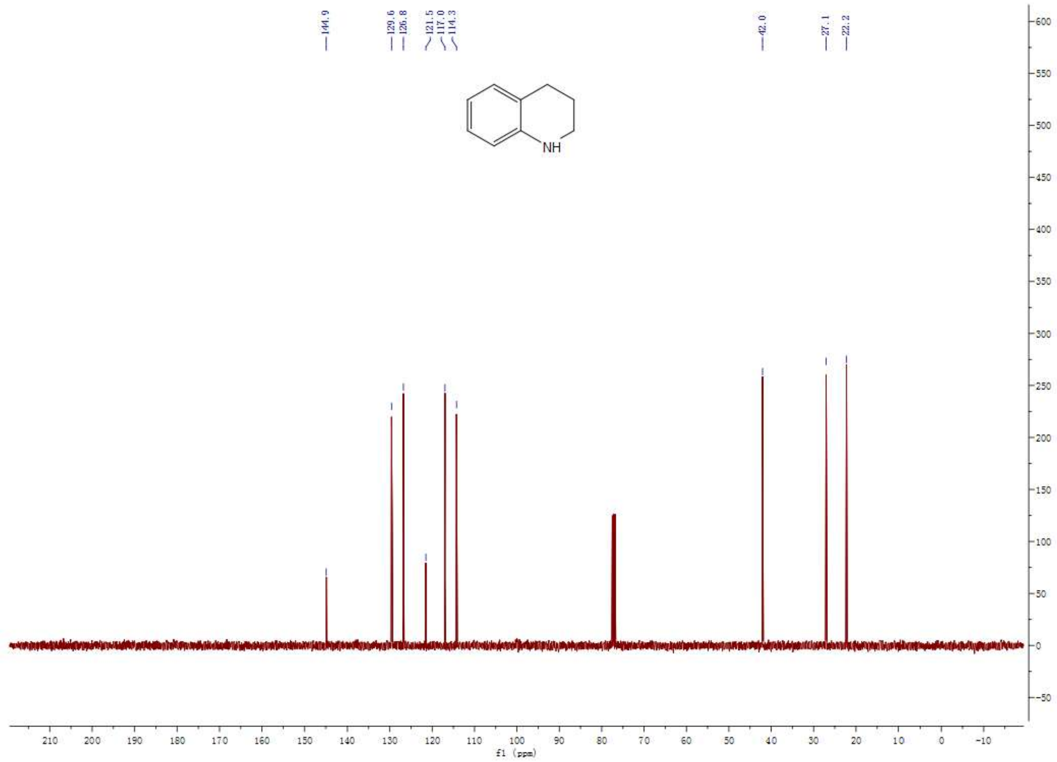

The NMR spectra of 4f

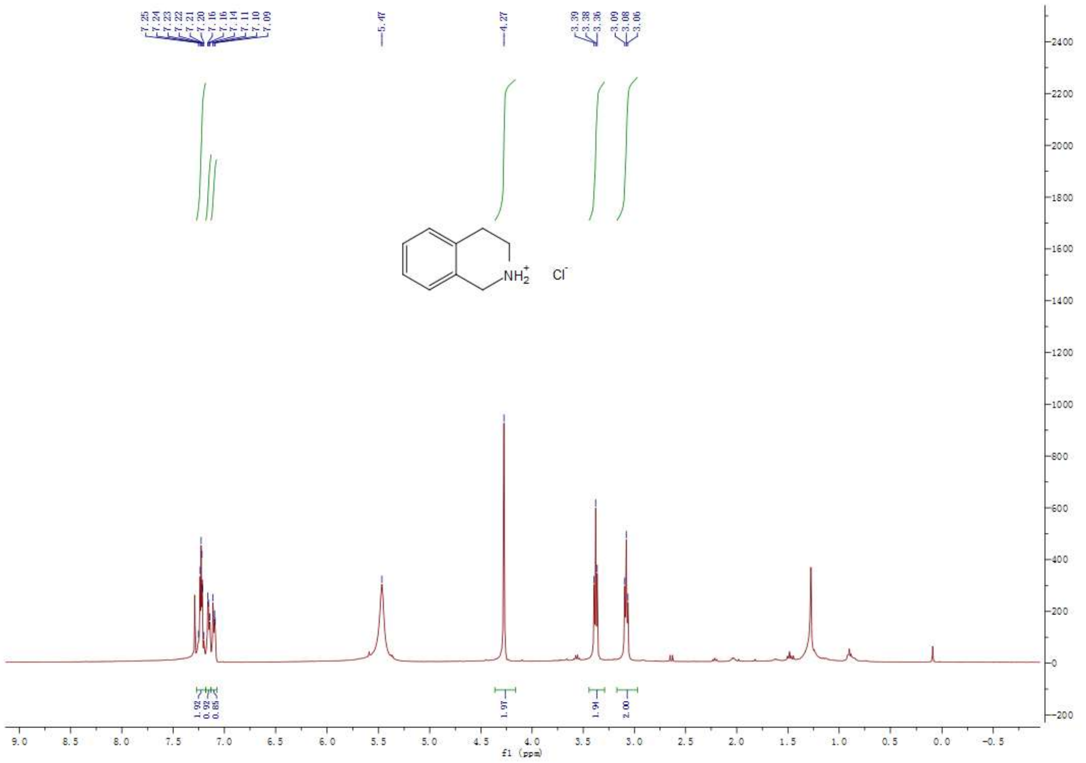

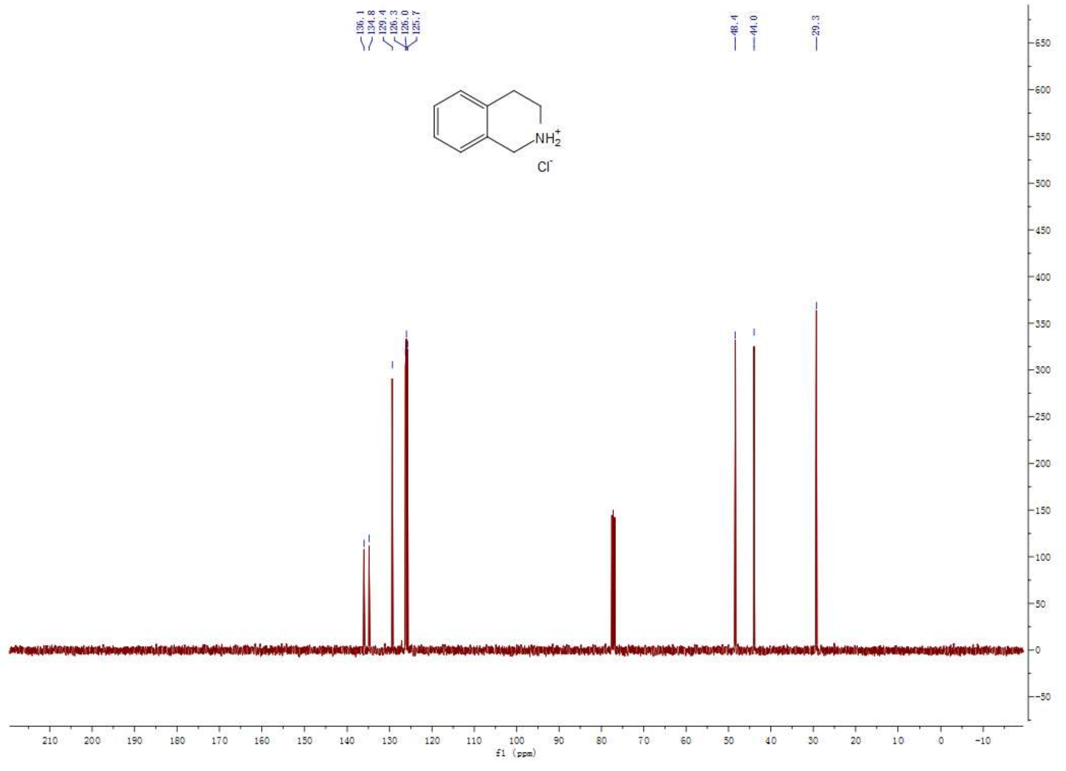

The NMR spectra of 4g

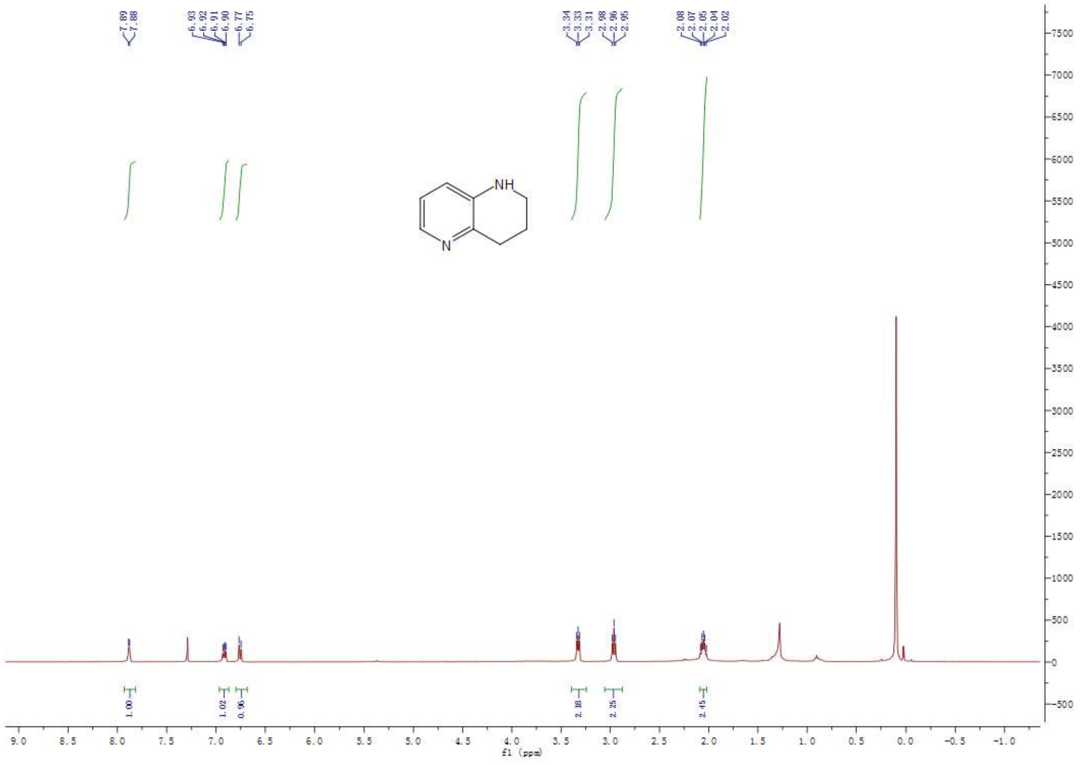

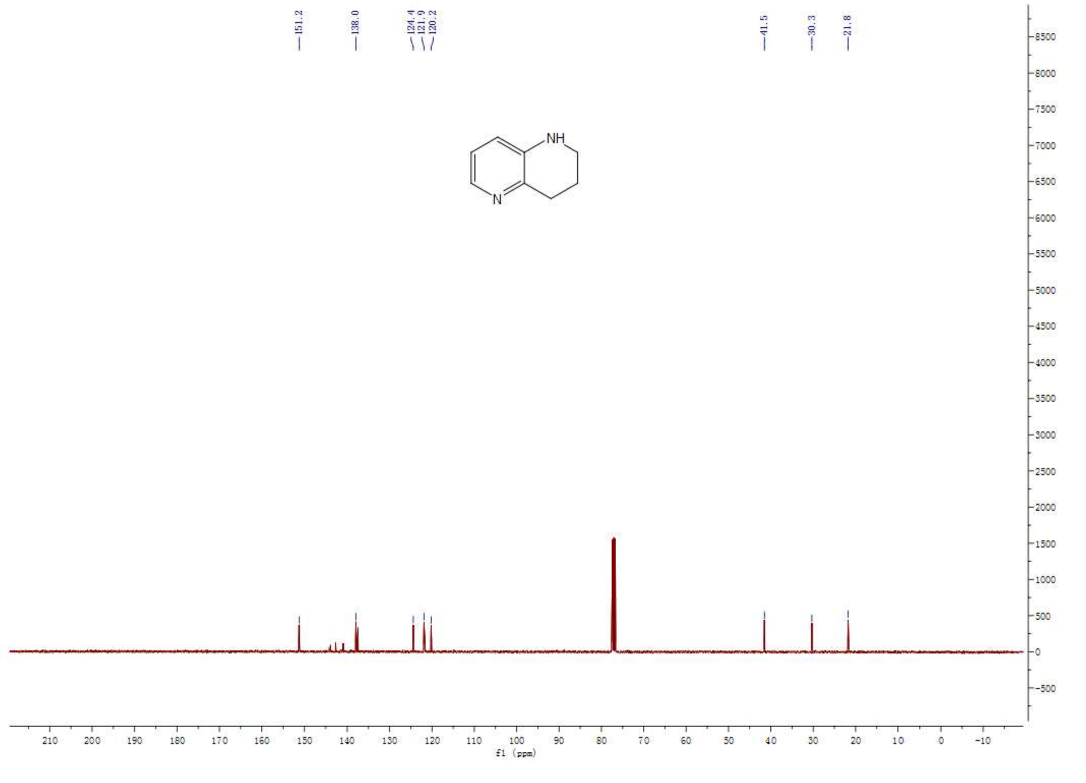

The NMR spectra of 4h

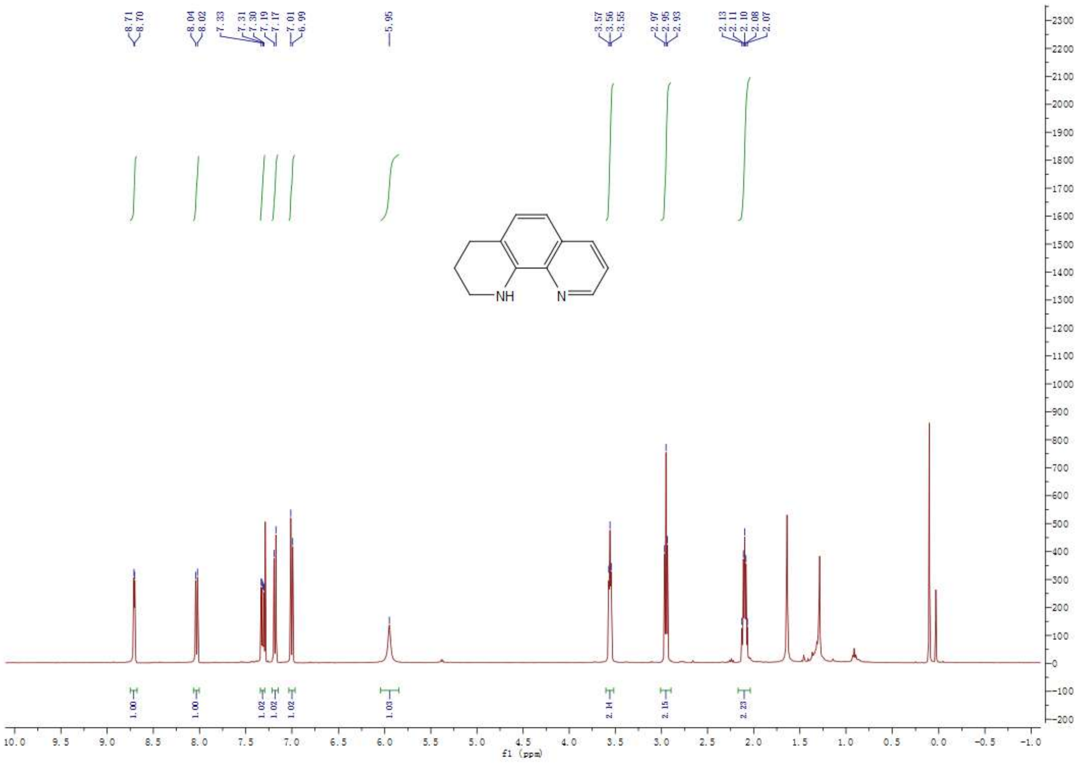

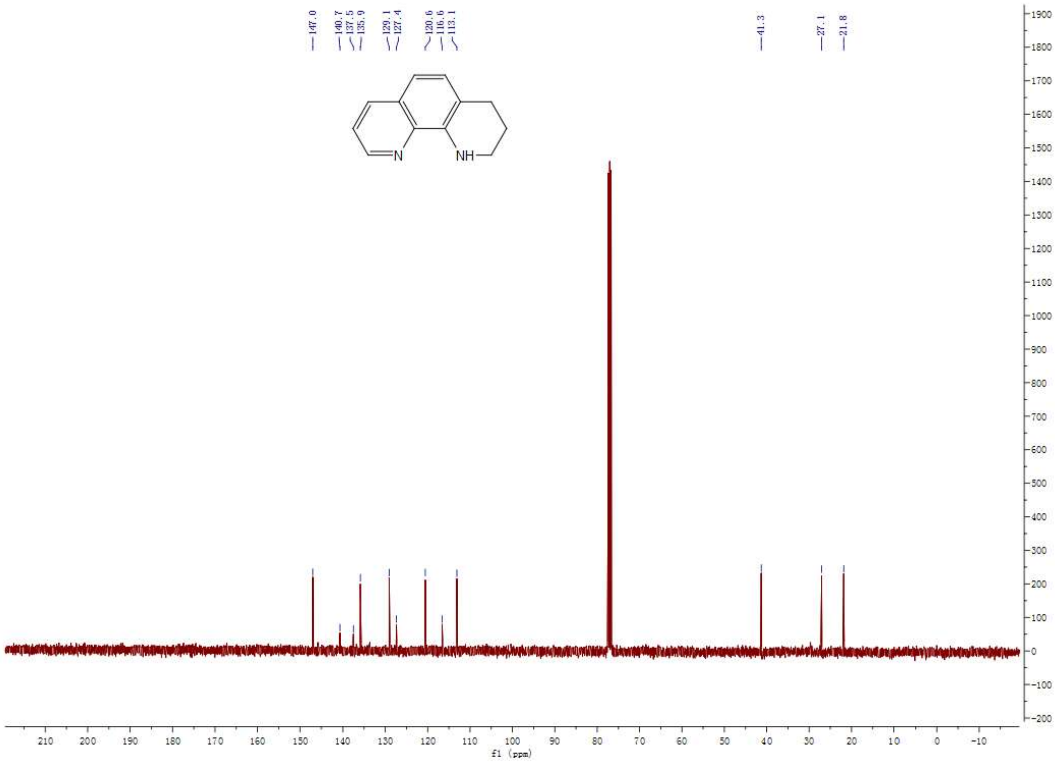

The NMR spectra of intermediate C

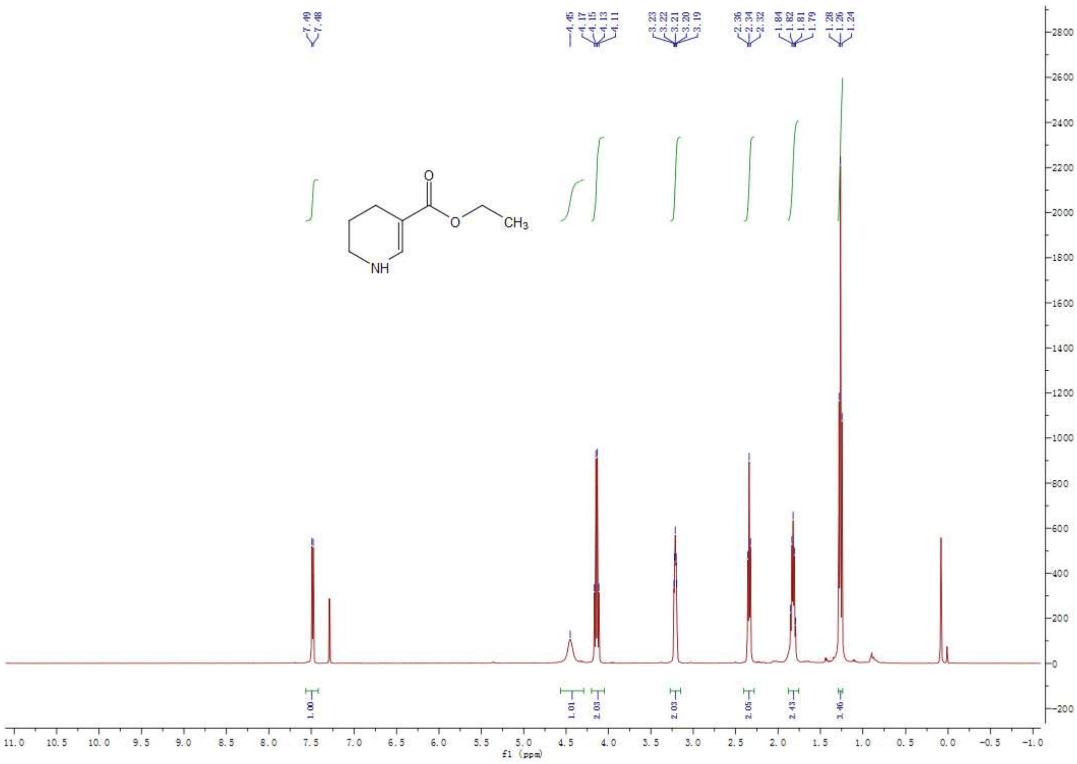

The NMR spectra of 5

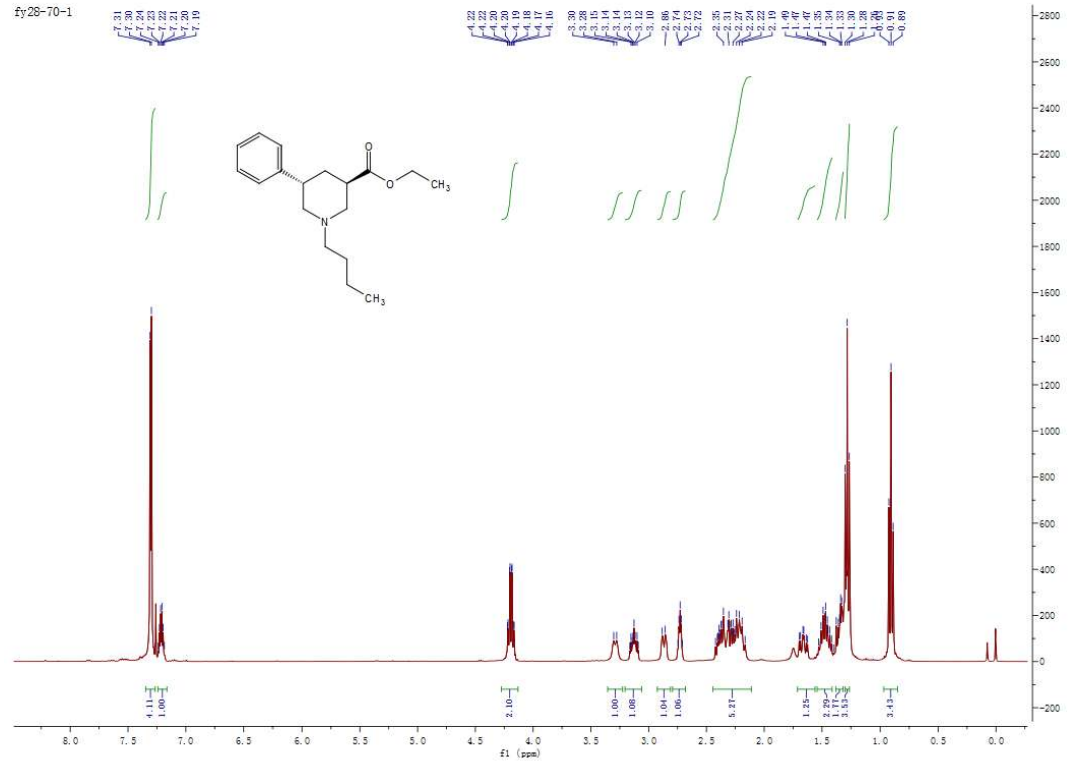

The NMR spectra of 6

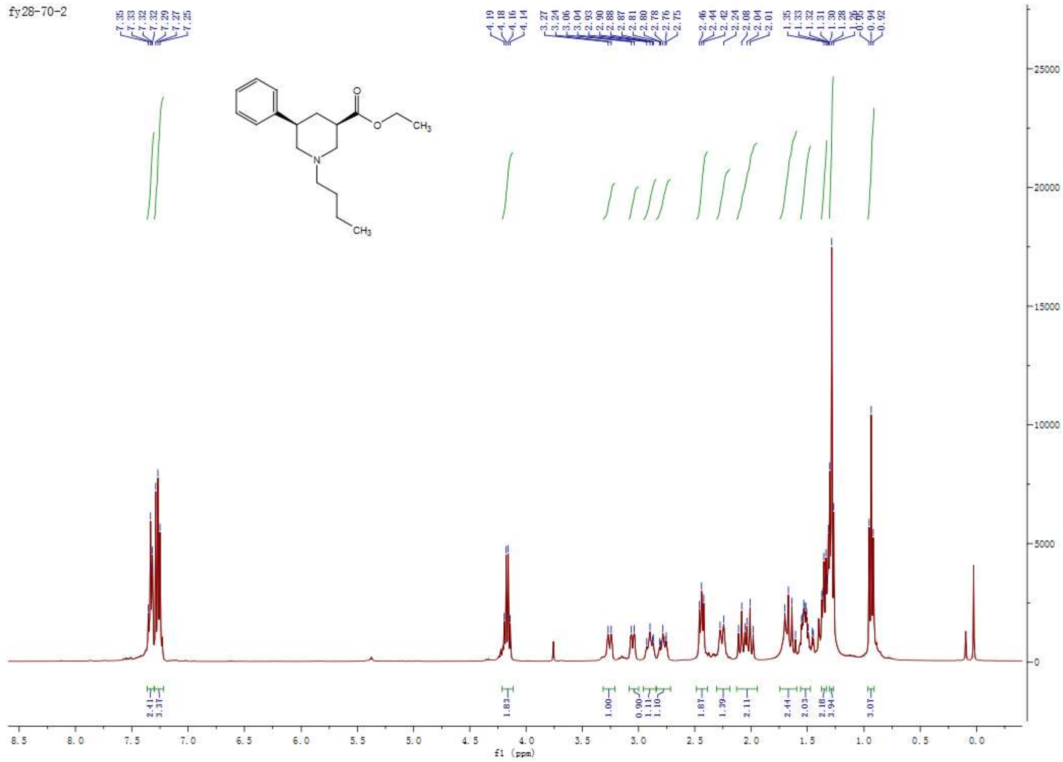

Supplement: Supplementary file 1 [file molecules-24-00401-s001.pdf]
